# Physics-Inspired Equivariant Descriptors of Nonbonded Interactions

**DOI:** 10.1021/acs.jpclett.3c02375

**Published:** 2023-10-20

**Authors:** Kevin K. Huguenin-Dumittan, Philip Loche, Ni Haoran, Michele Ceriotti

**Affiliations:** † Laboratory of Computational Science and Modeling, IMX, École Polytechnique Fédérale de Lausanne, 1015 Lausanne, Switzerland

## Abstract

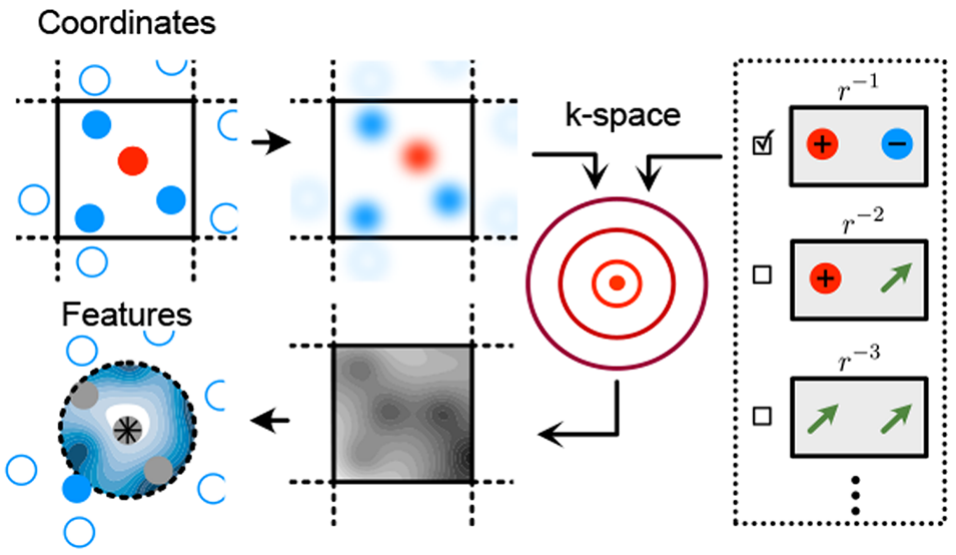

One essential ingredient in many machine learning (ML) based methods for atomistic modeling of materials and molecules is the use of locality. While allowing better system-size scaling, this systematically neglects long-range (LR) effects such as electrostatic or dispersion interactions. We present an extension of the long distance equivariant (LODE) framework that can handle diverse LR interactions in a consistent way and seamlessly integrates with preexisting methods by building new sets of atom centered features. We provide a direct physical interpretation of these using the multipole expansion, which allows for simpler and more efficient implementations. The framework is applied to simple toy systems as proof of concept and a heterogeneous set of molecular dimers to push the method to its limits. By generalizing LODE to arbitrary asymptotic behaviors, we provide a coherent approach to treat arbitrary two- and many-body nonbonded interactions in the data-driven modeling of matter.

Modeling approaches based on machine learning (ML) have become ubiquitous in the field of atomistic simulations, bridging the gap between techniques purely based on classical mechanics using empirical force fields, which are fast but less accurate, and quantum mechanical approaches providing greater accuracy at a larger cost.^1−4^ Since the introduction of early ML models to predict the energy of extended atomic structures^1,2^ and molecules,^5^ the field has rapidly expanded in many directions, improving the accuracy of predictions, and extending the diversity of target properties beyond energies and forces, including vectorial and tensorial properties,^6−8^ electron densities,^9−12^ single-particle electronic Hamiltonians^13,14^ and many-particles wave functions.^15,16^ A key ingredient in most successful approaches has been the use of locality, often justified in terms of the “nearsightedness” of electronic structure.^17,18^ Local models truncate atomic interactions up to a cutoff radius, which allows the development of fast algorithms that scale linearly with the number of particles.

Introducing a cutoff, however, neglects important contributions from all sorts of long-range (LR) interactions. The most prominent LR effects are the 1/*r* Coulomb potential between electrical charges as well as the 1/*r*^6^ dispersion interactions between induced dipoles.^19^ Many other asymptotic decays exist, e.g. charge-dipole and hydrogen bonding (1/*r*^2^), permanent dipole–dipole (1/*r*^3^), and charge-nonpolar (1/*r*^4^)^20^ interactions. On a coarser-grained scale, the effective potential between complex molecules involves different combinations of exponents such as in dense polymer solutions, between particles and surfaces, or membranes in biological systems.^21,22^

At the simplest level, neglecting LR effects sets a lower bound to the possible prediction error. In some cases, LR interactions determine qualitatively different behavior in materials.^23^ In order to address these issues in ML models, several approaches have been proposed. If the goal is to predict the energy and forces of an atomic structure, the simplest strategy is to add an explicitly fitted 1/*r*^*p*^ potential baseline. This idea was already applied to one of the earliest ML potentials^2^ and can easily be combined with any ML scheme.^24−26^ Such a simple approach, in which each chemical species is assigned a fixed point charge, has clear limitations, and more sophisticated techniques have been proposed. One direction is to explicitly include the Wannier centers associated with the valence electrons into the ML framework.^27,28^ Given that the center of charge of the electrons can be different from the positions of the nuclei, these models can describe both the ionic charge and local polarization. In order to describe global charge transfer, without violating charge-neutrality constraints, several methods have been proposed that employ a global charge equilibration scheme, predicting electronegativities rather than the charges,^29−31^ and more general self-consistent treatments of electrostatics.^32^ While the Coulomb potential has been at the center of these developments, some of these methods have also been extended to other interactions, including dispersion^25^ and more general potentials,^33^ often using a rather explicit physics-based functional form. There is, however, a lack of a unifying framework that treats various types of LR interactions consistently. One promising approach to fill in this gap is the Long-Distance Equivariant (LODE) framework,^34,35^ that encodes LR structural data in a form that mimics the asymptotic behavior of electrostatic interactions. Thus, even if LODE features are “physics-inspired”, and can be related to explicit physical interactions when used in a linear model, they retain the full flexibility of general ML schemes.

Here, we generalize LODE features to mimic the asymptotic behavior of any potential with an inverse power law form beyond Coulomb interactions and provide an efficient implementation that also includes gradients of the descriptors with respect to atomic positions, that are needed to compute forces. We provide a detailed mathematical analysis that allows an exact physical interpretation of the resulting LODE coefficients and explain how the framework can also describe many-body effects beyond pair potentials. We finally investigate the subtle balance between physical interpretability and generality of the ML model using two families of data sets: simple toy systems and a diverse collection of molecular dimers.

We first provide a concise summary on the construction of the LODE descriptors as is discussed in refs (36 and 37) and illustrated in Figure 1. A self-contained and more detailed discussion can be found in section S2 of the Supporting Information. The position of the atoms in a structure is encoded in a permutation-invariant way by defining a smooth atom density ρ(***r***)—the same method that is used to define local atom-density descriptors including the popular smooth overlap of atomic positions (SOAP) descriptor (Figure 1a). The Coulomb potential *V*(***r***) generated by this density (Figure 1b) can then be computed efficiently (e.g., with an Ewald summation in reciprocal space^38^). The atom centered features for atom *i* are then generated by first shifting the coordinate system to its position ***r***_*i*_, leading to the atom-centered potential *V*_*i*_(***r***) = *V*(***r* + *r***_*i*_ ). This potential, evaluated up to some cutoff radius *r*_c_, is then projected onto a set of basis functions consisting of (real) spherical harmonics *Y*_*l*_^*m*^ specified by the angular indices *l* = 0, 1, ..., *l*_max_ and |*m*| ≤ *l*, as well as radial basis functions *R*_*nl*_(*r*) for *n* = 0, 1, ..., *n*_max_ – 1 via the integral
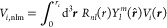
1where ***r*** corresponds to the displacement from atom *i*. Due to the slow decay of the 1/*r* potential, these LODE coefficients contain information on the position of far-away atoms, despite using an environment cutoff for the integration. In fact, similar to a Fourier expansion, knowing all coefficients in the limit as (*n*_max_,*l*_max_) → ∞ would allow one to recover the original function *V*_*i*_, meaning that the LODE coefficients also play a role of fitting coefficients as shown in Figure 1c. They have the same mathematical properties as those used to discretize the local density ρ, and can be used in a similar way, combining them in a symmetry-adapted fashion to obtain invariant features analogous to the SOAP^2,3^ descriptor and its higher-order invariant and equivariant extensions.^4,7,14,39,40^ In particular, it was shown that a combination of local density coefficients and Coulomb-field LODE leads to “multi-scale” features, that, when used in linear models, can be interpreted in relation to the multipole expansion of the electrostatic potential.^35^

**Figure 1:**
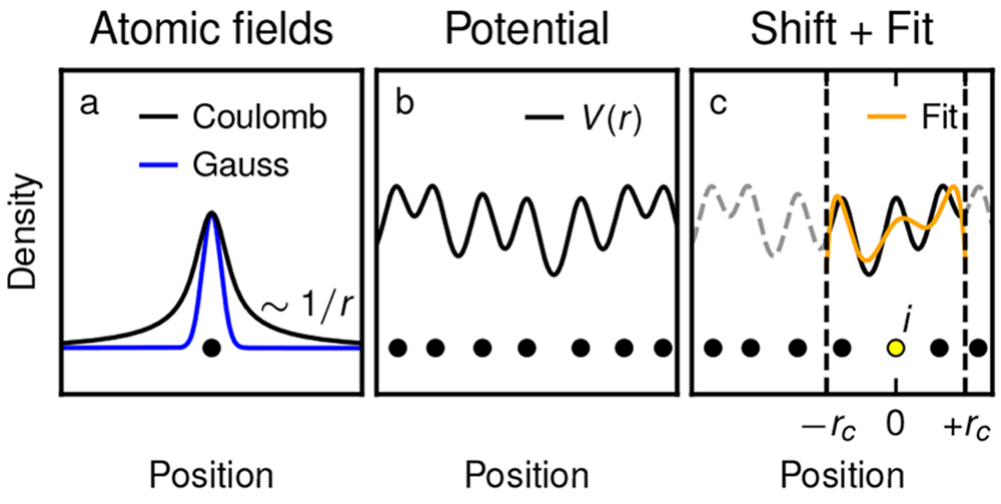
Construction of the LODE features. (a) Gaussian density and the Coulomb potential generated by it, which is a smeared out version of the bare 1/*r* Coulomb potential. The key for the LODE construction is the significantly slower decay of the smeared Coulomb potential. (b) Potential field *V*(*r*) obtained as the superposition of the smeared Coulomb potential on all atoms (black circles). In panel c, *V*_*i*_ is defined as the restriction of *V* to a local environment around the position of atom *i* (yellow circle), where the coordinate system has also been shifted. *V*_*i*_ is then discretized by using atom-centered basis functions. The resulting coefficients, obtained from eq 1, are the LODE features.

As a first key result in this work, for which we provide a detailed derivation in sections S1 and S2 in the Supporting Information, we present an exact physical interpretation of the LODE cofficients *V*_*i*,*nlm*_. For a fixed center atom *i*, the potential *V*_*i*_^>^(***r***) around atom *i* generated by the charge density *outside* the cutoff region can be shown to be of the form
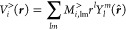
2where the coefficients *M*_*i*,*lm*_^>^ are called (exterior) multipole moments and completely characterize the potential generated by the exterior atoms as discussed in more detail in section S1 of the Supporting Information. The main result here is that with an appropriate choice of radial basis, the LODE coefficients are precisely *equal* to the multipole moments, *V*_*i,nlm*_ = *M*_*i,lm*_^>^, and in particular contain the full information on the exterior atoms for what concerns electrostatic properties. Furthermore, our derivation shows that one does not need a large number *n*_max_ of basis functions, since eq 2 does not depend on an index *n*. By choosing *R*_0*l*_(*r*) = *r*^*l*^, a single monomial basis function per angular momentum channel *l* is sufficient to capture the electrostatic contributions from far-field atoms. This translates into computational savings by a factor of *n*_max_, which varies between 4 and 12 in typical applications. We will call this the monomial or optimized basis, since this is the most compressed form in which we can store the exterior electrostatic information.

A potential risk in previous LODE implementations,^34,35^ that are fine-tuned to capture electrostatic behavior, is that they will be less flexible in describing other LR interactions. To address this issue, we generalize the LODE construction to arbitrary 1/*r*^*p*^ interactions, leading to a *p*-dependent family of potentials *V*^(*p*)^. While conceptually straightforward, this change does come with several subtleties. First, a key ingredient to obtain well-defined coefficients in the Coulombic case was the use of Gaussian charge densities rather than point charges to remove the singularity of 1/*r* at the origin. For *p* ≥ 3, the divergence as *r* → 0 becomes so strong that even using a Gaussian smearing, the resulting potential is still singular. Thus, a family of effective potentials parametrized by *p* and having the correct 1/*r*^*p*^ behavior for large distances is used instead of the bare form.^38,41,42^ The second difference is that the multipole expansion for a 1/*r*^*p*^ potential with *p* ≠ 1 contains several terms that do not appear in the Coulombic case. Despite these additional complications, all results can still be translated to the general case with suitable modifications. Both of these subtleties are discussed in sections S2 and S3 in the Supporting Information. While these results apply to interactions that can be treated as pair potentials, the LODE framework can also describe a wide range of LR many-body interactions by combining multiple LODE coefficients to generate higher order invariants. This approach, which we discuss in more detail in section S4 in the Supporting Information, leads to the LR analogue of systematic body-order expansions used in methods based on atom-centered density correlations,^39^ including ACE^40^ or NICE^43^ models.

To assess the capabilities of the general LODE framework, we begin with a demonstrative example, using a modified version of the toy data set originally presented in ref (35). It consists of 2000 structures, each obtained by distributing at random 64 particles inside cubic cells of varying size such that no particles are closer than 2.5 Å. For the same particle positions we consider two different potentials: in one case, similar to ref (35), we treat the structures as an overall neutral cloud of ±1 charges. In the second test, all atoms are equal and interact via an attractive 1/*r*^6^ dispersion interaction. Periodic boundary conditions are used in both cases.

The energies of these systems are learned using linear models built on either short-range (SR) or LODE descriptors with *p* = 1 and *p* = 6. The latter are computed with a method similar to Ewald summation using a reciprocal space sum, matching the periodicity of the target system. See section S8 in the Supporting Information for implementation details. For the LODE descriptors, we only take the coefficients for which *l* = *m* = 0, and compare two choices of radial basis functions *R*_*nl*_. As a baseline, we use the Gaussian-type orbital (GTO) basis functions used in ref (35) for both SR and LODE models for this test, with *n*_max_ = 8 radial basis functions. This is compared to the optimal radial basis that uses a single basis function. In fact, for *l* = *m* = 0, both the spherical harmonic  and *R*_0*l*_ = *r*^*l*^ = 1 are just constant functions. Thus, it can be seen from eq 1 that the coefficient *V*_*i*,000_ that enters the model is, up to a global factor, the average within the cutoff sphere of the potential generated by the atom density. For sufficiently small cutoff radii, we therefore simply recover the potential at the position of atom *i*. Model details and parameters can be found in section S5 of the Supporting Information.

The results in Figure 2 show the percentage root mean square error (%RMSE), defined as the absolute RMSE divided by the standard deviation σ of the energies in the training set, against the number of training set structures. We observe that the SR descriptors, even when using a large 9 Å cutoff, are unable to learn electrostatic or dispersion interactions, leading to rapidly saturating learning curves. On the other hand, the results show clearly how using a generalized LODE descriptor adapted to the asymptotic decay of the LR potential allows one to learn the target potential with very high accuracy and very few training structures. By using the optimized radial basis, a single LODE coefficient can reach the same accuracy of a model using an expansion on *n*_max_ = 8 GTO basis functions.

**Figure 2:**
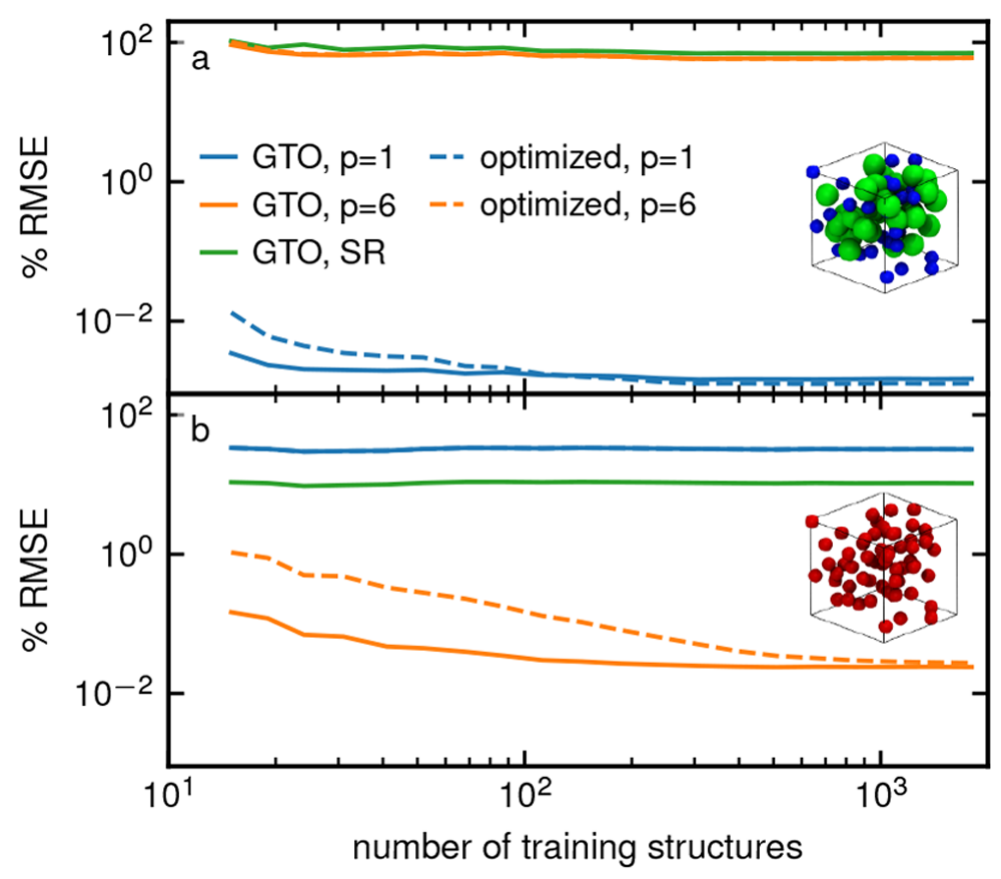
Learning curves showing the validation % RMSE against the number of training structures. In part (a), half of the particles each have a charge of ±1, while in part (b), all particles are neutral and interact solely via an attractive dispersion interaction. Solid lines show models using the GTO basis with *n*_max_ = 8 coefficients, whereas dashed lines use the optimized basis with a single coefficient. To convert back to absolute errors, the standard deviations in the energies are σ = 1.15 eV/atom and σ = 0.14 eV/atom, respectively.

In light of the analytical results discussed above, these observations are unsurprising. In fact, it is more insightful to comment on the residual errors. First, it is clear that the choice of a physically constrained model affects its applicability to other types of interactions. The performance of a *V*^(1)^ linear model for a 1/*r*^6^ potential, or those of a *V*^(6)^ for 1/*r* are comparable or worse to those of a SR model. Second, interaction-adapted models achieve high accuracy, but there is a residual error, which is due to the finite Gaussian smearing of the atoms and convergence of the reciprocal space sum in the implementation. While Ewald-based methods in classical molecular dynamics, including PME,^44^ SPME,^45^ and P3M^46^ correct for the smearing by using a compensating SR part,^47^ the LODE descriptor itself does not contain such corrections. This would not be an issue in practice, however, since one typically combines LODE with SR representations for more complex systems. The slightly slower convergence of the optimized radial basis can also be explained by the finite Gaussian smearing, and the cutoff radius for the integration in Equation 1, leading to discrepancies between the true potential and the LODE coefficients that can be compensated with a sufficient amount of data and/or fitting coefficients.

From this first example, we conclude that a single *l* = 0 component of the extended LODE framework (1) is sufficiently expressive to learn a LR potential with matching exponent with high accuracy and (2) has an increased efficiency due to a better choice of radial basis. Beyond such pair or two-body potentials, we also show in section S4 how higher order invariants built from the LODE coefficients can be used to learn three-body dispersion, supporting the more general result that the framework is also capable of treating many-body LR effects. Armed with descriptors that have a rigorous physical interpretation but can be easily combined with arbitrary ML frameworks, we can now explore the interplay between descriptors, model architecture, and type of LR interactions using a more challenging data set, with target properties obtained from actual quantum mechanical methods.

To cover a wide range of interactions between sufficiently simple but relevant molecules, we base our tests, on the BioFragment Database (BFDb),^35,48^ that contains 2291 pairs created from 22 relaxed organic molecules. For each configuration, we evaluate energies and forces along binding curves built starting from the configuration included in the BFDb with initial separation *r*_0_, and increasing the separation along the line connecting the centers of mass (COM) of the two molecules, up to *r* = 15 Å between the COMs. We note that (1) we use the HSE06 hybrid functional^49^ with a high fraction of exact exchange, to reduce the DFT localization error and ensure that charged dimers dissociate without spurious fractional charges, (2) we include a nonlocal many-body dispersion correction^50^ and (3) we use a supercell approach, so that binding energies also contain interactions between periodic replicas, consistent with the reciprocal-space calculation of LODE descriptors. Full details for the data set construction and the models are provided in section S6 of the Supporting Information.

The BFDb contains charged molecules, polar molecules with an effectively constant dipole moment, and apolar molecules without any permanent charge or dipole moment. The combination of these three molecular categories results in six dimer classes with different ideal power-law decay constants of their interactions:^21^ charge–charge (CC) with *p*_CC_ = 1, charge–polar (CP) with *p*_CP_ = 2, polar–polar (PP) with *p*_PP_ = 3, charge–apolar (CA) with *p*_CA_ = 4, polar–apolar (PA) with *p*_PA_ = 5, and finally apolar–apolar (AA) with an ideal interaction decay of *p*_AA_ = 6. We show an example snapshot for a CC pair in the inset of Figure 3c and example energy and force binding curves are shown in Figure S8 of the Supporting Information. We note in passing that often the decay exponents for the computed binding curves deviate from the ideal values, although the general trend of faster decay when moving from CC to AA dimers is preserved (see Figure S8 in the Supporting Information). Even though this data set is very similar to that used in refs (34 and 35), we more directly probe the ability of LR models to capture the tails of different types of interactions and also include forces to the data set. We split the structures into train and test sets based on a threshold separation distance *r*_train_ measured from the shortest separation *r*_0_; i.e., we train on shorter-range information and assess whether the model can extrapolate the asymptotic decay. In addition, we include the dissociated limit of the vanishing interaction energy, where the supercell contains only one monomer. This setup is consistent with typical scenarios, in which one would like to train the ML models on small simulation cells and use them on larger structures that are inaccessible to fully quantum mechanical methods due to the high computational cost.

**Figure 3:**
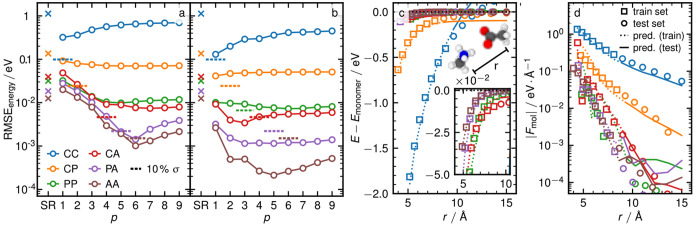
(a) Energy RMSE for models using a single monomial basis as a function of the potential exponent *p* for the six different dimer classes. Lines between the open circles serve as a guide to the eye. The dashed horizontal lines indicate 10% of the standard deviation σ of the training energies for each subset, which indicates the energy scale of each type of interactions. All lines are color-coded based on the type of dimers, as indicated in the legend. (b) Same as in panel (a but using multiple radial basis functions (*n*_max_ = 6, *l*_max_ = 4). (c) Binding energies *E* – *E*_monomer_ as a function of distance *r*. Open squares show data taken for the train set while open circles show data from the test set. Lines show the predictions of the model. For each dimer class, the exponent *p* with the highest prediction accuracy in panel (b) is used. The representative dimer is chosen such that its prediction errors are closest to the average behavior. The inset shows a snapshot of a positively charged methylamine and a negatively charged acetate at a center of mass distance of *r*_0_ = 4.6 Å. (d) Molecular force |*F*_mol_| of one molecule as a function of distance *r*. Symbols and curves are chosen in the same manner as in panel c.

As a first experiment, we fit each dimer class separately, using linear models based on a multiscale power spectrum^35^ that consists of suitable rotationally invariant products combining both SR descriptors and LODE using a single exponent in the range *p* = 1, 2, ...9. The linear model details and fitting procedures are discussed further in section S6D of the Supporting Information. We analyze the performance of the resulting models in Figure 3, for a training threshold of *r*_train_ = 4 Å. The qualitative observations for other threshold distances, shown in Figure S10 of the Supporting Information are similar. Figure 3a shows the test-set energy RMSE as a function of the model’s potential exponent *p* for each class of dimers, using an optimized radial basis for *l*_max_ = 1. In all cases, SR models show poor performance, while the LODE models with appropriate exponents can lead to dramatic improvements by up to 1 order of magnitude. For example, for the CC pairs we find the best model for *p* = 1, while *p* = 6 perform best for the AA pairs. The optimal values roughly correspond to the ideal exponents for each dimer class. The fact that the variation is somewhat smooth can be understood based on the fact that (1) the model potential is built as a superposition of atomic contributions. Over a finite distance interval, any 1/*r*^*p*^ potential can be fitted reasonably well with a superposition of multiple 1/∥ ***r*** – ***r***_*i*_∥^*p*′^ potentials with origins shifted to the atomic positions, even if the exponents *p* ≠ *p*′ do not agree (see section S7 in the Supporting Information for a more in-depth discussion). (2) Binding curves for real molecules do not exactly match the ideal behavior, and (3) interactions with *p* > 1 require more than a single monomial basis. Indeed, considering multiple monomial basis functions (*n*_max_ = 6, *l*_max_ = 4) changes the performance curves (Figure 3b). There is a large improvement for dimer classes with a large *p* (which is consistent with significant contributions from several monomial coefficients), but also for *p* = 1. The dependency of the accuracy on the LODE exponent is also less sharp, which is consistent with our theoretical analysis, which shows that higher-*l* basis functions lead to terms that decay as 1/*r*^*p*+*l*^. It should be noted that for the optimal exponents, the results are best for the AA and PA subsets, which is in part due to the larger size of the training set (according to Table S1 around 6-fold for AA compared to CC). In addition, the training is done on the total energy and forces, which also include SR terms and prove difficult to fit with this data set that is entirely focused on LR contributions. This comparison underscores a key observation in this work: LR interactions in realistic systems cannot be fully captured by the idealized asymptotics. Still, LODE features with an exponent adapted to the dimer class usually *do* perform better, which testifies to the added value of using a physically interpretable framework.

Given the ability of LODE features to target different types of interactions, it is interesting to investigate a model trained against the entire data set. This is particularly challenging because of the vastly different energy scales of the interactions, which is apparent in Figure 3, and because it is more difficult for the model to infer the correct asymptotic behavior of the interactions based on SR training data. A linear model based only on *p* = 1 coefficients yields respectable performance for all dimer classes *separately*, but when applied to the full data set the errors increase by an order of magnitude or more (Figure 4). When compared with the intrinsic energy scale, results are particularly poor for the faster-decaying interactions that are completely overshadowed by the much stronger variability in the CC binding curves: in terms of absolute errors, the error on the LR part for PP, PA, and AA dimers is comparable to that on the CC subset.

**Figure 4:**
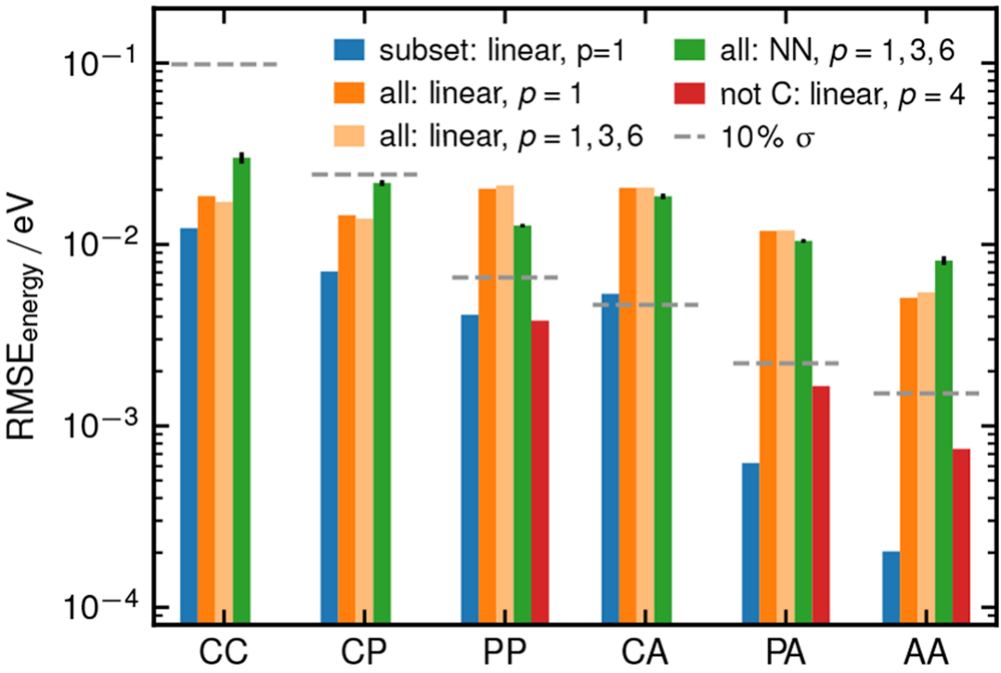
Energy RMSEs for the different subsets of the dimers. Blue bars show the RMSEs of models using a single *p* = 1 LODE exponent similar to that in Figure 3a, but fitted only on energies. Orange and light orange bars show linear models fit to the whole data set, either only using *p* = 1 or combining *p* = 1, 3, 6 for the features. Green bars show a fit to the whole data set with a nonlinear neural network model, again with features combining *p* = 1, 3, and 6. Red bars correspond to a linear model restricted to noncharged fragments, using *p* = 4 (see section S6D of the Supporting Information). Horizontal gray lines depict a relative error of 10% σ for each subset.

The overwhelming dominance of strong interactions cannot be addressed by using more flexible models that include multiple exponents, nor by adding a nonlinear layer on top of the LODE features (Figure 4, light orange and green bars). A more effective strategy, instead, is to reduce the variability in the energy scale by restricting the model to the dimer types that do not contain charged residues (Figure 4, red bars). This reduces the errors on PP, PA, and AA dimers by an order of magnitude. Larger training sets might allow to increase the accuracy when targeting all types of nonbonded interactions. However, our computational experiment highlights one of the inherent challenges when extending ML potential to LR physics. Without including ad hoc structures, and designing training targets that single out the desired type of contributions, fitting models on total energy and forces focuses on the larger SR or electrostatic terms. Other nonbonded interactions that are important to drive collective effects, but small in absolute magnitude, risk being completely neglected even with a model based on physically inspired terms.

Summarizing our results, we present an extension of the long-distance equivariant framework to arbitrary potential exponents and give a direct physical interpretation of the LODE coefficients. We prove a direct link between LODE features and the multipole expansion, which we use to propose a physically motivated radial basis that is adapted to the modeling of the far-field contributions, and show that combining LODE coefficients in a way analogous to what is done with atom-centered density correlations provides a systematic way to express many-body LR physics. We also provide a fast and modular implementation of this framework using GTO as well as an optimized radial basis, which also includes calculations of gradients, making it possible to train, and predict, on forces. This physically interpretable yet generally applicable class of descriptors allows us to investigate the challenges inherent in the ML modeling of LR interactions, revealing the delicate balance between physical content and generality of a model. While it is beneficial to use descriptors that are designed to reflect the expected asymptotic decay of the interactions, we observe that in a realistic application doing so is neither strictly necessary nor a guarantee of success. LODE-based models can reach levels of accuracy of a small fraction of the typical binding energy for different types of asymptotic behavior but only when separately targeting chemically homogeneous sets of molecules. When considering a heterogeneous data set that contains very different kinds of intermolecular interactions, the large variability in the energy scale of the various physical terms makes it particularly difficult to achieve good relative accuracy for the weaker types of interactions. The description of LR physics, from the bare interactions themselves to more complex processes, such as nonlocal charge transfers, remains one of the key challenges to the application of ML techniques to atomistic simulations. The extension of the LODE framework we introduce here provides a flexible, physically motivated solution and a sandbox to investigate the effects of descriptor engineering and ML architecture, balancing interpretability and generality of the model.

## Data Availability

All used data sets as well as the input for the DFT calculations are available for download at 10.24435/materialscloud:23-99. Generalized LODE descriptors can be computed using the *rascaline* package, available at https://github.com/luthaf/rascaline. Additional source codes for constructing the monomial basis in *rascaline* as well as the code to train the linear and the neural networks are available on zenodo: 10.5281/zenodo.8399545.

## Supporting Information Available

Pedagogic derivation of the connection between generalized LODE features and the multipole expansion, further details on the different models and data sets, and the dimer data set used for the benchmarks(PDF)
Archive of all data sets containing structures, cell information, energies and forces (ZIP)
Transparent Peer Review report available (PDF)
