# Supplementary material for: Physics-Inspired Equivariant Descriptors of Nonbonded Interactions
Source: J Phys Chem Lett. 2023 Oct 20;14(43):9612–8. doi: 10.1021/acs.jpclett.3c02375 (PMC10626632; doi:10.1021/acs.jpclett.3c02375)
Supplement: Supplementary file 1 — jz3c02375_si_001.pdf [file jz3c02375_si_001.pdf]

Physics-Inspired Equivariant Descriptors of Nonbonded Interactions
Supporting Information

Kevin K. Huguenin-Dumittan,[1,*] Philip Loche,[1,*] Ni Haoran,[1] and Michele Ceriotti[1,†]

[1] *Laboratory of Computational Science and Modeling, IMX, École Polytechnique Fédérale de Lausanne, 1015 Lausanne, Switzerland*

CONTENTS

S1 Multipole Expansion: Review and Extension S2
A Overview S2
B Multipole Expansion for Coulomb Potential S3
1 Key ingredient: Laplace Expansion of Coulomb Potential S3
2 Interior Charges S5
3 Exterior Charges S6
4 Inter-Regional Interaction Energy S7
C Extension of Multipole Expansion to General Inverse Power-Law Potential S7
1 Key ingredient: Laplace Expansion of General Power-Law Potential S7
2 Interior Charges S9
3 Exterior Charges S10
4 Inter-Regional Interaction Energy S10
D Remark On Conventions and Prefactors S11

S2 Physical Interpretation and Optimization of LODE Representation S12
A Overview of this section S12
B General Framework for Atomic Representations S12
C LODE for Coulombic Interactions S13
D Extension to Arbitrary Long-Range Descriptors S14
E Physical Interpretation and Optimizations for the Coulombic Case S14
F Physical Interpretation and Optimizations for General Exponents S16
G Building ML Models S17
H Similarities and Differences of the LODE Density with the actual Coulomb Potential S17
1 Absence of SR-LR splitting S18
2 Treatment of different chemical species S18

S3 Behavior of General Density Contribution Function S20
A Long-Range Limit S20
B Short-Range Limit S20
C Coulombic Special Case S21
D Why Gaussian Densities cannot be used in the general case S21

S4 Higher Order Descriptors and Many-Body Effects S22
A Review of Invariant Descriptors S22
B Interpretation of LODE coefficients in Terms of Many-Body Interactions S22
C Results on Many-Body Dispersion S24
1 Fitting DFT Data S24
2 Model Details S24
3 Fitting the ATM Potential S25

S5 Model Details for the Point-Charge Toy problem S27

S6 Computational details for the Dimer Dataset S27
A Reference energy calculations S27
B Empirical interaction exponents S28
C SOAP and LODE Hyperparameters S28
D Linear Models: Separate Dimer Classes S28
E Atomic Force Errors S30

* Contributed equally to this work
† michele.ceriotti@epfl.ch

F Test Errors for Different Training Cutoffs S30
G Energy Errors for Combined Linear Models Using Non-Charged Fragments S31
H Details of the Neural Network Models S32
I Relative Energy Errors for Different Subsets S33

S7 Flexibility of power-law fits S34

S8 Implementation S36

References S37

S1. MULTIPOLE EXPANSION: REVIEW AND EXTENSION

A. Overview

A key ingredient for our mathematical analysis is the multipole expansion from electrostatics. To summarize the general idea, let $\rho(\boldsymbol{r})$ for $\boldsymbol{r} \in \mathbb{R}^3$ be some density function that decays sufficiently fast as $r \to \infty$, in a sense that will be made more precise later on.

As shown in Figure S1a, we split the density up into an interior part $\rho^<$ and exterior part $\rho^>$, each containing only the density inside / outside some cutoff radius $r_{\text{cut}} > 0$, respectively. More formally, this can be written as

$$\rho^<(\boldsymbol{r}) = \begin{cases} \rho(\boldsymbol{r}) & r < r_{\text{cut}} \\ 0 & r \geq r_{\text{cut}} \end{cases}, \qquad \rho^>(\boldsymbol{r}) = \begin{cases} 0 & r < r_{\text{cut}} \\ \rho(\boldsymbol{r}) & r \geq r_{\text{cut}}, \end{cases} \tag{S1}$$

where $r = \|\boldsymbol{r}\|$ and $r' = \|\boldsymbol{r}'\|$. For the rest of this document, we shall denote by $\boldsymbol{r}$ ($\boldsymbol{r}'$) a point inside (outside) the cutoff, meaning that we will always have $r < r_{\text{cut}}$ ($r' > r_{\text{cut}}$). In particular, $r < r'$ will always be true.

We begin by providing a general overview of the multipole expansion: Now that we have divided space into two regions (interior and exterior), the multipole expansion is a technique that can be used to study the potential generated by charges in one region evaluated in the other region. To be more specific, we will derive expressions for the following three quantities:

1. The "famous version": The "electrostatic" potential $V^<$ generated by the interior charges, evaluated at a point $\boldsymbol{r}'$ in the exterior region as shown in Figure S1b and explicitly given by

$$V^<(\boldsymbol{r}') = \int_0^{r_{\text{cut}}} \mathrm{d}^3\boldsymbol{r} \, \frac{\rho^<(\boldsymbol{r})}{\|\boldsymbol{r} - \boldsymbol{r}'\|^p}, \tag{S2}$$

   where the integral runs over the entire interior region with $r < r_{\text{cut}}$.

2. The opposite of the above, i.e. the potential $V^>$ generated by the exterior charges, evaluated at a point $\boldsymbol{r}$ in the interior region as shown in Figure S1c and explicitly given by

$$V^>(\boldsymbol{r}) = \int_{r_{\text{cut}}}^{\infty} \mathrm{d}^3\boldsymbol{r}' \, \frac{\rho^>(\boldsymbol{r}')}{\|\boldsymbol{r} - \boldsymbol{r}'\|^p}, \tag{S3}$$

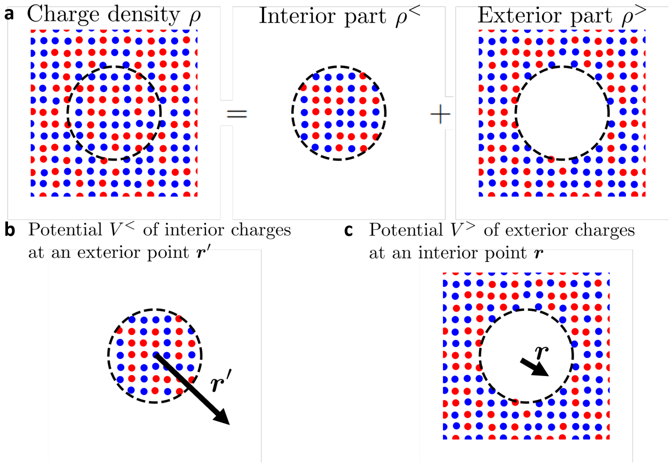

Figure S1. Visual representation of the various versions of the multipole expansion: (a) shows the splitting of a charge density into an interior and exterior part. (b) shows the "famous version" of the multipole expansion discussed in point 1. (c) shows the converse, in which the charges are outside and the potential is evaluated in the interior region.

where the integral runs over the entire exterior region with $r' > r_{\mathrm{cut}}$.

3. The "electrostatic" interaction energy between the two regions: it consists of interaction terms of the form $\frac{q_i q_j}{\|\boldsymbol{r}_i - \boldsymbol{r}'_j\|^p}$, where particle i is from the interior and j from the exterior region. In other words, it includes the inter-regional interactions but not the intra-regional ones. For the continuous charge densities treated here, this can be written as

$$E_{\mathrm{int}} = \int_0^{r_{\mathrm{cut}}} \mathrm{d}^3\boldsymbol{r} \int_{r_{\mathrm{cut}}}^{\infty} \mathrm{d}^3\boldsymbol{r}' \, \frac{\rho^<(\boldsymbol{r})\rho^>(\boldsymbol{r}')}{\|\boldsymbol{r}-\boldsymbol{r}'\|^p}. \tag{S4}$$

The multipole expansion essentially provides a mathematically elegant way to express the three quantities defined in Equations (S2), (S3) and (S4).

The separation into the two regions might at first seem arbitrary, but turns out to be a key ingredient: In Equation (S2), for instance, we can observe that for the entire region of integration, we always have $r < r'$ since $\boldsymbol{r}'$ was specifically chosen to be a point in the exterior region $r' > r_{\mathrm{cut}}$. This inequality will be essential to obtain a simple mathematical expression for the end result.

The most famous version of the multipole expansion that is discussed in many graduate level textbooks on electrodynamics[1] deals with the first case, in which the charges are confined to some interior region, and is restricted to the Coulomb potential for which $p = 1$. The same derivation with tiny modifications also lead to the analogous results for the second (Equation (S3)) and third (Equation (S4)) cases. In fact, the proofs for all cases are quite simple once one knows one key intermediate result that is common to all three versions: the Laplace expansion of the Coulomb potential. A unified description of these for the Coulomb potential is discussed in section S1 B. While all of these results are already known, we provide a mostly complete derivation here to make this document as self-contained as possible, and also to better understand the differences to the more general case.

As part of this work, the multipole expansion was extended to arbitrary exponents p. The general case turns out to be more complicated than the Coulombic special case, the underlying reason being the fact that the Coulomb potential is a solution to the Laplace equation $\Delta 1/r = 0$ for $r \neq 0$. In essence, this fact eliminates many terms that need to be taken into account for general p. The analogous results for general exponents p are discussed in section S1 C.

B. Multipole Expansion for Coulomb Potential

1. *Key ingredient: Laplace Expansion of Coulomb Potential*

All variants of the multipole expansion are quite simple to derive once we understand a single key ingredient: the Laplace expansion of the Coulomb potential. In this subsection, we will first state the result and provide some intuition for why it is such an essential tool. For the more mathematically inclined readers, this is then followed by a sketch of the proof, but readers may feel free to skip this part and move on to the following subsections in which the Laplace expansion is used to derive the various versions of the multipole expansion.

Lemma S1.1 (Laplace Expansion of Coulomb Potential)**.** *Let $\boldsymbol{r}, \boldsymbol{r}' \in \mathbb{R}^3$ such that $r < r'$. Then,*

$$\frac{1}{\|\boldsymbol{r}-\boldsymbol{r}'\|} = \sum_{l,m} \frac{4\pi}{2l+1} \frac{r^l}{r'^{l+1}} Y_l^m(\hat{\boldsymbol{r}}') Y_l^m(\hat{\boldsymbol{r}}), \tag{S5}$$

where $\sum_{l,m}$ is a shorthand for $\sum_{l=0}^{\infty}\sum_{m=-l}^{l}$ and the functions Y_l^m are the real spherical harmonics.

The key idea is that we have managed to rewrite the the original expression, which depends on both $\boldsymbol{r}$ and $\boldsymbol{r}'$, into a form in which the two variables are separated, namely:

$$\frac{1}{\|\boldsymbol{r}-\boldsymbol{r}'\|} = \sum_{l,m} \frac{4\pi}{2l+1} \cdot \underbrace{\frac{1}{r'^{l+1}} Y_l^m(\hat{\boldsymbol{r}}')}_{\text{only depends on } \boldsymbol{r}'} \cdot \underbrace{r^l Y_l^m(\hat{\boldsymbol{r}}')}_{\text{only depends on } \boldsymbol{r}} . \tag{S6}$$

The radial dependencies (the factors of r^l and $1/r'^{l+1}$) also explain why we need to split the charges into an interior and exterior part when we later discuss the full multipole expansion. For two general vectors $\boldsymbol{r}_1$ and $\boldsymbol{r}_2$, the Laplace expansion of $1/\|\boldsymbol{r}_1 - \boldsymbol{r}_2\|$ would contain factors of r_{min}^l and $1/r_{\mathrm{max}}^{l+1}$, where r_{max} (r_{min}) is the larger (smaller) of the two radii r_1 and r_2. By dividing the charges into an interior and exterior region, we will always have $r < r'$ and

thus $r_{\min} = r$, $r_{\max} = r'$. Otherwise, one would need to mix factors of $\frac{r^l}{r'^{l+1}}$ and $\frac{r'^l}{r^{l+1}}$, leading to significantly more complicated final expressions.

For readers not as familiar with the *real* spherical harmonics, these are essentially the same functions as the "usual" spherical harmonics (or complex spherical harmonics) more often encountered in the mathematical literature. They are analogous to solutions of the differential equation $f'' = -f$, two commonly used forms being $f(x) = Ae^{ix} + Be^{-ix}$ or $f(x) = C\cos x + D\sin x$. The complex spherical harmonics correspond to the exponential solutions $e^{\pm ix}$, and tend to be easier to work with in proofs, while the trigonometric functions are real-valued, and hence favorable in applications in which the target function f is known to be real. For this reason and for simpler notation, we use the real form, but all results would remain essentially the same with complex spherical harmonics, the only difference being that half of the spherical harmonics would need to be replaced by their complex conjugate.

We devote the rest of this subsection to the proof of the Laplace expansion. It is not required to understand this proof to get to the multipole expansion in the next subsection.

Proof. The proof consists of three steps. The first one only requires relatively elementary algebra, while the other two will require some knowledge about special functions (Legendre polynomials and spherical harmonics). We begin by writing the left hand side explicitly in terms of r, r' as well as the angle γ between the vectors $\boldsymbol{r}$ and $\boldsymbol{r}'$.

$$\frac{1}{\|\boldsymbol{r}-\boldsymbol{r}'\|} = \frac{1}{[(\boldsymbol{r}-\boldsymbol{r}')^2]^{\frac{1}{2}}} = \frac{1}{(r^2 + r'^2 - 2rr'\cos\gamma)^{\frac{1}{2}}} = \frac{1}{r'\left[\left(\frac{r}{r'}\right)^2 + 1 - 2\left(\frac{r}{r'}\right)\cos\gamma\right]^{\frac{1}{2}}}, \tag{S7}$$

where we pulled out a factor of r' in the last step, and the angular part can be explicitly computed as $\cos\gamma = \hat{\boldsymbol{r}} \cdot \hat{\boldsymbol{r}}'$.

For the second step, we use an identity involving Legendre polynomialsP_l in terms of a generating function, namely

$$\frac{1}{(1 - 2cx + x^2)^{\frac{1}{2}}} = \sum_{l=0}^{\infty} P_l(c)x^l \tag{S8}$$

for any $x, c \in \mathbb{R}$ with $|c| \leq 1$ and $|x| < 1$. In fact, this equation can be used to define the Legendre polynomials in the first place. We can use this identity with $x = r/r'$ and $c = \cos\gamma = \hat{\boldsymbol{r}} \cdot \hat{\boldsymbol{r}}'$, which leads us to

$$\frac{1}{\|\boldsymbol{r}-\boldsymbol{r}'\|} = \frac{1}{r'} \frac{1}{\left[1 - 2\left(\frac{r}{r'}\right)\left(\hat{\boldsymbol{r}} \cdot \hat{\boldsymbol{r}}'\right) + \left(\frac{r}{r'}\right)^2\right]^{\frac{1}{2}}} \tag{S9}$$

$$= \frac{1}{r'} \sum_{l=0}^{\infty} P_l(\hat{\boldsymbol{r}} \cdot \hat{\boldsymbol{r}}') \frac{r^l}{r'^l} = \sum_{l=0}^{\infty} P_l(\hat{\boldsymbol{r}} \cdot \hat{\boldsymbol{r}}') \frac{r^l}{r'^{l+1}}. \tag{S10}$$

Note that at this point, we have managed to separate the radial dependences with respect to r and r', but not yet the angular part.

For the third and final step, we will use the spherical harmonics addition theorem, which expresses the Legendre polynomials using spherical harmonics.

$$P_l(\hat{\boldsymbol{r}} \cdot \hat{\boldsymbol{r}}') = \sum_{m=-l}^{l} \frac{4\pi}{2l+1} Y_l^m(\hat{\boldsymbol{r}}') Y_l^m(\hat{\boldsymbol{r}}), \tag{S11}$$

where $\hat{\boldsymbol{r}}$ and $\hat{\boldsymbol{r}}'$ can be any two unit vectors in $\mathbb{R}^3$. Note that this theorem is typically derived using the complex form of the spherical harmonics, in which case the first spherical harmonic is replaced by the complex conjugate. Extending this theorem to real spherical harmonics is then straight-forward using the fact that the conversion between real and complex spherical harmonics is a unitary transformation.

Using this, we finally obtain the desired result

$$\frac{1}{\|\boldsymbol{r}-\boldsymbol{r}'\|} = \sum_{l,m} \frac{4\pi}{2l+1} \frac{r^l}{r'^{l+1}} Y_l^m(\hat{\boldsymbol{r}}') Y_l^m(\hat{\boldsymbol{r}}), \tag{S12}$$

in which we have completely separated the dependence with respect to $\boldsymbol{r}$ and $\boldsymbol{r}'$. For completeness, the analogous result with complex spherical harmonics would be

$$\frac{1}{\|\boldsymbol{r}-\boldsymbol{r}'\|} = \sum_{l,m} \frac{4\pi}{2l+1} \frac{r^l}{r'^{l+1}} Y_{\mathbb{C},l}^{*m}(\hat{\boldsymbol{r}}') Y_{\mathbb{C},l}^{m}(\hat{\boldsymbol{r}}). \tag{S13}$$

□

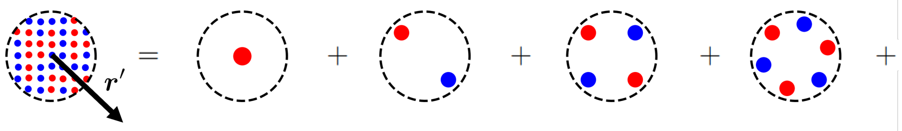

Figure S2. Visual representation of the interior multipole expansion. The potential generated by the interior charges can be decomposed into terms of increasing angular frequencies here illustrated for the first four ($l = 0, 1, 2, 3$) terms.

2. *Interior Charges*

We have now completed the most challenging part of the derivation, and are well-prepared to easily prove the multipole expansion theorems for the Coulomb potential. We begin by discussing the most well-known version of the multipole expansion, which is discussed in many textbooks on electrodynamics[1] and is illustrated in Figure S2.

Theorem S1.2. *Let $\rho^<$ be a localized charge density which is entirely contained within a cutoff radius r_{cut} and is sufficiently well-behaved to satisfy $\int_0^{r_{\text{cut}}} \mathrm{d}^3\boldsymbol{r}\, |\rho^<(\boldsymbol{r})| < \infty$. The resulting electrostatic potential $V^<(\boldsymbol{r}')$ evaluated at some point $\boldsymbol{r}'$ outside of the cutoff is given by*

$$V^<(\boldsymbol{r}') = \sum_{lm} M^<_{lm} \frac{1}{r'^{l+1}} Y_l^m(\hat{\boldsymbol{r}}'), \tag{S14}$$

with coefficients

$$M^<_{lm} = \frac{4\pi}{2l+1} \int_0^{r_{\text{cut}}} \mathrm{d}^3\boldsymbol{r}\, r^l Y_l^m(\hat{\boldsymbol{r}}) \rho^<(\boldsymbol{r}). \tag{S15}$$

The coefficients M_{lm} are called **(interior) multipole moments** and completely characterize the potential in the exterior region. The first term with $l = 0$ (and thus also $m = 0$) is called the monopole term and generates a spherically symmetric potential decaying as $1/r$. The prefactor $M^<_{00}$ for this term simply corresponds to the total charge. The second term with $l = 1$ is called the dipole term and generates a potential decaying as $1/r^2$. The three components $M^<_{1m}$ correspond to the three Cartesian components of the dipole moment vector. This term represents the first correction to the spherically symmetrical approximation. More generally, as shown in Figure S2, the multipole expansion can be thought of as a Fourier-like expansion of $\rho^<$ into angular frequency components. The term with an angular frequency l leads to a potential decaying as $1/r^{1+l}$, showing that terms with high l become less and less important as $r \to \infty$.

Proof. From the superposition principle, the electrostatic potential generated by $\rho^<$ is given by

$$V^<(\boldsymbol{r}') = \int_0^{r_{\text{cut}}} \mathrm{d}^3\boldsymbol{r}\, \frac{\rho^<(\boldsymbol{r})}{\|\boldsymbol{r} - \boldsymbol{r}'\|}. \tag{S16}$$

Since $r' > r_{\text{cut}}$, we have $r < r'$ in the entire domain of integration. This allows us to use the Laplace expansion of the Coulomb potential $\frac{1}{\|\boldsymbol{r}-\boldsymbol{r}'\|} = \sum_{l,m} \frac{4\pi}{2l+1} \frac{r^l}{r'^{l+1}} Y_l^m(\hat{\boldsymbol{r}}) Y_l^m(\hat{\boldsymbol{r}}')$ (Lemma (S5)) to write

$$V^<(\boldsymbol{r}') = \int_0^{r_{\text{cut}}} \mathrm{d}^3\boldsymbol{r}\, \rho^<(\boldsymbol{r}) \sum_{l,m} \frac{4\pi}{2l+1} \frac{r^l}{r'^{l+1}} Y_l^m(\hat{\boldsymbol{r}}) Y_l^m(\hat{\boldsymbol{r}}'). \tag{S17}$$

If $\rho^<$ is sufficiently well-behaved to satisfy $\int_0^{r_{\text{cut}}} \mathrm{d}^3\boldsymbol{r}\, |\rho^<(\boldsymbol{r})| < \infty$ (e.g. if $\rho^<$ is continuous), it is permissible to interchange the order of summation and integration using Fubini's theorem, which leads to

$$V^<(\boldsymbol{r}') = \sum_{l,m} \underbrace{\frac{4\pi}{2l+1} \int_0^{r_{\text{cut}}} \mathrm{d}^3\boldsymbol{r}\, \rho^<(\boldsymbol{r}) r^l Y_l^m(\hat{\boldsymbol{r}})}_{=:M_{lm}} \frac{1}{r'^{l+1}} Y_l^m(\hat{\boldsymbol{r}}') = \sum_{l,m} M^<_{lm} \frac{1}{r'^{l+1}} Y_l^m(\hat{\boldsymbol{r}}') \tag{S18}$$

with multipole moments

$$M^<_{lm} = \int_0^{r_{\text{cut}}} \mathrm{d}^3\boldsymbol{r}\, r^l Y_l^m(\hat{\boldsymbol{r}}) \rho^<(\boldsymbol{r}). \tag{S19}$$

This is the desired result, in which the exterior potential is written as a series in varying angular and radial dependence. □

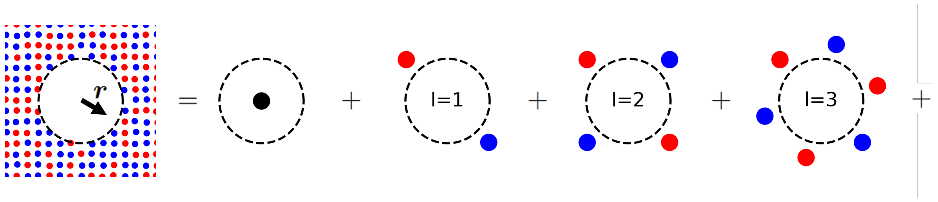

Figure S3. Visual representation of the exterior multipole expansion. The potential generated by the exterior charges can be decomposed into terms of increasing angular frequencies here illustrated for $l = 0, 1, 2, 3$ corresponding to the monopole, dipole, quadrupole and octupole terms.

3. *Exterior Charges*

We can now flip the roles between the exterior and interior regions: the charges are now outside, and we wish to compute the potential generated by these in the interior region. The result is analogous to the previous case, except that all factors of r^l and $1/r'^l$ switch roles.

Theorem S1.3. *Let $\rho^>$ be a charge density which is entirely contained outside of a cutoff radius r_{cut}, decaying sufficiently quickly such that $\int_{r_{\text{cut}}}^{\infty} \mathrm{d}^3\boldsymbol{r}' \, |\rho^>(\boldsymbol{r}')| < \infty$. The resulting electrostatic potential $V^>(\boldsymbol{r})$ evaluated at some point $\boldsymbol{r}$ inside of the cutoff is then given by*

$$V^>(\boldsymbol{r}) = \sum_{lm} M^>_{lm} r^l Y_l^m(\hat{\boldsymbol{r}}), \tag{S20}$$

with coefficients

$$M^>_{lm} = \frac{4\pi}{2l+1} \int_{r_{\text{cut}}}^{\infty} \mathrm{d}^3\boldsymbol{r}' \, \frac{1}{r'^{l+1}} Y_l^m(\hat{\boldsymbol{r}}') \rho^>(\boldsymbol{r}'). \tag{S21}$$

The coefficients $M^>_{lm}$ will be called **exterior multipole moments** in this work. As for the interior version, this can be interpreted as a decomposition of the exterior charge density $\rho^>$ into (angular) frequency components as is illustrated in Figure S3.

Proof. The proof follows along the exact same lines as the previous version, except that r and r' switch roles. From the superposition principle, the potential generated by the exterior charges is given by

$$V^>(\boldsymbol{r}) = \int_{r_{\text{cut}}}^{\infty} \mathrm{d}^3\boldsymbol{r}' \, \frac{\rho^>(\boldsymbol{r}')}{\|\boldsymbol{r} - \boldsymbol{r}'\|}, \tag{S22}$$

which is well-defined if $\rho^>$ decays sufficiently quickly as defined in the theorem. Using the Laplace expansion of the Coulomb potential (Eq. (S5)), we obtain

$$V^>(\boldsymbol{r}) = \int_{r_{\text{cut}}}^{\infty} \mathrm{d}^3\boldsymbol{r}' \, \rho^>(\boldsymbol{r}') \sum_{l,m} \frac{4\pi}{2l+1} \frac{r^l}{r'^{l+1}} Y_l^m(\hat{\boldsymbol{r}}') Y_l^m(\hat{\boldsymbol{r}}). \tag{S23}$$

If $\int_{r_{\text{cut}}}^{\infty} \mathrm{d}^3\boldsymbol{r}' \, |\rho^>(\boldsymbol{r}')| < \infty$, we can interchange the order of summation and integration using Fubini's theorem. Thus,

$$V^>(\boldsymbol{r}) = \sum_{l,m} \underbrace{\frac{4\pi}{2l+1} \int_{r_{\text{cut}}}^{\infty} \mathrm{d}^3\boldsymbol{r}' \, \frac{1}{r'^{l+1}} Y_l^m(\hat{\boldsymbol{r}}') \rho^>(\boldsymbol{r}')}_{=:M^>_{lm}} r^l Y_l^m(\hat{\boldsymbol{r}}) = \sum_{l,m} M^>_{lm} r^l Y_l^m(\hat{\boldsymbol{r}}), \tag{S24}$$

with coefficients

$$M^>_{lm} = \frac{4\pi}{2l+1} \int_{r_{\text{cut}}}^{\infty} \mathrm{d}^3\boldsymbol{r}' \, \frac{1}{r'^{l+1}} Y_l^m(\hat{\boldsymbol{r}}') \rho^>(\boldsymbol{r}'). \tag{S25}$$

This is the desired result, in which the interior potential is written as a series in angular and radial dependence. □

4. Inter-Regional Interaction Energy

We now discuss the third and final useful result that can be derived using the same mathematical methods: the "inter-regional" electrostatic interaction energy E_{int} between the charges in the two regions. More formally, let $\rho^<$ and $\rho^>$ be two charge densities that are confined to the interior / exterior region. The total electrostatic interaction energy E_{int} between the interior and exterior charges (not including the interactions between charges in the same region) is given by

$$E_{\text{int}} = \int_0^{r_{\text{cut}}} \mathrm{d}^3\boldsymbol{r} \int_{r_{\text{cut}}}^{\infty} \mathrm{d}^3\boldsymbol{r}' \, \frac{\rho^<(\boldsymbol{r})\rho^>(\boldsymbol{r}')}{\|\boldsymbol{r}-\boldsymbol{r}'\|}. \tag{S26}$$

Obtaining a multipolar expression for this interaction energy will be useful to obtain a physical interpretation of ML models that are built on top of the LODE features.

Theorem S1.4. *Let $\rho^<$ and $\rho^>$ be two charge densities for the interior and exterior regions, which satisfy $\int |\rho| < \infty$ on their respective domains. The electrostatic interaction energy E_{int} between the two regions is then*

$$E_{\text{int}} = \sum_{lm} \frac{2l+1}{4\pi} M_{lm}^> M_{lm}^<, \tag{S27}$$

where $M_{lm}^>$ and $M_{lm}^<$ are the exterior and interior multipole moments defined in the two previous section S1 B 3 and section S1 B 2, respectively.

Proof. The proof should be straight-forward to anyone who has followed the proofs of the two previous versions of the multipole expansion. We start from

$$E_{\text{int}} = \int_0^{r_{\text{cut}}} \mathrm{d}^3\boldsymbol{r} \int_{r_{\text{cut}}}^{\infty} \mathrm{d}^3\boldsymbol{r}' \, \frac{\rho^<(\boldsymbol{r})\rho^>(\boldsymbol{r}')}{\|\boldsymbol{r}-\boldsymbol{r}'\|}. \tag{S28}$$

and rewrite $1/\|\boldsymbol{r}-\boldsymbol{r}'\|$ using the Laplace expansion of the Coulomb potential (Eq. (S5)). Then, after interchanging the order of the summation over l, m and the two integrals (requiring the same two conditions as before to satisfy absolute integrability), all factors containing $\boldsymbol{r}$ and $\boldsymbol{r}'$ are grouped together, leading to the desired result. □

C. Extension of Multipole Expansion to General Inverse Power-Law Potential

We now extend this analysis to exponents other than $p = 1$, i.e. $p \in \mathbb{R}_+$. Just as before, we wish to derive three versions of the multipole expansion, corresponding to the three equations (S2) for interior charges, (S3) for exterior charges and (S4) for the interaction energy between the two regions. The key ingredient will be the generalization of the Laplace expansion of the Coulomb potential, discussed in section S1 B 1, to the general case. All variants of the multipole expansion will then follow in a straight-forward manner.

Note that different generalizations of the multipole expansion for general exponents have been proposed in the past, either based on the use of Cartesian coordinates or modified spherical harmonics. These methods, however, are less suitable for many ML applications in which we require a more systematic expansion in terms of the (usual) spherical harmonics due to symmetry reasons.

1. Key ingredient: Laplace Expansion of General Power-Law Potential

What made the Laplace expansion of the Coulomb potential so powerful was the fact that the expression $1/\|\boldsymbol{r}-\boldsymbol{r}'\|$ could be written in a way that provided a separation of the $\boldsymbol{r}$ and $\boldsymbol{r}'$ dependence. Thus, in order to generalize the mutipole expansion to arbitrary $1/r^p$ potentials, we need to derive an analogous expansion of $1/\|\boldsymbol{r}-\boldsymbol{r}'\|^p$. We begin by stating this generalized result and provide some intuition. As before, readers can feel free to skip the proof and continue to the next subsection, in which this lemma is used to prove the actual multipole expansion theorems.

Lemma S1.5. *Let $\boldsymbol{r}, \boldsymbol{r}' \in \mathbb{R}^3$ such that $r < r'$ and $p \in \mathbb{R}_+$. Then,*

$$\frac{1}{\|\boldsymbol{r}-\boldsymbol{r}'\|^p} = \sum_{nlm} \frac{4\pi}{2l+1} A_{n,l}^p \frac{r^{l+2n}}{r'^{p+l+2n}} Y_l^m(\boldsymbol{r}') Y_l^m(\boldsymbol{r}), \tag{S29}$$

where $\sum_{nlm}$ is a shorthand for $\sum_{n=0}^{\infty} \sum_{l=0}^{\infty} \sum_{m=-l}^{l}$ and $A_{n,l}^p$ are suitable coefficients.

Compared to the special case of $p = 1$ presented in Equation S5, we observe that the general version is more involved: it requires a sum over an additional index n, rather than just l, m. This is what makes all potentials with $p \neq 1$ qualitatively different from the Coulomb potential. As briefly mentioned before, $p = 1$ is special because applying the (three-dimensional) Laplace operator to $1/r$, we obtain $\Delta 1/r = 0$ for $r \neq 0$. It is, from a deeper point of view, this fact that eliminates all coefficients other than those where $n = 0$ in the Coulombic case.

Proof. The proof is similar to the one of Lemma (S5), except that there is one extra step that leads to the more complicated multipole expansion also requiring a sum over n. The first step again uses simple algebra to rewrite the denominator as

$$\frac{1}{\|\boldsymbol{r}-\boldsymbol{r}'\|^p} = \frac{1}{(r^2 + r'^2 - 2rr'\cos\gamma)^{\frac{p}{2}}} = \frac{1}{r'^p\left[\left(\frac{r}{r'}\right)^2 + 1 - 2\left(\frac{r}{r'}\right)\cos\gamma\right]^{\frac{p}{2}}}, \tag{S30}$$

where $\cos\gamma = \hat{\boldsymbol{r}} \cdot \hat{\boldsymbol{r}}'$ is the angle between $\boldsymbol{r}$ and $\boldsymbol{r}'$.

For the next step, we will use a set of special functions called Gegenbauer polynomials (also known as ultraspherical polynomials), which are a generalization of Legendre polynomials. These obey the relation

$$\frac{1}{(1-2cx+x^2)^{p/2}} = \sum_{q=0}^{\infty} C_q^{p/2}(c)x^q. \tag{S31}$$

for any $x, c \in \mathbb{R}$ with $|c| \leq 1$ and $|x| < 1$ (alternatively, one could use this as a way to define the Gegenbauer polynomials in terms of a generating function). Note that for $p = 1$, the Gegenbauer polynomials $C_q^{1/2}$ reduce to the Legendre polynomials. We can use this identity with $x = r/r'$ and $c = \cos\gamma = \hat{\boldsymbol{r}} \cdot \hat{\boldsymbol{r}}'$, which leads us to

$$\frac{1}{\|\boldsymbol{r}-\boldsymbol{r}'\|^p} = \sum_{q=0}^{\infty} C_q^{p/2}(\hat{\boldsymbol{r}} \cdot \hat{\boldsymbol{r}}')\frac{r'^q}{r^{p+q}}. \tag{S32}$$

Note that we have called the index of summation q rather than l, for reasons that we will now explain.

In our application, we require a form of the multipole expansion that is written in terms of the spherical harmoncis functions Y_l^m. Thus, we need to find a way to express $C_q^{p/2}$ in terms of spherical harmonics. While no direct such relation is known to the authors, we can exploit the fact that Gegenbauer polynomials share many similarities to the Legendre polynomials: for all $p > 0$, $C_q^{p/2}$ is a polynomial of degree q. If q is even (odd), then the function $C_q^{p/2}$ is even (odd) as well. Thus, using the analogous properties of the Legendre polynomials, we can see that there must exist some coefficients $\tilde{A}_{q,l}^p$ that allow us to express $C_q^{p/2}$ as a linear combination of Legendre polynomials P_l

$$C_q^{p/2} = {\sum_l}' \tilde{A}_{q,l}^p P_l, \tag{S33}$$

where the prime in the summation indicates that l runs over $l = q, q-2, q-4, \ldots$ up to 0 or 1 depending on whether q is even or odd due to the parity of both P_l and C_q. We thus obtain

$$\frac{1}{\|\boldsymbol{r}-\boldsymbol{r}'\|^p} = \sum_{q=0}^{\infty}{\sum_l}' \tilde{A}_{q,l}^p P_l(\hat{\boldsymbol{r}} \cdot \hat{\boldsymbol{r}}')\frac{r'^q}{r^{p+q}}. \tag{S34}$$

We can already observe a key difference to the electrostatic special case: For the Coulomb potential, the term decaying as $1/r^{1+l}$ only had an angular dependence of the form P_l, while for general p, the term decaying as $1/r^{p+q}$ requires multiple angular terms P_l with $l = q, q-2, \ldots$ and so on. Equivalently, and more conveniently for our application, we can group together all terms that belong to the same value of l and say that the term with an angular dependence of P_l has radial dependence $1/r^{p+q}$ for $q = l, l+2, l+4, \ldots$. This can be reparametrized as $q = l + 2n$ for $n = 0, 1, 2, \ldots$. Thus, we obtain the general relation

$$\sum_{q=0}^{\infty}\sum_{l=q,q-2,\ldots} f(q,l) = \sum_{l=0}^{\infty}\sum_{n=0}^{\infty} f(l+2n, l) \tag{S35}$$

which can be applied to our case to yield

$$\frac{1}{\|\boldsymbol{r}-\boldsymbol{r}'\|^p} = \sum_{l=0}^{\infty}\sum_{n=0}^{\infty} \tilde{A}_{l+2n,l}^p P_l(\hat{\boldsymbol{r}} \cdot \hat{\boldsymbol{r}}')\frac{r'^{l+n}}{r^{p+l+n}} =: \sum_{l=0}^{\infty}\sum_{n=0}^{\infty} A_{n,l}^p P_l(\hat{\boldsymbol{r}} \cdot \hat{\boldsymbol{r}}')\frac{r'^{l+n}}{r^{p+l+n}}, \tag{S36}$$

where for convenience, we have re-indexed the coefficients $\tilde{A}^p_{l+2n,l}$ as $A^p_{n,l}$.

Finally, we can again use the spherical harmonics addition theorem

$$P_l(\hat{\boldsymbol{r}} \cdot \hat{\boldsymbol{r}}') = \sum_{m=-l}^{l} \frac{4\pi}{2l+1} Y_l^m(\hat{\boldsymbol{r}}') Y_l^m(\hat{\boldsymbol{r}}) \tag{S37}$$

to obtain the final expression

$$\frac{1}{\|\boldsymbol{r} - \boldsymbol{r}'\|^p} = \sum_{nlm} \frac{4\pi}{2l+1} A^p_{n,l} \frac{r^{l+2n}}{r'^{p+l+2n}} Y_l^m(\boldsymbol{r}') Y_l^m(\boldsymbol{r}), \tag{S38}$$

which concludes the proof. □

2. Interior Charges

We can now discuss the three versions of the multipole expansion for general exponent p. As for the Coulombic case, the most challenging part of the derivation is the Laplace expansion in Equation (S29). If we accept this result, deriving the multipole expansion is just a simple extra step consisting of regrouping terms.

Theorem S1.6. *Let $\rho^<$ be a localized charge density which is entirely contained within a cutoff radius r_{cut} and is sufficiently well-behaved to satisfy $\int_0^{r_{\text{cut}}} \mathrm{d}^3\boldsymbol{r}\, |\rho^<(\boldsymbol{r})| < \infty$. The resulting "electrostatic" (inverse power-law) potential $V^<(\boldsymbol{r}')$ with exponent p evaluated at some point $\boldsymbol{r}'$ outside of the cutoff is given by*

$$V^<(\boldsymbol{r}') = \sum_{nlm} M^<_{nlm} \frac{1}{r'^{p+l+2n}} Y_l^m(\hat{\boldsymbol{r}}'), \tag{S39}$$

with coefficients

$$M^<_{nlm} = \frac{4\pi}{2l+1} A^p_{n,l} \int_0^{r_{\text{cut}}} \mathrm{d}^3\boldsymbol{r}\, r^{l+2n} Y_l^m(\hat{\boldsymbol{r}}) \rho^<(\boldsymbol{r}), \tag{S40}$$

and where $\sum_{nlm}$ is a shorthand for $\sum_{n=0}^{\infty} \sum_{l=0}^{\infty} \sum_{m=-l}^{l}$.

Proof. From the superposition principle, the potential generated by the interior charges is given by

$$V^<(\boldsymbol{r}') = \int_0^{r_{\text{cut}}} \mathrm{d}^3\boldsymbol{r}\, \frac{\rho^<(\boldsymbol{r})}{\|\boldsymbol{r} - \boldsymbol{r}'\|^p}. \tag{S41}$$

Rewriting $1/\|\boldsymbol{r} - \boldsymbol{r}'\|^p$ using the generalized Laplace expansion (Equation (S29)), we obtain

$$V^<(\boldsymbol{r}') = \int_0^{r_{\text{cut}}} \mathrm{d}^3\boldsymbol{r}\, \rho^<(\boldsymbol{r}) \sum_{nlm} \frac{4\pi}{2l+1} A^p_{n,l} \frac{r^{l+2n}}{r'^{p+l+2n}} Y_l^m(\boldsymbol{r}) Y_l^m(\boldsymbol{r}'). \tag{S42}$$

If $\int_0^{r_{\text{cut}}} \mathrm{d}^3\boldsymbol{r}\, |\rho^<(\boldsymbol{r})| < \infty$, it is permissible to interchange the oder of summation and integration. Thus,

$$V^<(\boldsymbol{r}') = \sum_{nlm} \underbrace{\left[\frac{4\pi A^p_{n,l}}{2l+1} \int_0^{r_{\text{cut}}} \mathrm{d}^3\boldsymbol{r}\, \rho^<(\boldsymbol{r}) r^{l+2n} Y_l^m(\boldsymbol{r}) \right]}_{=: M^<_{nlm}} \frac{1}{r'^{p+l+2n}} Y_l^m(\boldsymbol{r}') = \sum_{nlm} M^<_{nlm} \frac{1}{r'^{p+l+2n}} Y_l^m(\boldsymbol{r}') \tag{S43}$$

with

$$M^<_{nlm} = \frac{4\pi A^p_{n,l}}{2l+1} \int_0^{r_{\text{cut}}} \mathrm{d}^3\boldsymbol{r}\, \rho^<(\boldsymbol{r}) r^{l+2n} Y_l^m(\boldsymbol{r}). \tag{S44}$$

Thus, using the identical steps as for the Coulombic case, we obtain the desired result. □

3. Exterior Charges

Just as for the Coulomb potential, we can now flip the roles between the exterior and interior regions: the charges are now outside, and we wish to compute the potential generated by these in the interior region. The result is analogous to the previous case, except that all factors of r^{l+2n} and $1/r'^{p+l+2n}$ switch roles.

Theorem S1.7. *Let $\rho^>$ be a charge density which is entirely contained outside of a cutoff radius r_{cut}, decaying sufficiently quickly such that $\int_{r_{\text{cut}}}^{\infty} \mathrm{d}^3\boldsymbol{r}' \, |\rho^>(\boldsymbol{r}')| < \infty$. The resulting "electrostatic" (inverse power-law) potential $V^>(\boldsymbol{r})$ with exponent p evaluated at some point $\boldsymbol{r}$ inside of the cutoff is given by*

$$V^>(\boldsymbol{r}) = \sum_{nlm} M^>_{nlm} r^{l+2n} Y_l^m(\hat{\boldsymbol{r}}), \tag{S45}$$

with coefficients

$$M^>_{nlm} = \frac{4\pi}{2l+1} A^p_{n,l} \int_{r_{\text{cut}}}^{\infty} \mathrm{d}^3\boldsymbol{r}' \, \frac{1}{r'^{p+l+2n}} Y_l^m(\hat{\boldsymbol{r}}') \rho^>(\boldsymbol{r}'), \tag{S46}$$

and where $\sum_{nlm}$ is a shorthand for $\sum_{n=0}^{\infty} \sum_{l=0}^{\infty} \sum_{m=-l}^{l}$.

Proof. The proof is completely analogous to the various versions presented so far, consisting of the steps:

1. Start from the expression $V^>(\boldsymbol{r}) = \int_{r_{\text{cut}}}^{\infty} \mathrm{d}^3\boldsymbol{r}' \, \frac{\rho^>(\boldsymbol{r}')}{\|\boldsymbol{r}-\boldsymbol{r}'\|^p}$ (Equation (S3))
2. Use the Laplace expansion (Equation (S29)) for $1/\|\boldsymbol{r}-\boldsymbol{r}'\|^p$
3. Interchange the order of summation (over n, l, m) and integration using Fubini's theorem
4. Regroup factors that depend on $\boldsymbol{r}'$ to get the coefficients $M^>_{nlm}$

□

4. Inter-Regional Interaction Energy

Finally, we discuss how to compute the "inter-regional" interaction energy E_{int} between the charges in the two regions, namely

$$E_{\text{int}} = \int_0^{r_{\text{cut}}} \mathrm{d}^3\boldsymbol{r} \int_{r_{\text{cut}}}^{\infty} \mathrm{d}^3\boldsymbol{r}' \, \frac{\rho^<(\boldsymbol{r})\rho^>(\boldsymbol{r}')}{\|\boldsymbol{r}-\boldsymbol{r}'\|^p}. \tag{S47}$$

As for the Coulomb potential, obtaining a multipolar expression for this interaction energy will be useful to obtain a physical interpretation of ML models that are built on top of the LODE features.

Theorem S1.8. *Let $\rho^<$ and $\rho^>$ be two charge densities for the interior and exterior regions, which satisfy $\int |\rho| < \infty$ on their respective domains. The general power-law interaction energy E_{int} between the two regions is then*

$$E_{\text{int}} = \sum_{nlm} \frac{2l+1}{4\pi A^p_{n,l}} M^>_{nlm} M^<_{nlm}, \tag{S48}$$

where $M^>_{nlm}$ and $M^<_{nlm}$ are the exterior and interior multipole moments defined in the two previous subsections S1 C 3 and S1 C 2, respectively.

Proof. The proof is completely analogous to the various versions presented so far, consisting of the steps:

1. Start from the expression $E_{\text{int}} = \int_0^{r_{\text{cut}}} \mathrm{d}^3\boldsymbol{r} \int_{r_{\text{cut}}}^{\infty} \mathrm{d}^3\boldsymbol{r}' \, \frac{\rho^<(\boldsymbol{r})\rho^>(\boldsymbol{r}')}{\|\boldsymbol{r}-\boldsymbol{r}'\|^p}$ (Equation (S4))
2. Use the Laplace expansion (Equation (S29)) for $1/\|\boldsymbol{r}-\boldsymbol{r}'\|^p$
3. Interchange the order of summation (over n, l, m) and integration using Fubini's theorem
4. Group all factors that depend on $\boldsymbol{r}'$ together to get the coefficients $M^>_{nlm}$, and those that depend on $\boldsymbol{r}$ together to get the coefficients $M^<_{nlm}$

□

D. Remark On Conventions and Prefactors

The definitions of the various multipolar quantities in this work might differ from those found in some of the literature. We briefly summarize some conventions.

- We write all results using the real versions of the spherical harmonics, rather than the complex form that is more commonly used in mathematical textbooks. Since all relevant target functions in our case are ultimately real-valued, this leads to more efficient storage in software implementations, and leads to slightly simpler notation.

  All results do however also apply if we use complex spherical harmonics instead, since the transformation between real and complex spherical harmonics is unitary, with a single modification. In most places, the spherical harmonics appear in pairs. One of the two spherical harmonics (which one does not matter) will then need to get replaced by the complex conjugate. For the Laplace expansion of the Coulomb potential, this is explicitly shown at the end of the proof of Lemma S5. All further results can be translated in a straight-forward manner.

- We refer to the coefficients $M_{lm}^{<}$ (or $M_{nlm}^{<}$) that characterize **interior** charges as **interior** multipole moments. On the other hand, some works would call this the **exterior** multipole expansion, since we evaluate the potential at a point $\boldsymbol{r}'$ in the exterior region (and vice versa if the charges are outside). We decided to use this terminology because the focus in this work is on the atoms (charges), rather than where the potential is evaluated.

- Our definition of multipole moments, e.g. those in sections S1 B 2 and S1 B 3 contain the prefactors of $\frac{4\pi}{2l+1}$. It would, however, also be possible not to include this factor in the coefficient itself, and to keep it in the final summation. This clearly does not affect any of the conclusions. The current convention was chosen to maximize the mathematical analogies to the density based descriptors which are discussed in more detail in the next section within this Supporting Information.

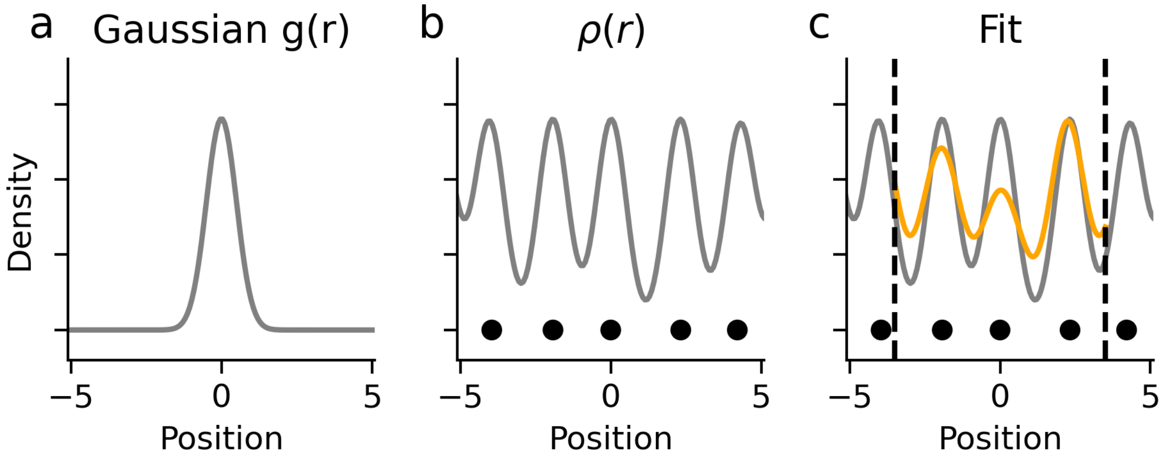

Figure S4. Visualization of the steps involved to generate short-range descriptors, including the popular SOAP descriptor. a: Density contribution function showing the density for a single atom. b: Density of the structure as a whole, obtained by considering superpositions of the function in part a for each atom. c: Fitting the target density within some cutoff requires the specification of some fitting parameters, which contain the information on the atomic positions.

S2. PHYSICAL INTERPRETATION AND OPTIMIZATION OF LODE REPRESENTATION

A. Overview of this section

In this section, we provide a complete and mostly self contained description of the mathematical theory behind the LODE descriptors that are used throughout the main text. We begin by reviewing a general mathematical framework for the construction of atomic descriptors that unifies LODE and many SR features (e.g. SOAP) that is described in Ref. 2. We believe that the way we present the concepts here will help to better see the connection between the different representations as well as the multipole expansion discussed in the previous section in this Supporting Information.

B. General Framework for Atomic Representations

We begin by providing a quick summary of a general framework to construct atomic representations, using the better-established SR descriptors as an example. We are given an atomic structure consisting of N atoms (with or without periodic boundary conditions) indexed as $i = 1, 2, \dots, N$, whose position vectors are denoted by $\boldsymbol{r}_i$ and chemical species by $a_i \in \{\mathrm{H}, \mathrm{He}, \mathrm{Li}, \dots\}$.

We start by defining a density contribution function g, a popular choice being a Gaussian function

$$g(\boldsymbol{r}) \propto \exp(-\boldsymbol{r}^2/2\sigma^2), \tag{S49}$$

where σ is a parameter controlling the width, as shown in Figure S4a. Different normalizations for the Gaussian are possible, but the specific choice is irrelevant to this discussion.

This density contribution function g is then used to define a density function ρ over the entire structure, $\rho(\boldsymbol{r}) = \sum_j g(\boldsymbol{r} - \boldsymbol{r}_j)$ shown in Figure S4b. This function ρ is not directly related to any physical quantity (e.g. the electron density), but contains the information about the location of all atoms in the structure. For instance, we could imagine that we "hide" all atoms from Figure S4b, and only show the density function. Since the locations of the atoms correspond to peaks in ρ, we can reconstruct the atomic positions (up to some precision that is determined by the smearing σ).

To describe the local neighborhood around an atom i rather than the entire structure, we use the atom centered density $\rho_i(\boldsymbol{r}) = \rho(\boldsymbol{r} + \boldsymbol{r}_i)$, truncated at some cutoff radius $r = \|\boldsymbol{r}\| \leq r_{\mathrm{cut}}$, i.e. only the portion of ρ around $\boldsymbol{r}_i$ (the cutoff is shown with dashed lines in Figure S4c).

For computational applications, it is necessary to describe the neighborhood of atom i using a discrete set of coefficients, rather than an abstract function ρ_i. Thus, we choose a set of orthonormal basis functions B_{nlm}, which for symmetry reasons are typically of the form $B_{nlm}(\boldsymbol{r}) = R_{nl}(r)Y_l^m(\hat{\boldsymbol{r}})$, where $R_{nl}(r)$ are some radial basis functions

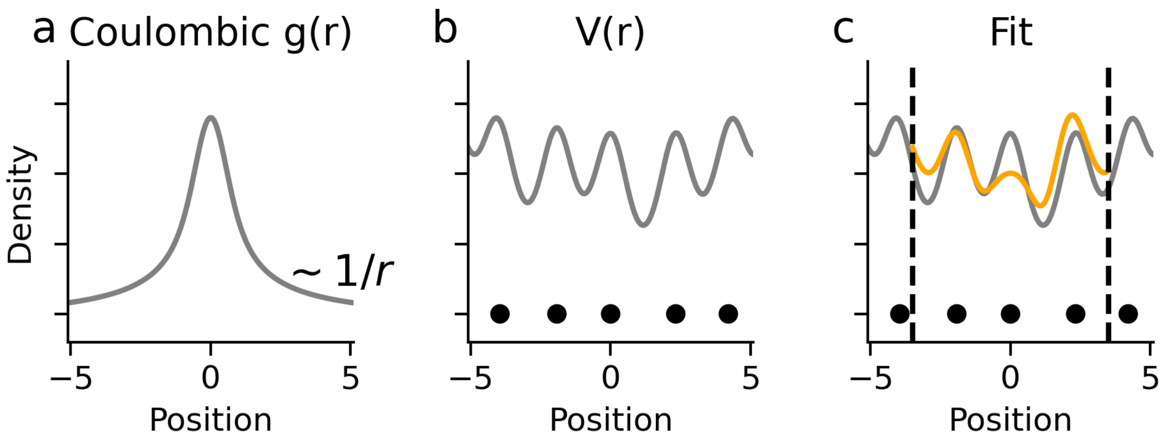

Figure S5. Visualization of the steps involved to generate the LODE descriptors. a: Density contribution function showing the density for a single atom. As opposed to a Gaussian, the functions now decay as $1/r$. b: Density of the structure as a whole, obtained by considering superpositions of the function in part a for each atom. c: Fitting the target density within some cutoff requires the specification of some fitting parameters, which contain the information on the atomic positions, now also including atoms outside of the cutoff due to the slow decay.

only depending on the distance r, and $Y_l^m(\hat{\boldsymbol{r}})$ are the real spherical harmonics describing the angular dependence. Any continuous function including ρ_i can then be written as the linear combination of the basis functions

$$\rho_i(\boldsymbol{r}) = \sum_{nlm} \rho_{i,nlm} B_{nlm}(\boldsymbol{r}) = \sum_{nlm} \rho_{i,nlm} R_{nl}(r) Y_l^m(\hat{\boldsymbol{r}}), \tag{S50}$$

with coefficients $\rho_{i,nlm}$, provided that the radial basis functions R_{nl} satisfy certain completeness relations. Furthermore, we typically choose the basis functions to be orthonormal, which allows us to easily compute the coefficients $\rho_{i,nlm}$ using the inner product of the basis function with the target density ρ_i,

$$\rho_{i,nlm} = \int_0^{r_{\text{cut}}} \mathrm{d}^3\boldsymbol{r}\, R_{nl}(r) Y_l^m(\hat{\boldsymbol{r}}) \rho_i(\boldsymbol{r}). \tag{S51}$$

The coefficients $\rho_{i,nlm}$ contain the information about the atomic positions around atom i, and can thus be used in ML applications to predict atomic properties. In reality, in most applications, the coefficients are not used directly: for instance, if the target property is invariant under rotations (e.g. the energy), rotationally invariant combinations of these coefficients are used. This can also be generalized to arbitrary transformation behaviors with respect to rotations, leading to equivariant models.

The SR nature of the descriptor arises from the use of a quickly decaying function for the density contribution function g, common choices being the aforementioned Gaussian function[3,4] and the infinitely sharp limit of delta distributions $g(\boldsymbol{r}) \to \delta(\boldsymbol{r})$.[5,6] Since only neighbor atoms j in the immediate neighborhood of the center atom i contribute to the density within the cutoff radius, the SR nature of g enables efficient predictions of materials properties scaling linearly with the number of atoms. More details on SR descriptors are discussed in Ref. 2.

C. LODE for Coulombic Interactions

While this framework was originally developed for SR descriptors, it permits an elegant extension to arbitrary LR interactions. By changing the density contributions g to a slowly decaying function, we obtain a new density function $\rho(\boldsymbol{r})$ for which even the local neighborhood around an atom i contains more information about far-away atoms.

Motivated by the fact that the most important LR interaction is the electrostatic one, the original work on LODE[7] used a density contribution of the form

$$g_{\text{Coul}}(\boldsymbol{r}) = \frac{\operatorname{erf}\left(r/\sqrt{2}\sigma\right)}{r}, \tag{S52}$$

where $\operatorname{erf}(x)$ is the error function. This corresponds to the Coulomb potential of a Gaussian charge density, commonly used for Ewald summation.[1] As for the Coulomb potential, this function asymptotically behaves as $1/r$ for $r \to \infty$,

while the singularity at $r = 0$ of the bare Coulomb potential has been smeared out as can be seen in Figure S5a. To differentiate this Coulombic density from the SR ones, as well as to emphasize the mathematical analogy to the Coulomb potential of the structure, we write $V(\boldsymbol{r}) = \sum_j g_{\text{Coul}}(\boldsymbol{r} - \boldsymbol{r}_j)$ rather than $\rho(\boldsymbol{r})$ for the total density which is shown in Figure S5b.

Just as for SR densities, for each center atom i, an atomic descriptor is constructed by first defining the shifted (atom centered) version of the density, $V_i(\boldsymbol{r}) = V(\boldsymbol{r} + \boldsymbol{r}_i)$ defined up to $r = \|\boldsymbol{r}\| \leq r_{\text{cut}}$. To get a discrete set of coefficients, this density is projected onto the basis functions B_{nlm}, leading to coefficients

$$V_{i,nlm} = \int_0^{r_{\text{cut}}} \mathrm{d}^3\boldsymbol{r}\, R_{nl}(r) Y_l^m(\hat{\boldsymbol{r}}) V_i(\boldsymbol{r}). \tag{S53}$$

The key idea is that due to the slow decay, even atoms outside the cutoff will provide significant contributions to the interior density (Figure S5c). The only conceptual difference to the SR version is thus a different choice of the density contribution function g, which allows us to easily reuse all ML methods that work for SR models and combine them with the LR modifictions. This is the key idea of the LODE framework.

Note that for systems with periodic boundary conditions, the sum $V(\boldsymbol{r}) = \sum_j g(\boldsymbol{r} - \boldsymbol{r}_j)$ is understood to include all periodic images. While this doesn't significantly affect SR descriptors using density contributions g that decay quickly (e.g. Gaussians or delta distributions), the LR descriptors require a more proper treatment of the infinitely many periodic images using an Ewald-like Fourier space formulation, which is discussed in section S8. Thus, while conceptually close to SR densities, actual implementations to compute the coefficients $V_{i,nlm}$ will be quite different from their SR counterparts.

D. Extension to Arbitrary Long-Range Descriptors

Given the diversity of $1/r^p$ interactions, $1/r^6$ being the most important apart from the Coulomb potential from a physical point of view, it is natural to extend LODE to other exponents p. This can be achieved by finding a function g behaving as $1/r^p$ for $r \to \infty$, while being monotonous in r and differentiable in the entire space. We use

$$g_p(\boldsymbol{r}) = g_p(r) = \frac{1}{\Gamma\left(\frac{p}{2}\right)} \frac{\gamma\left(\frac{p}{2}, \frac{r^2}{2\sigma^2}\right)}{r^p}, \tag{S54}$$

where $\Gamma(x)$ is the Gamma function, and $\gamma(a, x)$ is the lower incomplete Gamma function. Up to different scaling, this is a functional form that was first introduced in[8] and extended in.[9,10]

It should be noted that Equation (S54) is not simply the effective $1/r^p$ potential generated by a Gaussian charge density, which would naively be the first candidate function. In fact, such an approach would not work for exponents $p \geq 3$ due to the strong singularity at the origin. Nevertheless, Equation (S54) reduces to Equation (S52) for $p = 1$. Some mathematical details of this density contribution function, including the fact that it satisfies all desired properties, are discussed in section S3.

Summarizing the general recipe to generate the descriptors discussed so far, the methods are defined by the choices of (1) the density contributions $g(\boldsymbol{r})$ and (2) the radial basis functions $R_{nl}(r)$ on which the resulting density is projected. Different SR models as well as the transition to various LR models is primarily characterized by the choice of g. In the next two subsections, we show how a suitable choice of R_{nl} leads to a clean physical interpretation of descriptors as well as more efficient implementations. While all $1/r^p$ potentials share many properties with the Coulombic special case of $p = 1$, the general case is more complicated and is discussed separately after building a good intuition based on the Coulomb potential first.

E. Physical Interpretation and Optimizations for the Coulombic Case

So far, we have provided a mathematical recipe to compute both SR and LODE features, including the generalization to arbitrary exponents p. We will now show how a more detailed mathematical analysis can provide both a more physical interpretation of these coefficients, as well as a mathematical optimization method for more efficient implementations.

In terms of mathematical prerequisites, the rest of this section will make use of two key properties of real spherical harmonics (which can essentially be thought of as the real and imaginary parts of the usual complex spherical

harmonics). The first one is the mutual orthogonality, explicitly given by

$$\int_{S^2} \mathrm{d}S\, Y_l^m Y_{l'}^{m'} = \int_0^{2\pi} \mathrm{d}\phi \int_0^{\pi} \mathrm{d}\theta\, \sin\theta Y_l^m(\theta,\phi) Y_{l'}^{m'}(\theta,\phi) = \delta_{l,l'}\delta_{m,m'}, \tag{S55}$$

where S^2 is the unit sphere characterized by the two spherical angles θ and ϕ. The second property is that any continuous (more generally, L^2) function f in the angles (θ,ϕ) (on the sphere S^2) can be expressed as a Fourier series

$$f = \sum_{l,m} f_{l,m} Y_l^m, \tag{S56}$$

with coefficients

$$f_{l,m} = \int_{S^2} \mathrm{d}S\, Y_l^m f. \tag{S57}$$

Summarizing the two properties more compactly, we can say that the spherical harmonics form an orthonormal (Schauder) basis for the (L^2)-space of functions on the unit sphere.

The last key ingredient for our analysis is the multipole expansion from electrostatics, which was already used in Ref. 11. That work in fact claimed that

> ...neither the atom density ρ_i nor the associated potential V_i correspond to physical quantities ...

and that only certain combinations of $\rho_{i,nlm}$ and $V_{i,nlm}$

> ...entail(s) formal similarities with physics-based electrostatic models.

We will show that a more careful analysis using the multipole expansion does in fact provide a direct physical interpretation to both sets of coefficients $\rho_{i,nlm}$ and $V_{i,nlm}$ in terms of electrostatic quantities. This connection also provides us with an explicit recipe for faster algorithmic implementations. A self contained review of the multipole expansion for the Coulomb potential, as well as an extension to arbitrary exponents p that was performed as part of this work, are discussed in section S1 C. For readers only interested to the applications to atomistic descriptors, we briefly summarize the main results.

Let ρ_i be the atomic density around atom i, but now without the restriction due to a cutoff radius. As shown in Figure S1a, we split it up into an interior part $\rho_i^<$ and exterior part $\rho_i^>$, each only containing the density inside / outside the cutoff radius r_{cut}, respectively, such that $\rho_i = \rho_i^< + \rho_i^>$. For the rest of this section, we shall denote by $\boldsymbol{r}$ ($\boldsymbol{r}'$) a point inside (outside) the cutoff, i.e. $r = \|\boldsymbol{r}\| < r_{\mathrm{cut}}$ ($r' = \|\boldsymbol{r}'\| > r_{\mathrm{cut}}$).

The classical version of the multipole expansion is discussed in many textbooks on electrodynamics,[1] and is illustrated in Figure S1b: The electric potential $V_i^<(\boldsymbol{r}')$ generated by a localized (interior) charge density $\rho_i^<$ evaluated at some point $\boldsymbol{r}'$ in the exterior region can be written as

$$V_i^<(\boldsymbol{r}') = \sum_{lm} M_{i,lm}^< \frac{1}{r'^{l+1}} Y_l^m(\hat{\boldsymbol{r}}'), \tag{S58}$$

with coefficients

$$M_{i,lm}^< = \int_0^{r_{\mathrm{cut}}} \mathrm{d}^3\boldsymbol{r}\, r^l Y_l^m(\hat{\boldsymbol{r}}) \rho_i^<(\boldsymbol{r}), \tag{S59}$$

which are called (interior) multipole moments and completely characterize the potential in the exterior region. Intuitively, this corresponds to a Fourier-like expansion of the interior density into terms of higher and higher frequencies.

Comparing this with Equation S51, we can see that the SR features $\rho_{i,nlm}$ can be made to exactly match the interior multipole moment $M_{i,lm}^<$ of the density $\rho_i^<$ within the cutoff radius around atom i, if we choose the radial basis function $R_{nl}(r) = r^l$ for some n, which provides us with SR features that have an exact electrostatic interpretation.

Flipping the roles around, it is possible to compute the elctrostatic potential $V_i^>(\boldsymbol{r})$ generated by the exterior charges $\rho_i^>$, evaluated at an interior point $\boldsymbol{r}$, as is shown in Figure S1c. Similar to the previous case, the potential is completely characterized by coefficients $M_{i,lm}^>$, the exterior multipole moments, such that

$$V_i^>(\boldsymbol{r}) = \sum_{lm} M_{i,lm}^> r^l Y_l^m(\hat{\boldsymbol{r}}), \tag{S60}$$

$$M_{i,lm}^> = \int_{r_{\mathrm{cut}}}^{\infty} \mathrm{d}^3\boldsymbol{r}'\, \frac{1}{r'^{l+1}} Y_l^m(\hat{\boldsymbol{r}}') \rho_i^>(\boldsymbol{r}'). \tag{S61}$$

As for the interior version, this can be interpreted as a decomposition of the exterior charge density $\rho^>$ into (angular) frequency components.

To show the analogy to the LODE coefficients, we now project this density $V_i^>(\boldsymbol{r})$ onto basis functions $B_{nlm}(\boldsymbol{r}) = R_{nl}(r)Y_l^m(\hat{\boldsymbol{r}})$. From the orthonormality of the spherical harmonics, we get

$$V_{i,nlm}^> = \int_0^{r_\mathrm{cut}} \mathrm{d}^3\boldsymbol{r}\, R_{nl}(r)Y_l^m(\hat{\boldsymbol{r}})V_i^>(\boldsymbol{r}) = M_{i,lm}^> \int_0^{r_\mathrm{cut}} \mathrm{d}r\, r^{2+l}R_{nl}(r). \tag{S62}$$

Thus, we can see that the projection of $V_i^>$ allows us to recover the exterior multipole moment $M_{i,lm}^>$ up to a prefactor that only depends on the choice of radial basis. Choosing the same radial function $R_{nl}(r) = R_l(r) = r^l$, we obtain

$$V_{i,lm} = C_l M_{i,lm}^>, \quad C_l = \frac{r_\mathrm{cut}^{3+2l}}{3+2l}. \tag{S63}$$

Thus, if it was possible to extract from the full potential $V_i(\boldsymbol{r})$ the part $V_i^>(\boldsymbol{r})$ that corresponds to the contribution of exterior charges, a comparison with Equation (S53) shows that the LODE coefficients $V_{i,nlm}$ correspond precisely to the exterior multipole moment $M_{i,lm}^>$, for the charge density $\rho_i^>$ defined relative to the central atom i. Eliminating the interior part $\rho_i^<$ is simple in principle, since this can be implemented using the same methods as for SR densities.

There are two important messages that we can learn from this analysis. Firstly, beyond the formal analogy originally proposed in Ref. 11 for the special case of linear models, we can see that the coefficients $\rho_{i,nlm}$ and a suitable modification of $V_{i,nlm}$ correspond *exactly* to the interior and exterior multipole moments of the "density" $\rho_i(\boldsymbol{r})$ centered around atom i, if we interpret it as a charge density (see remarks in section S2 H of the Supporting Information regarding the sign conventions and the more subtle treatment of different chemical species) separated into two regions by the cutoff radius r_cut around center atom i.

Secondly, we can connect this to a significant speedup of codes implementing LODE. While typical approaches for ML models use a large number of radial basis functions, $n = 0, 1, 2, \ldots, n_\mathrm{max} - 1$ with n_max on the order of $4 \sim 12$, this analysis shows us that the entire LR information about atoms outside of the cutoff can be captured by using a single radial basis function, namely r^l. This results in a reduction of the computational cost as well as memory of LODE by a factor of n_max, which can be close to an order of magnitude.

F. Physical Interpretation and Optimizations for General Exponents

We now extend this analysis to exponents other than $p = 1$. Firstly, we need to start by deriving a generalization of the multipole expansion that is suitable for our use case. While different such forms have been proposed, either based on the use of Cartesian coordinates or modified spherical harmonics, these methods are less suitable for many ML applications that require a more systematic expansion in terms of spherical harmonics.

Stating the main result first, we have shown in section section S1 that the potential $V_i^>(\boldsymbol{r})$ at a point $\boldsymbol{r}$ in the interior region generated by an exterior charge density $\rho_i^>(\boldsymbol{r}')$ can be written as

$$V_i(\boldsymbol{r}) = \int_{r_\mathrm{cut}}^{\infty} \mathrm{d}^3\boldsymbol{r}'\, \frac{\rho_i(\boldsymbol{r}')}{\|\boldsymbol{r}-\boldsymbol{r}'\|^p} = \sum_{nlm} M_{i,nlm}^> r^{l+2n} Y_l^m(\hat{\boldsymbol{r}}), \tag{S64}$$

where $M_{i,nlm}^>$ are suitable generalizations of the multipole moments that completely characterize the potential within the cutoff sphere. Similarly to the Coulombic special case, we can extract these coefficients and use them as inputs to a ML model using a suitable radial basis.

This form shows one key difference: The sum now also runs over $n = 0, 1, 2$, etc., indicating a more complicated radial dependence. The underlying mathematical reason is the fact that the Coulomb potential $g(\boldsymbol{r}) = 1/r$ is a solution to the Laplace equation $\Delta g = 0$ for $\boldsymbol{r} \neq 0$, while this is no longer true for any other $p \neq 1$.

Just as for the Coulombic case, we can extract the coefficients $M_{i,nlm}^>$ by choosing an appropriate set of radial basis functions $R_{nl}(r)$, the extra complication being the fact that for each angular channel l, the radial dependence is no longer a simple function with a single coefficient.

Nevertheless, keeping in mind that the term with $M_{i,nlm}^>$ in the generalized multipole expansion discussed in section S1 C 3 contains information about the density decaying as $1/r^{p+l+2n}$, we propose the following method to pick the best exponents. First, one chooses a maximal decay exponent $1/r^{p_\mathrm{max}}$, with $p_\mathrm{max} = p + l_\mathrm{max}$. Then, we use all coefficients $M_{i,nlm}^>$ for which $p + l + 2n < p_\mathrm{max}$ as input to our ML model. This allows us to interpret the cutoff as a truncation in decay speed of the potentials. For a given level of desired accuracy p_max, this approach requires about half as many coefficients compared to a more naive implementation in which a range of values for l and n are specified independently.

G. Building ML Models

A connection between LODE and the multipole expansion was already mentioned in previous work[11] for the special case of linear models, the key difference of this work being that (1) beyond being a mathematical analogy, we see that the LODE coefficients are *precisely* suitably defined multipole moments characterizing the interior and exterior atoms, and (2) the mathematical equivalence arises directly at the level of the coefficients, and is not restricted to the use of linear models.

While our discussion so far has focused entirely on the descriptors, and not on the model, it is important to discuss the final step that will lead to the actual target property.

Just as for SR descriptors, we know that the power spectrum coefficients of the form

$$I[\rho_i \otimes V_i]_{nn'l} = \sum_{m=-l}^{l} \rho_{i,nlm} V_{i,n'lm} \tag{S65}$$

for any n, n', l are rotationally invariant, and can thus be used as inputs to predict invariant target properties like the energy or charge of a system. While many other invariants can be constructed in a similar manner, these specific ones allow for a particularly simple interpretation.

To find an analogous quantity in electrostatics, let ρ once again be an arbitrary charge density, divided up into an interior and exterior part relative to some origin. The electrostatic interaction energy E_{int} between the interior charges $\rho^<$ and exterior charges $\rho^>$ (but not the interactions among themselves) is then given by

$$E_{\mathrm{int}} = \int_0^{r_{\mathrm{cut}}} \mathrm{d}^3\boldsymbol{r} \int_{r_{\mathrm{cut}}}^{\infty} \mathrm{d}^3\boldsymbol{r}' \, \frac{\rho^<(\boldsymbol{r})\rho^>(\boldsymbol{r}')}{\|\boldsymbol{r}-\boldsymbol{r}'\|}. \tag{S66}$$

Using the multipole expansion, it can be shown that

$$E_{\mathrm{int}} = \sum_{lm} \frac{2l+1}{4\pi} M^>_{lm} M^<_{lm}. \tag{S67}$$

In other words, the interaction energy is completely specified by knowing both the interior and exterior multipole moments of the charge distribution.

Beyond the Coulomb potential, the proof of the general multipole expansion for arbitrary exponents p can be used to also obtain the analogous result for general p, namely

$$E_{\mathrm{int}} = \int_0^{r_{\mathrm{cut}}} \mathrm{d}^3\boldsymbol{r} \int_{r_{\mathrm{cut}}}^{\infty} \mathrm{d}^3\boldsymbol{r}'\,' \frac{\rho^<(\boldsymbol{r})\rho^>(\boldsymbol{r}')}{\|\boldsymbol{r}-\boldsymbol{r}'\|^p} = \sum_{nlm} \frac{2l+1}{4\pi A^p_{l,n}} M^<_{nlm} M^>_{nlm}. \tag{S68}$$

This can be compared with a linear model on the invariants in Eq. (S65) to predict the "atomic energy" E_i,

$$E_i = \sum_{nn'l} c_{nn'l} I[\rho_i \otimes V_i]_{nn'l} = \sum_{nn'lm} c_{nn'l} \rho_{i,nlm} V_{i,n'lm}. \tag{S69}$$

Remembering that $\rho_{i,nlm}$ can be chosen to essentially correspond to $M^<_{nlm}$, while $V_{i,nlm}$ is $M^>_{nlm}$ for the charge density centered around atom i, we can see that we essentially recover the same mathematical form as the actual interaction energy between the two regions discussed in section S1, with the extra flexibility of choosing the coefficients $c_{nn'l}$ in a non-uniform way. This is essentially the result presented in[11] rewritten in our notation, but with a cleaner separation of the connection between the multipole moments and the final model.

Clearly, the usage of the LODE coefficients $V_{i,nlm}$ is not restricted to linear models, and thanks to the mathematical closeness to SR approaches, any software architecture that works with SR descriptors should be capable of seemlessly integrating LODE to incorporate far-field contributions. Furthermore, the coefficients $V_{i,nlm}$ also posses the full equivariance discussed in,[12] and can thus be used to learn target properties of arbitrary tensorial character including dipole moments or electron densities.

H. Similarities and Differences of the LODE Density with the actual Coulomb Potential

The density function V that is used throughout the LODE framework is, by construction, closely related to the Coulomb potential. This is also why, to help the intuition, we informally refer to it as "the potential (field)" throughout this document. There are, however, two subtle differences to the actual Coulomb potential that one might compute, e.g. for MD simulations.

Summarized in a few sentences each, these are:

- Traditional methods for electrostatics including the Ewald based ones or PPPM split the potential into a SR and LR part, and treat the LR part with Fourier transforms for efficient evaluation. In this picture, LODE basically only is using the LR part.
- In MD, each atom is assigned a charge q_i (often one charge per chemical species that depends on the system), and the potential is the sum of the bare $1/r$ potential weighted by the charges. In LODE, each chemical species is assigned a separate density field, and are thus treated completely independently.

In the following two subsections, each of these points is discussed further.

1. *Absence of SR-LR splitting*

For the Coulombic case, it is possible to interpret the density contribution function $g_{\mathrm{Coul}}(r)$ as the effective potential generated by a Gaussian charge density. In fact, many textbooks introduce methods such as Ewald summation by starting from a Gaussian charge density, and later introducing g as the resulting potential. This way of introducing the function $g(r)$ certainly has the advantage of being visually and physically pleasing, since the resulting method has a clear interpretation. In these applications, the total Coulomb potential is split into a SR and LR part, namely

$$\frac{1}{r} = g(r) + h(r) = \frac{\mathrm{erf}(r/\sqrt{2}\sigma)}{r} + \frac{\mathrm{erfc}(r/\sqrt{2}\sigma)}{r}, \tag{S70}$$

where the second term $h(r)$ is the SR part containing the complementary error function $\mathrm{erfc}(x) = 1 - \mathrm{erf}(x)$.

Splitting the Coulomb potential in this way has the advantage that the first term is a weakly varying function containing the LR tail, and thus is easy to calculate in reciprocal space using Fourier transforms, while the second term is a quickly decaying SR function containing the singularity as $r \to 0$, and can thus be efficiently evaluated in real space. In traditional molecular dynamics simulations, explicitly including the SR part is important to get accurate energy and force predictions. LODE, on the other hand, only includes the LR part of this decomposition, the idea being that the SR part is typically already well covered by established approaches.

2. *Treatment of different chemical species*

A second way in which the LODE density function V differs from the usual electrostatic potential is the treatment of different atomic species. At the simplest level, in traditional MD simulations, each atomic species is assigned a charge q (e.g. for NaCl, one could set $q_{\mathrm{Na}} = +1$ and $q_{\mathrm{Cl}} = -1$ in units of the elementary charge). The "physical" electrostatic potential $V_{\mathrm{Coul}}(\boldsymbol{r})$ then formally corresponds to the expression

$$V_{\mathrm{Coul}}(\boldsymbol{r}) = \sum_j q_j g_{\mathrm{bare}}(\|\boldsymbol{r} - \boldsymbol{r}_j\|), \tag{S71}$$

where the sum runs over all atoms in the structure (in general, including periodic images), and $g_{\mathrm{bare}}(r) = 1/r$ is the (bare) Coulomb potential. In other words, regardless of the number of chemical species present in the system, we only obtain one potential function $V_{\mathrm{Coul}}(\boldsymbol{r})$.

For LODE, on the other hand, each species is treated separately. Taking NaCl as an example, we would then obtain two separate density functions, namely

$$V_{\mathrm{Na}}(\boldsymbol{r}) = \sum_{j\in\mathrm{Na}} g(\|\boldsymbol{r} - \boldsymbol{r}_j\|) \tag{S72}$$

$$V_{\mathrm{Cl}}(\boldsymbol{r}) = \sum_{j\in\mathrm{Cl}} g(\|\boldsymbol{r} - \boldsymbol{r}_j\|), \tag{S73}$$

where the sum in the first (second) line only runs over all Na (Cl) atoms, respectively, including their periodic images. Note also that the charges q_j in the summation has been removed. Furthermore, the bare potential $g_{\mathrm{bare}}(r) = 1/r$ has been replaced by the appropriate smeared version $g(r)$. We shall not touch upon this point since it was already discussed in the previous subsection. These two functions could be called the "Na-potential" and "Cl-potential", respectively. The "physical" Coulomb potential, if we ignore the distinction between g_{bare} and g, then is simply the linear combination

$$V_{\mathrm{Coul}}(\boldsymbol{r}) = q_{\mathrm{Na}} V_{\mathrm{Na}}(\boldsymbol{r}) + q_{\mathrm{Cl}} V_{\mathrm{Cl}}(\boldsymbol{r}). \tag{S74}$$

From the point of view of ML models, it means that the information about the "physical" Coulomb potential is contained in the LODE potentials, but the latter still have more flexibility, and describe the location of the atoms separately for each species.

Clearly, in a data set containing atoms of N_a different species, there would be one such potential V_a for each species, where a runs over all possible such elements.

All the steps discussed in the main text are then separately applied to each of these potentials. Thus, after picking a center atom i for which we wish to compute the atom-centered features, we compute the potential function for species a centered around atom i, as $V_{ia}(\boldsymbol{r}) = V_a(\boldsymbol{r} - \boldsymbol{r}_i)$ and project onto a basis, leading to a set of coefficients $V_{ia,nlm}$ defined by

$$V_{ia} = \sum_{nlm} V_{ia,nlm} B_{nlm}. \tag{S75}$$

One further subtlety has to be considered for periodic systems: for the traditional Ewald summation based methods, it can be shown that (for exponents $p < 3$, which includes the important Coulomb case) the well-definedness of the potential function requires that the unit cell as a whole is charge neutral. This condition is relatively easy to satisfy for systems in which the charges q_j are chosen manually. On the other hand, as is apparent from equations (S72) and (S73), all terms in the summation are assigned the same charge. Physically, this problem can be solved by adding a homogeneous background charge of opposite sign to the system, as is also done for, e.g. the Jellium model.

S3. BEHAVIOR OF GENERAL DENSITY CONTRIBUTION FUNCTION

We show that the density contribution function

$$g(\boldsymbol{r}) = g(r) = \frac{1}{\Gamma\left(\frac{p}{2}\right)} \frac{\gamma\left(\frac{p}{2}, \frac{r^2}{2\sigma^2}\right)}{r^p}, \tag{S76}$$

has the desired mathematical properties, namely that it behaves as $1/r^p$ as $r \to \infty$ while removing the singularity at the origin.

The Gamma function is defined as

$$\Gamma(a) = \int_0^\infty t^{a-1} e^{-t} \mathrm{d}t, \tag{S77}$$

while the lower incomplete Gamma function is defined similarly, but the integration range restricted to $[0, x]$:

$$\gamma(a, x) = \int_0^x t^{a-1} e^{-t} \mathrm{d}t. \tag{S78}$$

A. Long-Range Limit

In the limit as $r \to \infty$, we want $g(r)$ to asymptotically behave as $1/r^p$. From the definition of the lower incomplete Gamma function, we can immediately obtain

$$\gamma(a, x) = \int_0^x t^{a-1} e^{-t} \mathrm{d}t \overset{x \to \infty}{\longrightarrow} \int_0^\infty t^{a-1} e^{-t} \mathrm{d}t = \Gamma(a) \tag{S79}$$

Thus, we can indeed see that

$$g(r) \sim \frac{1}{r^p} \quad \text{as} \quad r \to \infty, \tag{S80}$$

where the equivalence relation $f(x) \sim g(x)$ for $x \to x_0$ means that f and g asymptotically behave in the same way, formally defined as $\lim_{x \to x_0} \frac{f(x)}{g(x)} = 1$.

B. Short-Range Limit

To convince ourselves that the singularity at $r = 0$ is properly taken care of, we can use the asymptotic expansion

$$\gamma(a, x) = \frac{x^a}{a} + O(x^{a+1}) \tag{S81}$$

as $x \to 0$, which can directly be obtained from Taylor expanding the exponential function, and using Fubini's theorem to interchange the order of summation and integration (since the integrand is absolutely integrable):

$$\gamma(a, x) = \int_0^x t^{a-1} \sum_{n=0}^\infty (-1)^n \frac{t^n}{n!} \mathrm{d}t = \sum_{n=0}^\infty \frac{(-1)^n}{n!} \int_0^x t^{a-1} t^n \mathrm{d}t = \sum_{n=0}^\infty \frac{(-1)^n}{n!} \frac{x^{a+n}}{a+n} \tag{S82}$$

$$= \frac{1}{a} x^a + O(x^{a+1}). \tag{S83}$$

Using this, we can see that the limit at $r \to 0$ is indeed a well defined nonzero value, namely

$$\lim_{r \to 0} g(r) = \lim_{r \to 0} \frac{1}{\Gamma\left(\frac{p}{2}\right)} \frac{\gamma\left(\frac{p}{2}, \frac{r^2}{2\sigma^2}\right)}{r^p} = \lim_{r \to 0} \frac{1}{\Gamma\left(\frac{p}{2}\right)} \cdot \frac{1}{\frac{p}{2}} \left(\frac{r^2}{2\sigma^2}\right)^{\frac{p}{2}} \cdot \frac{1}{r^p} = \frac{1}{\Gamma(\frac{p+2}{2}) \, (2\sigma^2)^{\frac{p}{2}}}, \tag{S84}$$

with corrections that are quadratic in r for small nonzero $r > 0$.

C. Coulombic Special Case

We begin by showing that for the special case of $p = 1$, we recover the Coulomb potential of a Gaussian density

$$g_{\text{Coulomb}}(r) = \frac{\text{erf}(r/\sqrt{2}\sigma)}{r}. \tag{S85}$$

Comparing with the general expression, it suffices to show that

$$\text{erf}(r/\sqrt{2}\sigma) = \frac{\gamma\left(\frac{1}{2}, \frac{r^2}{2\sigma^2}\right)}{\Gamma(1/2)} = \frac{\gamma\left(\frac{1}{2}, \frac{r^2}{2\sigma^2}\right)}{\sqrt{\pi}} \tag{S86}$$

since the factor of $1/r$ is common to both. This equality follows directly from the definition of the error function

$$\text{erf} z = \frac{2}{\sqrt{\pi}} \int_0^z e^{-t^2} \text{d}t \overset{u=t^2}{=} \frac{1}{\sqrt{\pi}} \int_0^{z^2} u^{-\frac{1}{2}} e^{-u} \text{d}u = \frac{\gamma\left(\frac{1}{2}, z^2\right)}{\sqrt{\pi}}. \tag{S87}$$

Plugging in $z^2 = \frac{r^2}{2\sigma^2}$, we obtain the desired result.

D. Why Gaussian Densities cannot be used in the general case

As was shown in this section, the density contribution functions $g(r)$ have a well-defined limit as $r \to 0$. Naively, just as for the Coulombic special case, one could hope to achieve this by using the effective potential generated by a Gaussian charge density. This, however, would not work for $p \geq 3$.

The qualitative way of seeing this is to work in spherical coordinates and study the asymptotic scaling of the potential as $r \to 0$. While the bare $1/r$ potential has a singularity at the origin, the Coulomb potential generated by a Gaussian charge (or any continuous charge density for that matter) is still finite at the origin due to the extra factor of r^2 arising from the Jacobian in spherical coordinates. More generally, $1/r^p \cdot r^2 = r^{2-p}$ can be integrated without leading to singularities as long as $p < 3$. Thus, if we wish to have a set of densities $g_p(r)$ that also varies smoothly with respect to p, it is impossible to use Gaussian densities.

Instead, if we consider the effective $1/r^p$ potential generated by a spherically symmetric density $\rho(\boldsymbol{r})$, the potential at a point $\boldsymbol{r}$ is then given by

$$\phi(\boldsymbol{r}) = \int_{\mathbb{R}^3} \frac{\rho(\boldsymbol{r}')}{\|\boldsymbol{r} - \boldsymbol{r}'\|^p} = \frac{2\pi}{(2-p)r} \int_0^\infty \text{d}r' r' \rho(r') \left[|r - r'|^{-p+2} - (r + r')^{-p+2}\right] \tag{S88}$$

In our case, $\phi(\boldsymbol{r})$ would correspond to the density contribution function $g(\boldsymbol{r})$. Using the specific form in Equation (S76), one can invert the relation and compute what the effective charge density (or smearing) $\rho(r)$ would need to be to get this specific effective density.

S4. HIGHER ORDER DESCRIPTORS AND MANY-BODY EFFECTS

The main text has mostly focused on LODE and LR interactions as a 2-body interaction. In this section we discuss how the same coefficients can also be combined to produce higher order descriptors containing general n-body information.

A. Review of Invariant Descriptors

We start with a brief review of the analogous results for SR descriptors, and then translate the results to LODE. Since the early successful ML models for the energy of a system,[3,13] much effort has been dedicated to searching for ways to describe the local environment of an atom in a way that is invariant under translations and rotations. Well-known geometrical descriptors having these properties are the interatomic distances (2-body), angles (3-body) and dihedrals (4-body). Thus, many of the earlier ML models explicitly used these as inputs. In parallel, driven by the popularity of the SOAP descriptor,[4] a range of descriptors based on the density ρ as introduced in section S2 have been developed and systematically extended. In particular, if we start from the coefficients $\rho_{i,nlm}$ of the density, also written as $\langle nlm|\rho_i\rangle$ in other works and explained in more detail in section S2 (see Eq. (S50)), one can generate a systematic hierarchy of rotationally invariant descriptors by taking polynomial combinations of these coefficients, which is the key idea underlying the implementation of models like ACE[6] and NICE.[14] In particular, it was shown that the invariant coefficients linear, quadratic and cubic in ρ contain 2-, 3- and 4-body information, meaning that these contain the information on the distances, angles and dihedrals introduced above. Furthermore, beyond these geometrically interpretable quantities, one can more generally combine ν such coefficients to build a descriptor that contains $\nu+1$-body information. The intuition here is that the center atom i is given, and each copy of ρ adds one neighbor atom, which is why it is $\nu+1$ rather than just ν. More explicitly, the invariant descriptors at the linear ($\nu=1$, physically corresponding to 2-body) and quadratic ($\nu=2$, physically corresponding to 3-body) level are given by

$$I[\rho]_n = \rho_{i,n00} \tag{S89}$$

$$I[\rho\otimes\rho]_{nn'l} = \sum_{m=-l}^{l} \rho_{nlm}\rho_{n'lm} \tag{S90}$$

When used as descriptors in a linear regression model, the $\nu=1$ invariants can be used to parametrize 2-body functions, and more generally, a linear model built on invariants of degree ν lead to an effective $\nu+1$-body function. By combining multiple such coefficients, one obtains a systematic way to approximate many-body potentials.

Moving to LODE, we recall that the coefficients $V_{i,nlm}$ are defined completely analogously to $\rho_{i,nlm}$, the only difference being the choice of density contribution $g(\boldsymbol{r})$. In particular, their transformation behavior under rotations of the system is exactly the same, arising from the properties of the spherical harmonics. Thus,

$$I[V]_n = V_{i,n00} \tag{S91}$$

$$I[V\otimes V]_{nn'l} = \sum_{m=-l}^{l} V_{nlm}V_{n'lm} \tag{S92}$$

are the invariant under rotation, and are the simplest invariants one can build corresponding to $\nu=12$ and $\nu=2$. More generally, invariant expressions for $\nu\geq 3$ can be constructed as well using Clebsh-Gordan coefficients, similarly to the density bispectrum[15] and higher-body order invariants used in SR models. Beyond invariants, arbitrary-order equivariant descriptors, involving combinations of short and long-range terms, can be obtained with an iterative procedure as discussed in Refs. 16,17.

B. Interpretation of LODE coefficients in Terms of Many-Body Interactions

We will now show how a linear model built on the descriptors $I^{(\nu)}$ for some degree ν can be interpreted as a parametric family of $\nu+1$-body functions. For simplicity, we will write the results in this section by using the energy E as a target property, but the argument would equally apply to any other target property that is invariant under rotations. We will assume a general interaction exponent p throughout this section. Furthermore, we will assume that only the atoms (density) outside of the cutoff radius generates the potential, since it is the exterior part that we wish to capture with LODE (the interior part can be subtracted using more conventional methods).

We first discuss the two cases $\nu = 1$ and $\nu = 2$ separately, and follow with the general calculation. Starting with $\nu = 1$, it was shown in section S1 that the information content in the coefficients $V^{>}_{i,nlm}$ essentially corresponds to the exterior multipole moment $M^{>}_{i,nlm}$ defined as

$$M^{>}_{i,nlm} \propto \int_{r_{\mathrm{cut}}}^{\infty} \mathrm{d}^3\boldsymbol{r}' \, \frac{\rho(\boldsymbol{r}')}{r'^{p+l+2n}} \tag{S93}$$

and in particular for $l = m = 0$:

$$M^{>}_{i,n00} \propto \int_{r_{\mathrm{cut}}}^{\infty} \mathrm{d}^3\boldsymbol{r}' \, \frac{\rho(\boldsymbol{r}')}{r'^{p+2n}} \tag{S94}$$

This, however, is just the total $1/r^{p+2n}$ potential generated by the exterior charges. This can be made even more explicit by assuming that the density $\rho = \sum_j q_j \delta(\boldsymbol{r} - \boldsymbol{r}_{ij})$ is made up of point particles containing charges q_j, in which case we get

$$M^{>}_{i,n00} \propto \sum_j \frac{q_j}{r'^{p+2n}_{ij}}, \tag{S95}$$

where we have more explicitly written $r_{ij} = \|\boldsymbol{r}_j - \boldsymbol{r}_i\|$ to emphasize that we are working in a coordinate system centered around some atom i. Thus, if we use a linear model built on these features to predict, say, the "atomic energy" ϵ_i of a system, this corresponds to a fit of the form

$$\epsilon_{i,\mathrm{pred}} = \sum_n \tilde{w}_n M^{>}_{i,n00} = \sum_j q_j \sum_n w_n \frac{1}{r'^{p+2n}_{ij}}, \tag{S96}$$

where we have absorbed some proportionality constants into the definition of w_n. The last form makes it particularly clear that we are effectively fitting the total energy using a linear combination of $1/r^p, 1/r^{p+2}, 1/r^{p+4}, \ldots$ up to some maximal exponent. We thus obtain a model that predicts energies as a linear combination of 2-body terms.

Moving on to the quadratic case, $\nu = 2$, we consider a linear model built on

$$I[V \otimes V]_{nn'l} = \sum_{m=-l}^{l} V_{nlm} V_{n'lm}. \tag{S97}$$

Writing only the basis functions that can be combined with the appropriate fitting coefficients, this is equivalent to an expansion in terms of the products of the multipole moments

$$\sum_m M^{>}_{i,nlm} M^{>}_{i,n'lm} = \sum_m \int_{r_{\mathrm{cut}}}^{\infty} \mathrm{d}^3\boldsymbol{r}' \int_{r_{\mathrm{cut}}}^{\infty} \mathrm{d}^3\boldsymbol{r}'' \, \frac{\rho(\boldsymbol{r}')}{r'^{p+l+2n}} \frac{\rho(\boldsymbol{r}'')}{r"^{p+l+2n'}} Y_l^m(\boldsymbol{r}') Y_l^m(\boldsymbol{r}'') \tag{S98}$$

Using the spherical harmonics addition theorem,

$$\frac{4\pi}{2l+1} \sum_m Y_l^m(\hat{\boldsymbol{r}}_1) Y_l^m(\hat{\boldsymbol{r}}_2) = P_l(\hat{\boldsymbol{r}}_1 \cdot \hat{\boldsymbol{r}}_2), \tag{S99}$$

we can rewrite the above as

$$\sum_m M^{>}_{i,nlm} M^{>}_{i,n'lm} = \int_{r_{\mathrm{cut}}}^{\infty} \mathrm{d}^3\boldsymbol{r}' \int_{r_{\mathrm{cut}}}^{\infty} \mathrm{d}^3\boldsymbol{r}'' \, \frac{\rho(\boldsymbol{r}')}{r'^{p+l+2n}} \frac{\rho(\boldsymbol{r}'')}{r"^{p+l+2n'}} P_l(\hat{\boldsymbol{r}}_1 \cdot \hat{\boldsymbol{r}}_2) \tag{S100}$$

Again, taking the limit in which the density $\rho_i = \sum_j q_j \delta(\boldsymbol{r} - \boldsymbol{r}_{ij})$ is built as a sum of delta distributions, we obtain

$$\sum_m M^{>}_{i,nlm} M^{>}_{i,n'lm} = \sum_{j,k} \frac{q_j q_k}{r_{ij}^{p+l+2n} r_{ik}^{p+l+2n'}} P_l(\hat{\boldsymbol{r}}_{ij} \cdot \hat{\boldsymbol{r}}_{ik}). \tag{S101}$$

This form makes clear that the invariant component corresponding to the indices n, n', l corresponds to a 3-body interaction

$$V^{3-\mathrm{body}}_{nn'l}(r_{ij}, r_{ik}, \theta_{jk}) = \frac{1}{r_{ij}^{p+l+2n} r_{ik}^{p+l+2n'}} P_l(\cos\theta_i) \tag{S102}$$

parametrized by the two sidelengths r_{ij}, r_{ik} of the triangle formed by atoms i, j, k, as well as the angle θ_i between these sides formed at the position of atom i. The combinations of multiple coefficients p, l, n provides a family of models that can capture 3-body interactions with arbitrary decay exponents. We will examine the flexibility of the many-body LODE features in the next subsection.

For $\nu = 1, 2$, we have explicitly written out the radial and angular dependencies of the resulting potential. While the angular part becomes more complicated for higher order invariants, the general intuition remains the same. By combining ν multipole moments characterized by (p_1, n_1, l_1), (p_2, n_2, l_2), $\ldots$, (p_ν, n_ν, l_ν), one can build invariant coefficients representing an $\nu+1$-body interaction with arbitrary decay exponents with respect to neighbor distances, and complex dependencies on the relative spatial arrangements of far-field atoms.

C. Results on Many-Body Dispersion

1. Fitting DFT Data

We now show how a linear model only built on $\nu = 1$ invariants can not learn the 3-body part of many-body dispersion, while a model also using $\nu = 2$ can. We generated a dataset consisting of 100 Xenon dimers, as well as 100 Xenon trimers in which the three atoms are placed on an equilateral triangle. For both, the distance d between the atoms (the side length of the triangle for the trimers) has been varied between 3.5 and 14 Å. The energies of the molecules were computed using the identical settings as for the molecular dimers discussed in section S6 A.

We then learn these energies using a linear model including two different features: only the invariants linear in the LODE coefficients, $I[V]_n$, which describe pair interactions, as well as the quadratic invariants $I[V \otimes V]_{nn'l}$ that contain 3-body information. The models are trained on a subset of the entire dataset only containing the molecules up to the cutoff separation of 7.5Å, the results of which are shown in Figure S6. The left column (subfigures a and c) shows the results of the first model only using the two-body descriptors, while the right column (subfigures b and d) uses a model that uses both two- and three-body LODE features.

Superficially, just comparing the panels (a) and (c) showing the total energy of the dimers and trimers, the two models seem to yield similar performance. The difference becomes clear, however, once we compare the accuracy on the three-body part of the energy, shown in panels (b) and (d). We see in (b) that the two-body model cannot learn the three-body part of the energy at all, while in (d), we see that a model which includes three-body LODE features can capture the behavior well. Moreoever, looking at the scale of the energy shows that the three-body part of the energy is less than a tenth of the total energy. Thus, the apparent good performance of the two-body-only model seen in panel (a) is simply due to the fact that the dominant two-body component was captured well. Higher accuracies, however, clearly need to capture the three-body part as well, requiring the use of the higher order LR features.

2. Model Details

For the two-body features, we used a potential exponent $p = 6$ to match the leading term of dispersion interaction, with a monomial basis consisting of $n_{\max} = 6$ basis functions and a smearing parameter of $\sigma = 1$Å. For the three-body features, we used the same radial basis, $p = 4$ (such that the effective decay potential $2p = 8$ is close enough to the theoretical asymptotic value of 9, which corresponds to the total exponent in the Axilrod-Teller-Muto (ATM) potential, that is often used to describe 3-body dispersion interactions), $l_{\max} = 4$ and $n_{\max} = 6$. The learning was performed using the ridge regression class of scikit-learn with regularization parameter $\alpha = 10^{-12}$.

Since the two atoms for the dimers and three atoms for the trimers have the exactly same environment (up to rotations), these will have the same rotationally invariant descriptors $I[V]_n$ and $I[V \otimes V]_{nn'l}$ (recall that $I[V \otimes \cdots \otimes V]$ with ν factors contains $\nu+1$-body information). The model can thus be effectively written as

$$E^{(2b)}_{\text{pred}} = 2 \sum_{n=0}^{n_{\max}-1} w^{(2)}_n I[V]_n \tag{S103}$$

$$E^{(3b)}_{\text{pred}} = 3 \left(\sum_{n=0}^{n_{\max}-1} w^{(2)}_n I[V]_n + \sum_{n,l,l'} w^{(3)}_{n,n',l} I[V \otimes V]_{nn'l} \right) \tag{S104}$$

with model parameters $w^{(2)}_n$ and $w^{(3)}_{n,n',l}$, and the same loss function as in section S5.

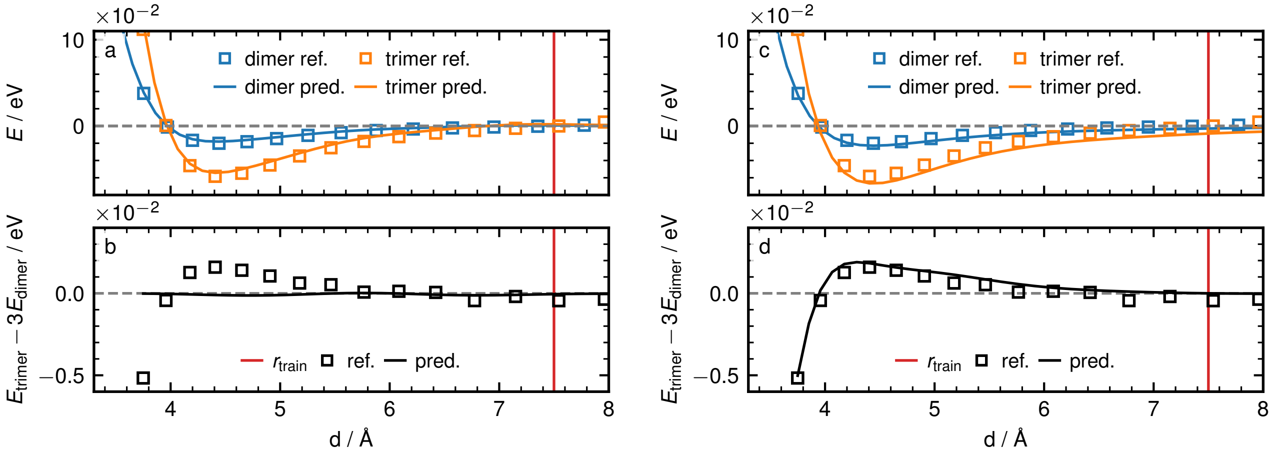

Figure S6. Fitting of three-body dispersion using LODE. The two figures on top (a,c) show the target data and predicted curves for the total energy of both the dimers and trimers as a function of the interatomic distance d. The two figures on the bottom (b,d) show the analogous result for the three-body part of the energy, defined as $E_{\mathrm{3b}}(d) = E_{\mathrm{trimer}}(d) - 3E_{\mathrm{dimer}}(d)$. For the left hand column, a linear model only using two-body features is used, which fails to capture the three-body part as shown in panel (b). On the right hand column, a three-body LODE features are used as well, leading to a model that can also capture the three-body part of the energy.

3. *Fitting the ATM Potential*

A commonly used many-body potential, representing the first correction to the two-body description of dispersion, is the ATM potential, which between three particles labelled as i, j, k is explicitly given by

$$V^{\mathrm{ATM}} = \frac{1 + 3\cos\theta_i \cos\theta_j \cos\theta_k}{(r_{ij} r_{ik} r_{jk})^3}. \tag{S105}$$

We now examine to which extent this ATM potential can be captured exactly for the simple special case in which i, j, k form an isosceles triangle with the common sides meeting at i. In this case, the geometry is specified by two parameters d, the common side length, and $\theta = \theta_i$. The potential can then be written as

$$V^{\mathrm{ATM}}(d, x) = \frac{1}{d^9} \frac{1 + \frac{3}{2}x(1-x)}{(2(1-x))^{3/2}}, \tag{S106}$$

where $x = \cos\theta$. If we wish to fit this using a superposition of the 3-body LODE features as shown in Eq. (S102), we need to only consider terms in which the total decay exponent $p + p' + 2l + 2n + 2n' = 9$, where in general, it is possible to combine coefficients arising from different values of p, n, n'. Since $p, n \geq 0$ (where n is an integer and p is a real number), this provides us with a restriction of the form $2l \leq 9$ which has the solutions $l = 0, 1, 2, 3, 4$, leading to angular dependencies of the form $P_l(\cos\theta) = P_l(x)$ with $x = \cos\theta$ as before. In other words, a general LODE model with a total asymptotic decay of $1/r^9$ can capture any angular dependence that is a linear combination of the first five Legendre polynomials. For the ATM potential which is the leading three-body term, this means to approximate

$$V^{\mathrm{ATM}}(d, x) = \frac{1}{d^9} \frac{1 + \frac{3}{2}x(1-x)}{(2-2x)^{3/2}} \approx \sum_{l=0}^{4} \tilde{w}_l P_l(x) = \sum_{l=0}^{4} w_l x^l \tag{S107}$$

for some fitting coefficients $\tilde{w}_l$ (in terms of the Legendre polynomials) or w_l (in terms of the standard monomials).

In Figure S7, we show the results of this fit (where "best fit" is defined in the $L2$-sense) for ranges of the angle θ: $\theta \in [\pi/4, \pi] = [45°, 180°]$ in panel (a) and $\theta \in [\pi/9, \pi] = [20°, 180°]$ in panel (b). The initial cutoff angle of 45° corresponds to the angle formed at the position of a center atom i, if the two neighbors are at a distance of 4 Å from i and 1.5 Å from each other, thus corresponding to the three-body contribution by a pair of atoms "right outside the cutoff" radius. We can see that if we restrict the range of the angle as shown in (a), we obtain a good fit of the angular dependence of the ATM potential. On the other hand, if we also include narrower angles as shown in (b), we can see that the potential starts to diverge, and the performance of the fit becomes more restricted.

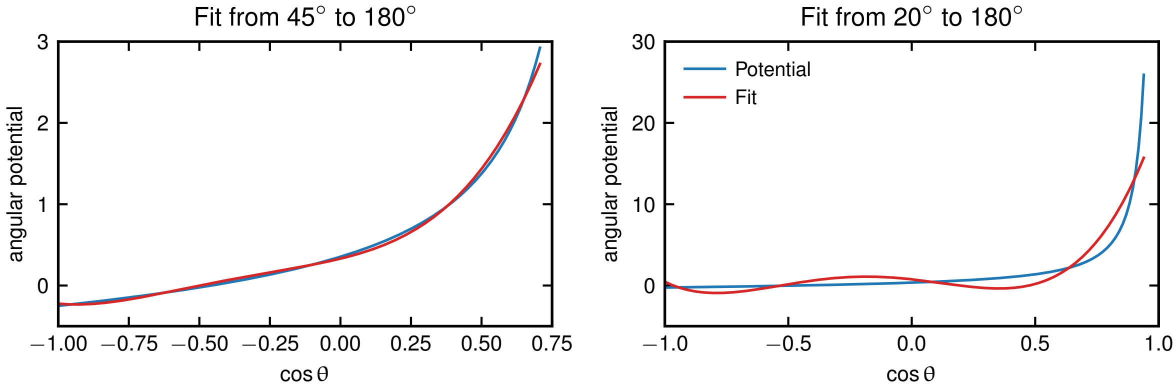

Figure S7. Fitting of angular part of the ATM potential, the equation of which is provided in the text. The fit is performed on two interval ranges for the angle θ: $\theta \in [\pi/4, \pi] = [45^\circ, 180^\circ]$ in panel (a) and $\theta \in [\pi/9, \pi] = [20^\circ, 180^\circ]$ in panel (b). The initial angle of 45° corresponds to the angle formed at the position of a center atom i, if the two neighbors are at a distance of 4 Å from i and 1.5 Å from each other, thus corresponding to the three-body contribution by a pair of atoms "right outside the cutoff" radius.

The difficulty in fitting the divergent part will not be a huge issue in practice due to its origin: it should be noted that this divergence arises from the factor of $1/r_{jk}^3$ as $r_{jk} \to 0$. In other words, as the two atoms "around" atom i approach each other. If the two atoms in practice are closer than the cutoff radius, the SR part of the model would be strongly affected by it, and adapt its coefficients to learn that part of the interaction (at least to the extent that SR models can do that, since such short-distance divergences are also challenging for conventional ML Models).

To make this more explicit: naively, the angle θ at the position i could be small even if the atoms j, k have a more reasonable distance a, if the distance $d \to \infty$ goes to infinity. We however know that for an isosceles triangle, $2a = d \sin\theta/2$ and thus as $d \to \infty$, the angle θ will asymptotically approach $\theta \sim \frac{4a}{d} + \mathcal{O}\left(\left(\frac{a}{d}\right)^3\right)$. In this limit, we then get the asymptotic result

$$V^{\mathrm{ATM}} \sim \frac{1}{d^9} \frac{1}{(\sin\frac{\theta}{2})^{3/2}} \sim \frac{1}{d^6 a^3}. \tag{S108}$$

which is precisely the distance dependence of the ATM potential for three particles forming a triangle with side lengths d, d, a, showing also directly using the angle dependence that there is no true singularity. Furthermore, for $d \to \infty$, the far-field contributions of the comparably quickly decaying $1/r^9$ potential will only constitute a tiny fraction of the total energy. The most dominant part of such a three-body correction would arise from atoms that are right outside of the cutoff radius, and for those, the angle θ will be limited by the small value of d (for instance, two neighbors are at a distance of 4 Å from the center and 1.5 Å from each other would form an angle of 45°, which was the value chosen for Figure S7). This simple toy example shows that the three-body extension of LODE is capable of capturing this interaction decently well.

S5. MODEL DETAILS FOR THE POINT-CHARGE TOY PROBLEM

The toy dataset comprised of a gas of point particles interacting through pure electrostatic or dispersion interactions is built following the procedure discussed in the main text and in Ref. 11. For each structure we compute the atom centered spherical expansion where we use a single angular channel ($l = 0$ and hence automatically $m = 0$), one radial channel for the optimized (monomial) basis, and eight radial channels for the GTO basis. For the short range (SR) model we use a rather large cutoff of 9 Å and set the width of the Gaussian densities to $\sigma = 1$ Å. For the long-range (LR) LODE model we use an environment cut-off 0.1 Å and a Gaussian width of 0.6 Å. In all cases, we predict the total energy of a structure using a decomposition

$$E_{\text{pred}} = \sum_{i=1}^{N} \epsilon_i, \tag{S109}$$

where ϵ_i is the predicted atomic energy for atom i, which is in turn expressed as

$$\epsilon_i = \begin{cases} \sum_{n=0}^{n_{\max}-1} \sum_a w_{na} V_{i,an00} & \text{LODE} \\ \sum_{n=0}^{n_{\max}-1} \sum_a w_{na} \rho_{i,an00} & \text{SR} \end{cases}, \tag{S110}$$

where a runs over all species present in the system, and w_{an} are model weights. We use $n_{\max} = 8$ for the GTO radial basis (used for both SR and LODE features) while $n_{\max} = 1$ for the optimized (monomial) radial basis of LODE.

The weights are optimized by performing a simple ridge regression against the energy, i.e. by minimizing the loss function

$$\mathcal{L}(\boldsymbol{w}) = \sum_A (E(A) - E_{\text{pred}}(A))^2 + \lambda \sum_n w_n^2, \tag{S111}$$

where $E(A)$ is the energy of structure A, $\boldsymbol{w} = (w_0, w_1, \dots)$ is the vector of weights and λ is a regularization parameter. The actual optimization is performed using the implementation in Scikit-learn,[18] with a regularizer of $\lambda = 1 \cdot 10^{-5}$. Given the linear nature of the model, we compute global descriptors of the structure by summing over all atom-centered descriptors within each structure, and apply the regression algorithm at the structure level.

S6. COMPUTATIONAL DETAILS FOR THE DIMER DATASET

As discussed in the main text, we use a dataset based on the BioFragment Database (BFDb),[11,19] that contains rigid-molecule binding curves for pairs of molecular fragments that are charged (C), polar (P) or apolar (A), which gives rise to six classes of dimer interactions. In Table S1 we show some general properties of the dataset while in Table S2 we shows molecular formula and additional information about the monomers appearing in the dataset.

A. Reference energy calculations

We orient the dimers in a periodic cell with a length of 30 Å so that the connecting vector between the COMs of the two fragments points in the $(1, 1, 1)$ direction of the cell. Binding energies and forces are calculated using the Heyd, Scuseria, and Ernzerhof (HSE06)[20] hybrid functional together with a non-local many-body dispersion correction to handle the LR van der Waals (vdW) energy as implemented in the FHI-Aims package.[21] For the HSE06 functional, we split the exchange energy into 75 % Hartree-Fock and treat the remaining 25 % at the PBE level.

Classes	# samples	σ_{energy} meV	σ_{force} meV/ Å
CC	2,392	983.72	246.59
CP	3,471	243.00	131.31
PP	2,093	65.70	39.69
CA	2,730	46.43	25.28
PA	5,434	22.10	7.75
AA	13,663	15.10	3.44
Total	29,783		
Total	29,783		

Table S1. The six classes of dimer interactions, their number of sample in the dataset as well as the standard deviation of the energies and forces of the the training set at a training cutoff of $r_{\text{cut}} = 4$ Å.

Molecular formula	Label	-C	-P	-A
$C_3N_3H_{10}^+$	C	208	26	65
$C_2O_2H_3^-$	C	4,134	1,053	442
$C_2N_3H_8^+$	C	1,729	234	286
C_2H_6	A	780	1,716	22,490
C_2ONH_5	P	416	2,639	455
$C_4N_2H_6$	P	208	1,261	377
C_3ONH_7	P	78	455	195
C_3H_8	A	312	572	4,836
CNH_6^+	C	624	117	0
$C_2OH_6^-$	C	130	949	416
$C_3O_2H_5$	P	806	169	429
$C_5N_2H_8$	P	39	117	39
C_9NH_9	P	0	78	0
COH_4	P	442	1,456	117
C_7H_8	A	182	195	3,770
C_3OH_8	A	78	182	117
C_7OH_8	A	234	1,742	819
C_2NH_8	A	507	39	117
C_4H_{10}	P	52	52	507
$C_5ONH_{11}^-$	C	13	26	0
C_8H_{10}	P	13	0	13
C_8OH_{10}	P	0	13	0

Table S2. Molecular informatoion about monomers together with how many timers they appear in the total dataset with which another monomer class.

B. Empirical interaction exponents

To extract the empirical decay exponents p_{phys} for the interaction energy from the data set we fit each binding curve individually to a linear function on a double logarithmic scale. The linear fit function has the form $f(r) = p_{\text{phys}} \cdot r + b$, where f can either be the energy E or the magnitude of the molecular force $|F_{\text{mol}}|$. The molecular force is defined as the sum of all forces acting on the atoms of each molecule (for a given dimer, the total forces on the two molecules are equal in magnitude and opposite in sign). Figure S8 shows all binding curves of a subset, i.e. CC for charge-charge, as gray solid lines and one representative fit as a solid black line. The mean and standard error of each p_{phys} is given in the legend of each sub-panel. We find that, for every type of interaction, the physical exponent is systematically higher compared to the ideal exponent p_{ideal}. This is an indication of the fact that molecules in the DFT simulation are not ideal point particles as assumed in the derivation of the ideal exponents. Spurious interactions between periodic replicas (especially for charged fragments), as well as noise in the binding energy at large distance (especially for weak interactions) contribute to this discrepancy.

C. SOAP and LODE Hyperparameters

For the SR and the LR spherical expansions we use a cutoff/environment-cutoff of 3.0 Å. For the SR model we use a density Gaussian width of 0.3 Å, and expand the density on a basis including four angular channels, and six GTO-type radial basis channels. The density at the cutoff is smoothed using a shifted cosine switching function with a width of 0.5 Å. For the LR model we either use an optimal monomial basis containing one radial channel and one angular channel, or an extended monomial basis containing six radial and four angular channels, with the exponents determined according to the theoretical analysis in section S2 F. For the LR desciptors we set the width of the Gaussian basis functions to 1 Å and we use no cosine switching function at the cutoff distance. From the atom centered spherical expansion coefficients of the density and the associated potential we construct the $\rho \otimes \rho \oplus \rho \otimes V_p$ multiscale power spectra according to Ref. 11.

D. Linear Models: Separate Dimer Classes

We construct linear-regression models for each type of dimers, using global features obtained by summing over all atom-centered descriptors within each structure, supplemented by composition features that representing the chemical

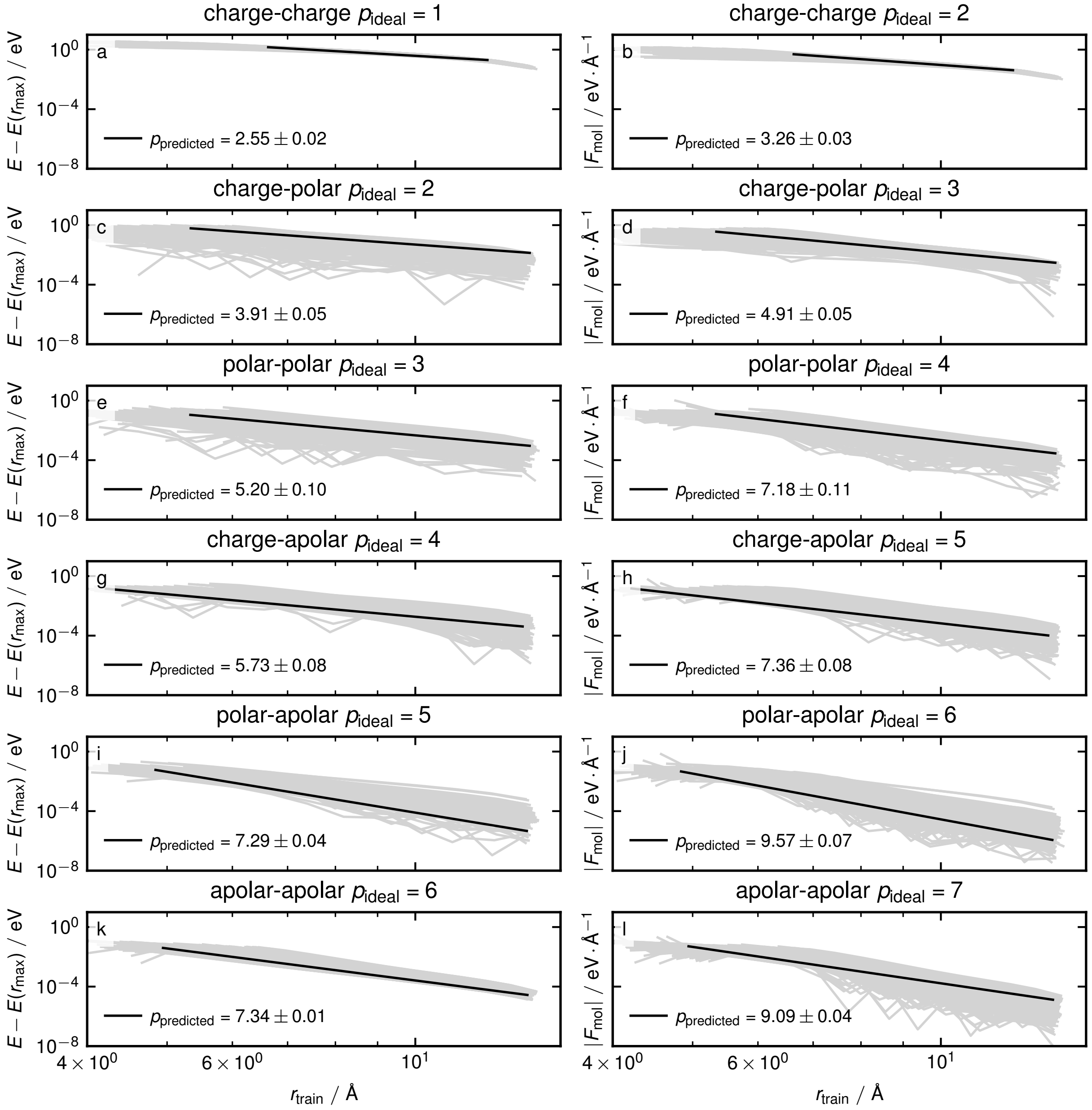

Figure S8. Dimer binding curves on a double logarithmic scale. Gray lines show the binding curves from the DFT simulation and solid black lines shows an example linear fit to a representative binding curve. Values in each figure legend indicate the mean and standard error for each p_{phys}. Each row of sub-panels shows binding curves for different subset of the dimers. Left columns: Binding energies $E - E(r_{\max})$. For a better visualization all curves are shifted by the energy at the maximum separation $E(r_{\max})$. Right columns: Absolute molecular force $|F_{\text{mol}}|$.

stoichiometry of each structure. Explicitly written out, we predict the atomic energy of atom i by

$$\epsilon_i = \begin{cases} \sum_{aa'nn'l} w_{aa'nn'l} I\left[\rho_i \otimes V_i\right]_{aa'nn'l} & \text{LODE} \\ \sum_{aa'nn'l} w_{aa'nn'l} I\left[\rho_i \otimes \rho_i\right]_{aa'nn'l} & \text{SR} \end{cases}, \tag{S112}$$

where $I\left[\rho_i \otimes V_i\right]_{aa'nn'l}$ are the previously mentioned invariants $\sum_m \rho_{i,anlm}\rho_{i,a'n'lm}$ and similarly for $\rho \otimes \rho$ and the total energy is then predicted as the sum of atomic contributions plus an additional species-wise baselining:

$$E_{\text{pred}} = \sum_i \epsilon_i + \sum_a \tilde{\epsilon}_a N_a, \tag{S113}$$

where N_a is the number of atoms of species a, and $\tilde{\epsilon}_a$ is a constant offset in the energy of each such atom. During the linear regression we use a very small regularizer of $2.2 \cdot 10^{-16}$ for the composition features and optimize the regularizer for the SR and LR features using a grid search. The grid is ranges from 10^{-12} to 10^3 and contains 20 equally spaced points. We find the optimal regularizer by minimizing the total RMSE of the energies and forces according to $\mathrm{RMSE} = (\mathrm{RMSE}_{\mathrm{energy}} + \mathrm{RMSE}_{\mathrm{force}})/2$ The linear regression is performed with the equisolve packages maintained at `https://github.com/lab-cosmo/equisolve`. Percentage RMSE are computed by normalizing the absolute RMSE values by the standard deviation of the binding energy of the training structures, computed separately for each dimer subset.

E. Atomic Force Errors

In Figure S9 we compare the accuracy of the atomic force on the test set predicted by linear models using multiple monomials for the radial expansion. Similar to what shown in Figure 3b in the main text we compare different potential exponents $p = 1, 2, \ldots, 9$ and fit separately on each dimer fragment classes. As for the energy we find that all short-range models suffer from poor performance, and the inclusion of any LODE term leads to dramatic improvement in the test accuracy. However, the improvement is less pronounced than for the energies, and there is less-pronounced variation of the accuracy depending on the generalized LODE exponent, even though we find that the best potential exponent roughly corresponds to the ideal exponents for each dimer class.

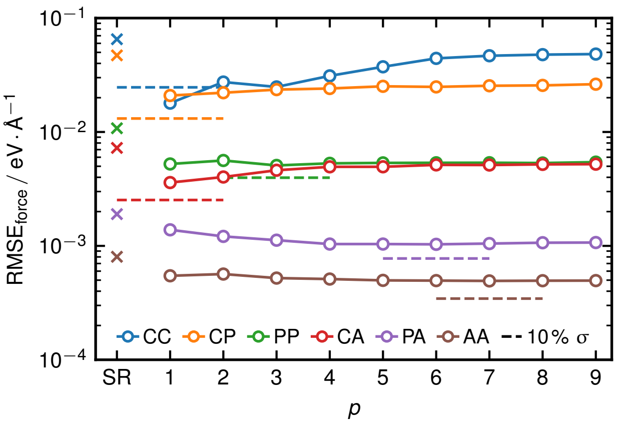

Figure S9. The figure shows the atomic force RMSE as a function of the potential exponent p, similar to Figure 3 in the main text. Dashed horizonatal lines depict the RMSE corresponding to $10\,\%\,\sigma$.

F. Test Errors for Different Training Cutoffs

We recall that the extrapolative learning exercise we perform involves predicting the long-range part of the binding curve, for $r > r_{\mathrm{train}}$, based on training on the part of the data set with $r \leq r_{\mathrm{train}}$ (where distances are defined relative to the minimal separation in the dataset for each dimer configuration). In Figure S10 we show the % RMSE for the energies and forces for different subsets of the dataset as a function of the training cutoff r_{train} and the potential exponent p. We find that an increased training cutoff increases the accuracy on the test set. We note that due to the construction of our train/test split these curves are not usual learning curves. Contrary to the typical training exercises in which the number of test points remains constant, when the training cutoff is increased the number of training points increases while the number of test points decreases. This leads to a flattening of the RMSE curves as a function of the r_{train} for the shorter range interaction types like polar-polar, charge-apolar, polar-apolar and apolar-apolar. For these subsets their binding energy and force is almost zero if the two molecules are further apart then a few Å. Therefore, the learning accuracy will not increase for even larger r_{train} since the whole energy and force can be fully described by the contributions of the isolated individual molecules. We also note that for the force RMSE we find only very little difference between the long and the short range models. This is due to the fact that the force on individual atoms is dominated by the force exerted by nearby atoms, which is usually not zero because of the fact that the binding curves are computed without relaxing the atoms at each interatomic separation.

From this discussion we conclude that the chosen train-test split of $4\,\text{Å}$, which we use in the rest of this work, is a reasonable choice since force curves have not already fully flattened out and the train set is sufficiently large for a physically-inspired model to be able to infer the asymptotic behavior of the interactions, and perform reasonable extrapolative predictions on the test set.

G. Energy Errors for Combined Linear Models Using Non-Charged Fragments

In Figure S11 we show the % RMSE of the energy for models trained on several dimer classes simultaneously. However, instead of training on the whole data set, we chose a subset excluding the charge-charge interactions (!CC, black open circles) or excluding all dimer classes that contain some charged fragment (!C, gray open circles). The SR models show a very poor performance even though the slowest decaying $1/r$ interactions are left out. Comparing the black and the gray open circles we find that the model accuracy increases by a factor of two if all charged molecules are excluded from the model. This originates that for the remaining PP, PA and AP classes the standard deviation of the energies are very similar as we show in the legend of Figure 3b in the main text.

Comparing the potential exponent dependence for the training without the charge-charge fragments we find the best model for potential exponent is $p = 2$. This corresponds to the next slowest decaying interaction after CC, and therefore the largest contribution to the energy, that stems from the charge-polar fragments. For the training on the subset without any charged fragments we find a minimum around $p = 4$ which roughly corresponds to the

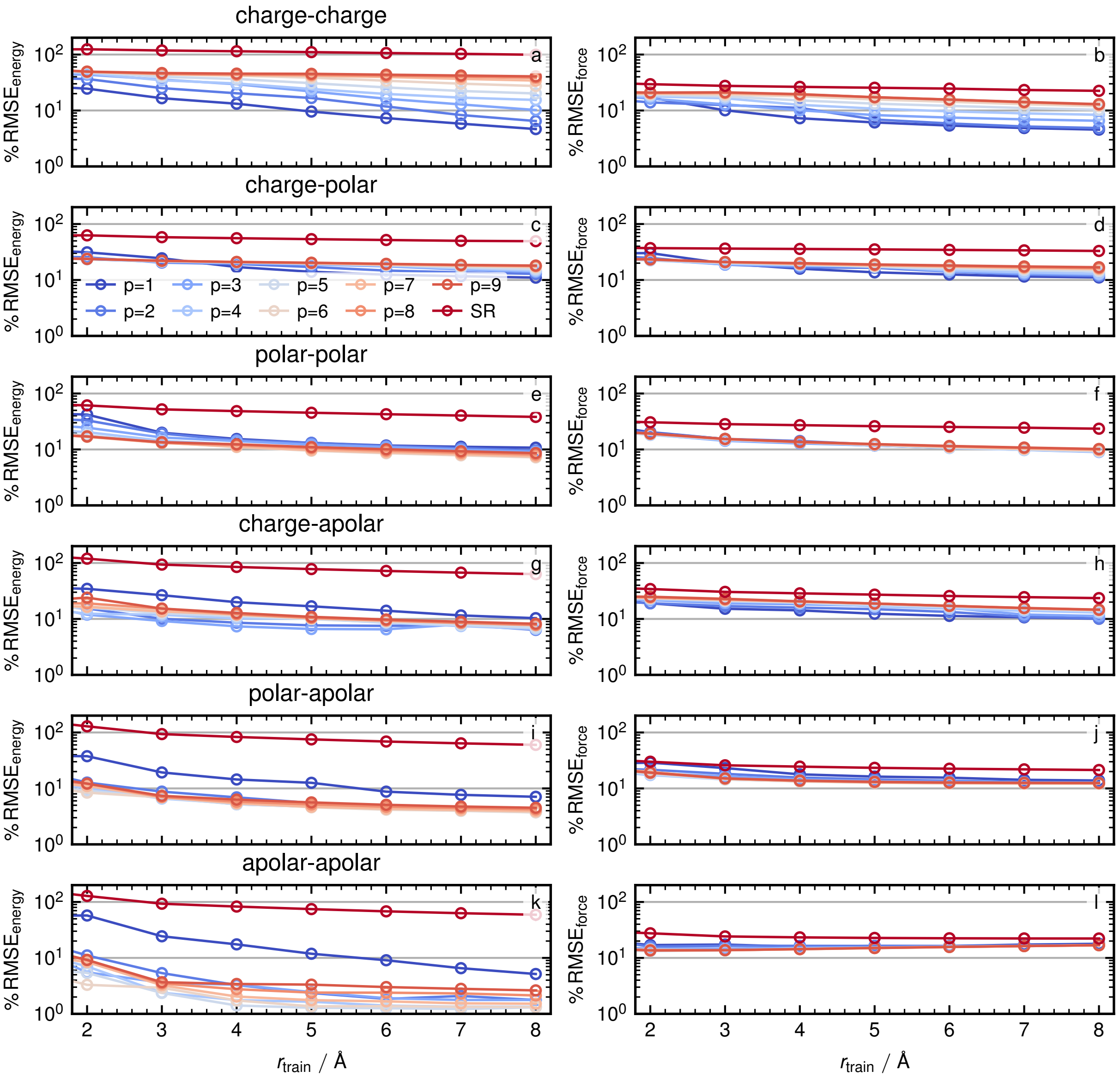

Figure S10. % RMSE for energies and forces for different dimer classes as a function of the training cutoff r_{train} and the potential exponent p. Subpanel titles indicate the corresponding dimer class. Left columns show $\%\,\mathrm{RMSE}_{\mathrm{energy}}$ while right columns show $\%\,\mathrm{RMSE}_{\mathrm{force}}$. Different colors indicate different potential exponents p used in the generalized LODE descriptors.

ideal potential exponent of polar-polar interaction, which is 3. In addition, we find that the model accuracy is nearly independent in the range from $p = 2$ to $p = 6$, which is the range of the potential exponents for the PP, PA, and AA pairs. The weak dependence of the accuracy on the exponent for this subset is consistent with the similar weak dependence seen for the separate training exercises, and reflects the fact that (1) these weak interactions have a similar range of variability, so there is little gain in using an exponent optimized for one of the classes, and (2) the weaker interaction often show pronounced deviations from the ideal behavior (Figure S8).

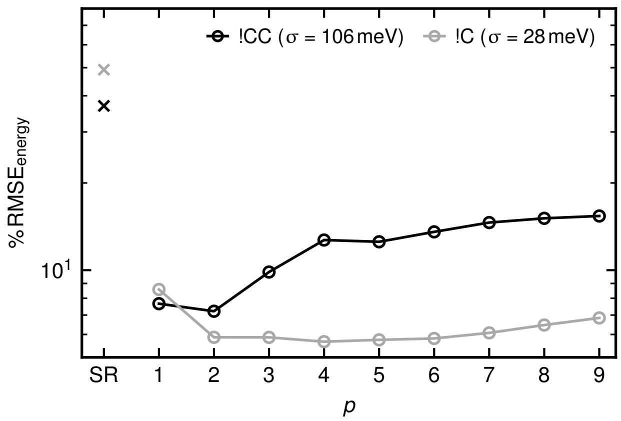

Figure S11. The figure shows the energy % RMSE for models trained on the energies of all dimer structures, up to a cutoff $r_{\mathrm{train}} = 4$ Å, leaving out the CC fragments (!CC, in black) or leaving out all the dimers containing a charged residue (!C, in gray, including only PP, PA, AA dimers). The model details and training protocol are analogous to those used for Figure 3 in the main text.

H. Details of the Neural Network Models

We apply a multilayer perceptron neural network (NN) model to the concatenation of SR and LR descriptors at the level of individual environments, summing over the environments to determine the total energy of each structure, that is used as the target property. The NN uses three hidden layers each with 16 neurons. We use a sigmoid linear unit (SiLU) as activation function and perform a layer normalization for each layer. All models were trained using the Adam optimizer[22] as implemented in pytorch.[23] The learning rate is reduced by a factor of 0.8 every 1000 epochs if the validation loss reaches a plateau. Means and errors of the RMSE's are obtained from five independent training runs. In Figure S12 we show a bar plot analogous to Figure 4 in the main text, displaying the energy RMSE on the train set for energies and forces for the neural network (NN) models. The figure shows that the additional flexibility afforded by a NN architecture allows to improve the accuracy in the interpolative regime, but is not able to improve the extrapolative performance, that requires a physical prior.

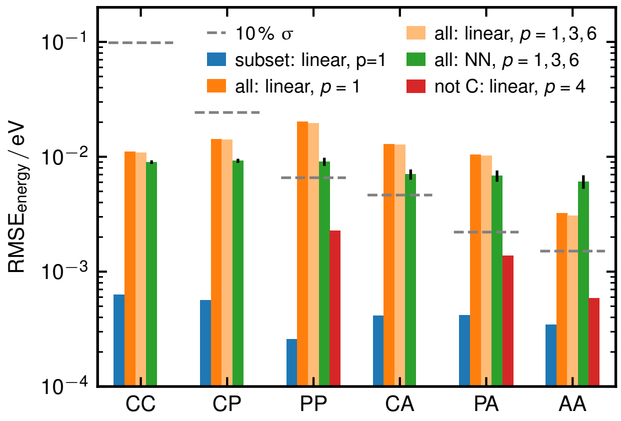

Figure S12. Energy RMSE on the train set for the energies for the different subsets of the dimers. The vertical scale and color coding match those used in Figure 4 in the main text. Blue bars show the RMSE of models using a single $p = 1$ LODE exponent. Orange and light orange bars show linear models fit to the whole data set. Green bars show a fit to the whole data set with a non-linear neural network model. Red bars correspond to a linear model restricted to non-charged fragments. Horizontal gray lines depict an relative error of 10% σ for each subset.

I. Relative Energy Errors for Different Subsets

In Figure S13a we plot the relative RMSE in % with respect to the standard deviation in the energies of the training data based on the data of Figure 4 in the main text which shows the error on the test set. Figure S13b shows the relative error on the training set based on the results shown in Figure S12. The figure shows that for a training on the whole dataset the absolute errors on all subsets are comparable, and the main challenge in improving the relative accuracy for the weaker interactions is due the the discrepancy between the energy scale of different long-range effects.

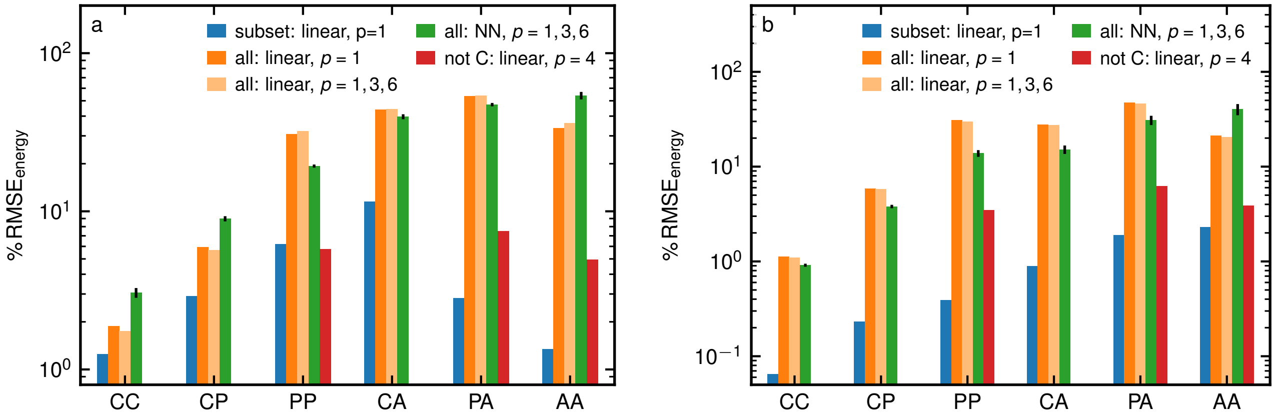

Figure S13. The subsets relative RMSE on the test (a) and the train (b) set for the energies. Blue bars show the RMSE of models using a single $p = 1$ LODE exponent Orange and light orange bars show linear models fit to the whole data set. Green bars show a fit to the whole data set with a non-linear neural network model. Red bars correspond to a linear model restricted to non-charged fragments.

S7. FLEXIBILITY OF POWER-LAW FITS

In this section we show a simple example to illustrate how, given a sufficiently flexible functional form, different types of asymptotic behavior can be achieved using a single potential exponent for the LR features. We consider the following toy model in one dimension: We aim to fit a target function $f(x) = 1/|x|^p$ for fixed p. To do so, we use 6 “atoms” with x coordinates $x \in (-2.5, -1.3, -0.4, 0.5, 1.4, 2.2)$, i.e. atoms around the origin with average distance on the order of 1 roughly corresponding to interatomic distances in Å. We then assume that each of the $i = 1, \ldots, 6$ points generates a $1/|x|^{p'}$ potential, where $p' \neq p$ is different from the true exponent, and with “charges” q_i that can be fitted to the data. Thus, we obtain the fitting function

$$\tilde{f}(x) = \sum_{i=1}^{6} \frac{q_i}{|x - x_i|^{p'}}. \tag{S114}$$

In Figure S14 a and b, we try to fit a true underlying function $f(x) = 1/|x|$ (i.e. $p = 1$) using a superposition of six $1/|x|^3$ functions (i.e. $p' = 3$). We compare the true function (black solid line) against the obtained least squares fit (red dashed line), where the fit was performed such as to minimize the squared error

$$\text{Loss} = \int_{x_{\min}}^{x_{\max}} |f(x) - \tilde{f}(x)|^2 \,\mathrm{d}x, \tag{S115}$$

where the fitting interval was chosen to be $[x_{\min}, x_{\max}] = [5, 10]$.

In Figure S14a, we can see that in this fitting interval, the six $1/|x|^3$ functions lead to an essentially perfect fit of the true underlying $1/|x|$ potential. However, as we move to figure b, and observe how the fit performs against the true behavior for distances beyond the fitting interval, we can see that fit is not truly capturing the LR $1/|x|$ behavior. In fact, as is expected, the asymptotic behavior of $\tilde{f}$ as $x \to \infty$ does indeed approach a $1/|x|^3$ form matching the terms in the fitting function.

In Figure S14c and d, we repeat the same exercise but with exponents flipped: the true underlying function (black solid line) is given by $f(x) = 1/|x|^3$, which is fitted by the superposition of six functions decaying as $1/|x|$ (red dashed line). We can again see that the fit is excellent in the fitting interval $x = [5, 10]$ (Figure S14c). A key difference to the previous case, however, is that also in the extrapolative regime d, the fit still remains accurate up to significantly larger distances. In fact, as was shown in much more generality in section S1, it is possible for a superposition of $1/|x|$ potentials to generate a function with an asymptotic behavior of $1/|x|^3$. To provide a simpler example that only

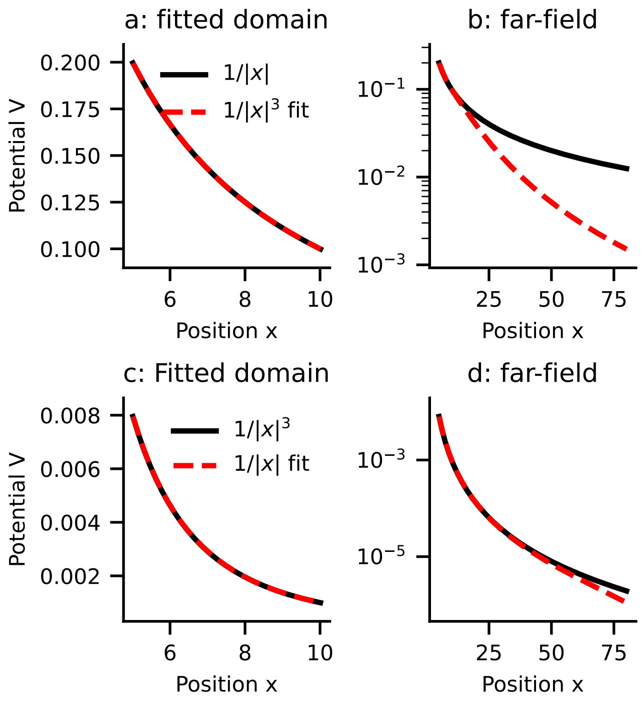

Figure S14. Fitting power law decaying potential with different exponents. Black line shows the to be fitted power law $1/r$ and red dashed line show a fit with a $1/r^3$ power law.

requires knowledge about the geometric series, we can use that for $x \to +\infty$

$$\frac{1}{|x \pm a|} = \frac{1}{x \pm a} = \frac{1}{x}\frac{1}{1 \pm \frac{a}{x}} = \frac{1}{x}\left(1 \mp \frac{a}{x} + \frac{a^2}{x^2} \mp \frac{a^3}{x^4} +\right) = \frac{1}{x} \mp \frac{a}{x^2} + \frac{a^2}{x^3} \mp \frac{a^3}{x^4} + \dots \tag{S116}$$

and thus

$$\frac{1}{|x+a|} + \frac{1}{|x-a|} = 2\frac{a}{x^2} + 2\frac{a^3}{x^4} + \dots \tag{S117}$$

showing that a superposition of two functions decaying as $1/|x|$ can indeed generate a function decaying as $1/|x|^2$, if the relative weights (here, we assumed that the two potentials have the same "charge" / prefactor) and offsets (here: $+a$ and $-a$) lead to a perfect cancellation of the leading $1/|x|$ term. Superpositions of three such functions can then in principle lead to asymptotic $1/|x|^3$ behavior, etc. While possible in principle, this requires a perfect cancellation of terms, which is why the fit in Figure S14(d) does deviate from the perfect $1/|x|^3$ behavior at very large distances.

In summary, we can see that restricted to intermediate distances, one can in principle fit any exponent p from any other reasonably close exponent p', which does not mean that the true far-field behavior is captured. This lack of extrapolation capability for large distances can either be due to fundamental limitations, namely if $p < p'$, or due to the high numerical accuracy that is required to obtain a perfect cancellation of leading order terms for $p > p'$.

We can also flip the story around: if we are only given a superposition of $1/|x|^{p'}$ potentials, e.g. the red dashed lines in the figures S14a and c, and want to find the best $q/|x|^p$ fit for suitable parameters q and p, the exponent we obtain from the fit will typically not be the correct exponent p' unless we can work in the true $x \to \infty$ limit and no cancellations as discussed above happen (which, in the absence of special symmetries, should only occur with a probability of zero). This shows how the very concept of an "exponent" characterizing a potential can be quite subtle when working with dense systems and a limited dataset.

S8. IMPLEMENTATION

We briefly summarize the key ideas that have been used for the practical implementation of the method. If $g(\boldsymbol{r})$ denotes the density contribution function as introduced in section S2, the total potential at a point $\boldsymbol{r}$ can be computed as

$$V(\boldsymbol{r}) = \sum_j g(\boldsymbol{r} - \boldsymbol{r}_j) \tag{S118}$$

The main implementation assumes the use of periodic boundary conditions. In this case, we let j run over all atoms in the primary cell, and add an additional summation over periodic images

$$V(\boldsymbol{r}) = \sum_j \sum_{\boldsymbol{s}} g(\boldsymbol{r} - \boldsymbol{r}_j + \boldsymbol{s}), \tag{S119}$$

where $\boldsymbol{s}$ runs over all lattice sites. The Poisson summation formula is a technique that allows us to compute the periodic sum of a function f defined on $\mathbb{R}^d$ using an analogous sum in reciprocal space, namely

$$\sum_{\boldsymbol{s}} f(\boldsymbol{r} + \boldsymbol{s}) = \frac{1}{\Omega} \sum_{\boldsymbol{k}} \hat{f}(\boldsymbol{k}) e^{i\boldsymbol{k}\boldsymbol{r}}, \tag{S120}$$

where Ω is the volume of the unit cell, $\boldsymbol{k}$ runs over all reciprocal lattice vectors and the Fourier transform is defined as

$$\hat{f}(\boldsymbol{k}) = \int_{\mathbb{R}^3} \mathrm{d}^3\boldsymbol{r}\, e^{-i\boldsymbol{k}\boldsymbol{r}} f(\boldsymbol{r}). \tag{S121}$$

Applying this relation to the evaluation of V, we obtain

$$V(\boldsymbol{r}) = \frac{1}{\Omega} \sum_j \sum_{\boldsymbol{k} \neq 0} \hat{g}(k) e^{i\boldsymbol{k}(\boldsymbol{r} - \boldsymbol{r}_j)}, \tag{S122}$$

where we used (1) the fact that $g(\boldsymbol{r})$ is typically a spherically symmetric function, which then also has a spherically symmetric Fourier transform $g(\boldsymbol{k}) = g(k)$, and (2) to enforce global charge neutrality, we set $\hat{g}(0) = 0$, which is physically equivalent to adding a homogeneous background charge (if necessary). The atom centered density $V_i(\boldsymbol{r}) = V(\boldsymbol{r} + \boldsymbol{r}_i)$ is then given by

$$V_i(\boldsymbol{r}) = \frac{1}{\Omega} \sum_j \sum_{\boldsymbol{k} \neq 0} \hat{g}(k) e^{i\boldsymbol{k}(\boldsymbol{r} - \boldsymbol{r}_{ij})}, \tag{S123}$$

where we defined $\boldsymbol{r}_{ij} = \boldsymbol{r}_j - \boldsymbol{r}_i$ as the relative position of atom j in the coordinate system centered around atom i. To obtain the LODE coefficients $V_{i,nlm}$, we then need to evaluate the integral

$$V_{i,nlm} = \int_0^{r_\mathrm{cut}} \mathrm{d}^3\boldsymbol{r}\, R_{nl}(r) Y_l^m(\hat{\boldsymbol{r}}) V_i(\boldsymbol{r}) \tag{S124}$$

$$= \sum_j \sum_{\boldsymbol{k} \neq 0} \hat{g}(k) e^{-i\boldsymbol{k}\boldsymbol{r}_{ij}} \int_0^{r_\mathrm{cut}} \mathrm{d}^3\boldsymbol{r}\, R_{nl}(r) Y_l^m(\hat{\boldsymbol{r}}) V_i(\boldsymbol{r}) e^{i\boldsymbol{k}\boldsymbol{r}}, \tag{S125}$$

where in the last step, we used the fact that V_i is continuous, which then justifies interchanging the order of the infinite summation over $\boldsymbol{k}$ and the integral. To evaluate the integral, it is necessary to use the plane-wave expansion theorem, which states that

$$e^{i\boldsymbol{k}\boldsymbol{r}} = 4\pi \sum_{l,m} i^l Y_l^m(\hat{\boldsymbol{k}}) Y_l^m(\hat{\boldsymbol{r}}) j_l(kr), \tag{S126}$$

where we use real spherical harmonics Y_l^m, j_l is the spherical Bessel function of the first kind, $k = \|\boldsymbol{k}\|$ is the norm and $\hat{\boldsymbol{k}} = \boldsymbol{k}/k$ the direction of $\boldsymbol{k}$. Using the orthogonality of the spherical harmonics, we thus get

$$\int_0^{r_\mathrm{cut}} \mathrm{d}^3\boldsymbol{r}\, R_{nl}(r) Y_l^m(\hat{\boldsymbol{r}}) V_i(\boldsymbol{r}) e^{i\boldsymbol{k}\boldsymbol{r}} = 4\pi i^l Y_l^m(\hat{\boldsymbol{k}}) \int_0^{r_\mathrm{cut}} \mathrm{d}r\, r^2 R_{nl}(r) j_l(kr) \tag{S127}$$

Thus, with the definition

$$\tilde{I}_{nl}(k) = \int_0^{r_{\rm cut}} \mathrm{d}r\, r^2 R_{nl}(r) j_l(kr), \tag{S128}$$

we obtain

$$V_{i,nlm} = \frac{4\pi}{\Omega} i^l \sum_j \sum_{\boldsymbol{k}\neq 0} \hat{g}(k) e^{-i\boldsymbol{k}\boldsymbol{r}_{ij}} \tilde{I}_{nl}(k) Y_l^m(\hat{\boldsymbol{k}}). \tag{S129}$$

While the current expression still contains a factor of i^l, seemingly making the coefficients complex, $V_{i,nlm}$ should in fact be a real number. To see how, it is helpful to group the wave vectors $\boldsymbol{k}$ into pairs $(\boldsymbol{k}, -\boldsymbol{k})$. Then, using the fact that $Y_l^m(-\boldsymbol{k}) = (-1)^l Y_l^m(\boldsymbol{k})$, we get

$$e^{-i\boldsymbol{k}\boldsymbol{r}_{ij}} Y_l^m(\boldsymbol{k}) + e^{+i\boldsymbol{k}\boldsymbol{r}_{ij}} Y_l^m(-\boldsymbol{k}) = \left(e^{-i\boldsymbol{k}\boldsymbol{r}_{ij}} + (-1)^l e^{+i\boldsymbol{k}\boldsymbol{r}_{ij}}\right) Y_l^m(\boldsymbol{k}) \tag{S130}$$

$$= Y_l^m(\boldsymbol{k}) \cdot \begin{cases} 2\cos(\boldsymbol{k}\boldsymbol{r}_{ij}) & l \text{ even} \\ -2i\sin(\boldsymbol{k}\boldsymbol{r}_{ij}) & l \text{ odd} \end{cases} \tag{S131}$$

Combined with the extra prefactor of i^l, we then get the more clearly real-valued expressions

$$V_{i,nlm} = \frac{8\pi}{\Omega} \sum_j \sum_{\boldsymbol{k}}{}' \hat{g}(k) \tilde{I}_{nl}(k) Y_l^m(\hat{\boldsymbol{k}}) \begin{cases} (-1)^{\frac{l}{2}} \cos(\boldsymbol{k}\boldsymbol{r}_{ij}) & l \text{ even} \\ (-1)^{\lfloor \frac{l}{2} \rfloor} \sin(\boldsymbol{k}\boldsymbol{r}_{ij}) & l \text{ odd} \end{cases}, \tag{S132}$$

where the prime in $\sum_{\boldsymbol{k}}'$ indicates that the sum only runs over one representative out of each pair of reciprocal space vectors $(\boldsymbol{k}, -\boldsymbol{k})$.

Evaluating this expression requires a sum over N atoms j, as well as an infinite reciprocal space sum over $\boldsymbol{k}$. In practice, the sum over $\boldsymbol{k}$ is truncated by choosing a typical length scale σ that determines the resolution in space of the Fourier approximation. The number of terms in the sum over $\boldsymbol{k}$ is then proportional to the system volume Ω. Since this operation naively needs to be repeated for each center atom i, the total computational cost then scales as $\mathcal{O}(N^2\Omega)$, which for condensed systems with $\Omega \propto N$ becomes $\mathcal{O}(N^3)$, at least naively. However, by using

$$\cos(\boldsymbol{k}\boldsymbol{r}_{ij}) = \cos\left[\boldsymbol{k}(\boldsymbol{r}_j - \boldsymbol{r}_i)\right] = \cos(\boldsymbol{k}\boldsymbol{r}_i)\cos(\boldsymbol{k}\boldsymbol{r}_j) + \sin(\boldsymbol{k}\boldsymbol{r}_i)\sin(\boldsymbol{k}\boldsymbol{r}_j) \tag{S133}$$

and the analogous transformation for $\sin(\boldsymbol{k}\boldsymbol{r}_{ij})$, one can precompute all combinations of the form

$$C(\boldsymbol{k}) = \sum_j \cos(\boldsymbol{k}\boldsymbol{r}_j) \tag{S134}$$

$$S(\boldsymbol{k}) = \sum_j \sin(\boldsymbol{k}\boldsymbol{r}_j) \tag{S135}$$

in $\mathcal{O}(N^2)$ operations, reducing the computational cost for all LODE coefficients in the structure to $\mathcal{O}(N^2)$. Using the fast Fourier transform, it is further possible to reduce the cost to $\mathcal{O}(N \log N)$.

Gradients can be implemented straight-forwardly by differentiating the above exression with respect to some $\boldsymbol{r}_j$:

$$\frac{\partial V_{i,nlm}}{\partial \boldsymbol{r}_j} = \frac{8\pi}{\Omega} \sum_{\boldsymbol{k}}{}' \hat{g}(k) \tilde{I}_{nl}(k) \boldsymbol{k} Y_l^m(\hat{\boldsymbol{k}}) \begin{cases} -(-1)^{\frac{l}{2}} \sin(\boldsymbol{k}\boldsymbol{r}_{ij}) & l \text{ even} \\ (-1)^{\lfloor \frac{l}{2} \rfloor} \cos(\boldsymbol{k}\boldsymbol{r}_{ij}) & l \text{ odd} \end{cases} \tag{S136}$$

In this case, since all pairs i, j require a sum over $\boldsymbol{k}$, there is an associated memory cost of $\mathcal{O}(N^2)$ and computational cost of $\mathcal{O}(N^2\Omega) \sim \mathcal{O}(N^3)$ to evaluate all such gradient components for an entire structure containing N atoms.

---

(1) Jackson, J. D. *Classical Electrodynamics Third Edition*, 3rd ed.; Wiley: New York, 1998.

(2) Musil, F.; Grisafi, A.; Bartók, A. P.; Ortner, C.; Csányi, G.; Ceriotti, M. Physics-Inspired Structural Representations for Molecules and Materials. *Chem. Rev.* **2021**, *121*, 9759–9815.

(3) Bartók, A. P.; Payne, M. C.; Kondor, R.; Csányi, G. Gaussian Approximation Potentials: The Accuracy of Quantum Mechanics, without the Electrons. *Phys. Rev. Lett.* **2010**, *104*, 136403.

(4) Bartók, A. P.; Kondor, R.; Csányi, G. On Representing Chemical Environments. *Phys. Rev. B* **2013**, *87*, 184115.
(5) Behler, J.; Parrinello, M. Generalized Neural-Network Representation of High-Dimensional Potential-Energy Surfaces. *Phys. Rev. Lett.* **2007**, *98*, 146401.
(6) Drautz, R. Atomic Cluster Expansion for Accurate and Transferable Interatomic Potentials. *Phys. Rev. B* **2019**, *99*, 014104.
(7) Grisafi, A.; Ceriotti, M. Incorporating Long-Range Physics in Atomic-Scale Machine Learning. *J. Chem. Phys.* **2019**, *151*, 204105.
(8) Nijboer, B. R. A.; De Wette, F. W. On the Calculation of Lattice Sums. *Physica* **1957**, *23*, 309–321.
(9) Williams, D. E. Accelerated Convergence of Crystal-Lattice Potential Sums. *Acta Crystallographica Section A: Crystal Physics, Diffraction, Theoretical and General Crystallography* **1971**, *27*, 452–455.
(10) Williams, D. E. Accelerated Convergence Treatment of R-n Lattice Sums. *Crystallography Reviews* **1989**, *2*, 3–23.
(11) Grisafi, A.; Nigam, J.; Ceriotti, M. Multi-Scale Approach for the Prediction of Atomic Scale Properties. *Chem. Sci.* **2021**, *12*, 2078–2090.
(12) Grisafi, A.; Wilkins, D. M.; Csányi, G.; Ceriotti, M. Symmetry-Adapted Machine Learning for Tensorial Properties of Atomistic Systems. *Phys. Rev. Lett.* **2018**, *120*, 036002.
(13) Behler, J.; Parrinello, M. Generalized Neural-Network Representation of High-Dimensional Potential-Energy Surfaces. *Phys. Rev. Lett.* **2007**, *98*, 146401.
(14) Nigam, J.; Willatt, M. J.; Ceriotti, M. Equivariant Representations for Molecular Hamiltonians and N-center Atomic-Scale Properties. *J. Chem. Phys.* **2022**, *156*, 014115.
(15) Thompson, A.; Swiler, L.; Trott, C.; Foiles, S.; Tucker, G. Spectral Neighbor Analysis Method for Automated Generation of Quantum-Accurate Interatomic Potentials. *Journal of Computational Physics* **2015**, *285*, 316–330.
(16) Willatt, M. J.; Musil, F.; Ceriotti, M. Atom-Density Representations for Machine Learning. *J. Chem. Phys.* **2019**, *150*, 154110.
(17) Nigam, J.; Pozdnyakov, S.; Ceriotti, M. Recursive Evaluation and Iterative Contraction of N -Body Equivariant Features. *J. Chem. Phys.* **2020**, *153*, 121101.
(18) Pedregosa, F. et al. Scikit-Learn: Machine Learning in Python. *Journal of Machine Learning Research* **2011**, *12*, 2825–2830.
(19) Burns, L. A.; Faver, J. C.; Zheng, Z.; Marshall, M. S.; Smith, D. G. A.; Vanommeslaeghe, K.; MacKerell, A. D.; Merz, K. M.; Sherrill, C. D. The BioFragment Database (BFDb): An Open-Data Platform for Computational Chemistry Analysis of Noncovalent Interactions. *J. Chem. Phys.* **2017**, *147*, 161727.
(20) Heyd, J.; Scuseria, G. E.; Ernzerhof, M. Hybrid Functionals Based on a Screened Coulomb Potential. *J. Chem. Phys.* **2003**, *118*, 8207–8215.
(21) Blum, V.; Gehrke, R.; Hanke, F.; Havu, P.; Havu, V.; Ren, X.; Reuter, K.; Scheffler, M. Ab Initio Molecular Simulations with Numeric Atom-Centered Orbitals. *Computer Physics Communications* **2009**, *180*, 2175–2196.
(22) Kingma, D. P.; Ba, J. Adam: A Method for Stochastic Optimization. 2017.
(23) Paszke, A. et al. PyTorch: An Imperative Style, High-Performance Deep Learning Library. 2019.
