# Supplementary material for: Physics-Inspired Equivariant Descriptors of Nonbonded Interactions
Source: J Phys Chem Lett. 2023 Oct 20;14(43):9612–8. doi: 10.1021/acs.jpclett.3c02375 (PMC10626632; doi:10.1021/acs.jpclett.3c02375)
Supplement: Supplementary file 3 — jz3c02375_si_003.pdf [file jz3c02375_si_003.pdf]

jz-2023-02375h.R1

Name: Peer Review Information for "Physics-inspired Equivariant Descriptors of Non-bonded Interactions"

First Round of Reviewer Comments

Reviewer: 1

Comments to the Author

The manuscript describes the extension of the LODE framework, originally constructed for charge-charge interactions, to arbitrary asymptotics. The mathematical details are delegated to the SI, and the paper mostly presents benchmark results of a toy system and dimer interactions. The aim of the work is to keep the flexibility of machine learned methods while introducing physical behaviour in otherwise extrapolative regimes, where ML performs poorly. This aim is partially achieved, with an incomplete reproduction of long-range asymptotics in the dimer test set. The absolute errors for some systems are very respectable, but interestingly in the charge-charge dimers are quite poorly the long-distance interaction appears somewhat inaccurate. It is unclear from the manuscript if the authors tried a combination of p models, as my understanding is that a single p model only represents the leading term of the asymptotics, but other may be also important. I have missed some even simpler "toy models" from the evaluation, e.g. the 3-body component of the many-body dispersion of 3 particles could be beautifully analysed with this extension of LODE.

I also had the impression that the text could be improved at many places. The language is colloquial (see below for examples), and some of the commentary could be much clearer.

I am somewhat surprised by the argument of V in Eq. 1: why (r+r_i)? It may be obvious to others, but an explanation would be useful to some of us. The SI doesn't appear to explain this specifically.

Figures and figure captions should be made clearer. Most adeptly summarised by the authors themselves, who, presumably accidentally, left the comment "I find this a little confusing" in the caption of Fig. 3. I couldn't agree more. E.g. Fig.2, the caption should explicitly say "panel a" rather than "a" as one can confuse "a" with the English definite article "a". At the same place, the authors presumably describe "b" when they say "In a all particles are neutral". Also, legends could be clearer. In Fig. 2, what is n=8? Presumably they mean n_\max = 8? In Fig. 3, "b: Same as a but using multiple monomials for the radial basis" - which exactly? What is encoded in panels c and d by the colours? They are particularly difficult to see in panel c. The legends in Fig. 4 should be explained.

It would be great if the "linear model" expression was defined quantitatively in the text as well. What is r_0 in p4, l55? What is "shifted"? Phrases such as "we perform a different kind of experiments" should be removed or exactly specified.

In this current form I don't see the publication criterion (new physical insights and/or extremely important results) satisfied. The model is a very useful extension of LODE, which could be extremely important tool for modellers, but in this current presentation I did not find new physical insight or novel results. The presentation is also in need of improvement. Being a potentially powerful tool, this may be possible after a major review.

Reviewer: 2

Comments to the Author

In the manuscript "Physics-inspired Equivariant Descriptors of Non-bonded Interactions", the authors build on previous work combining SOAP with LODE, present new results, and discuss the usefulness of extensions to the long-range equivariant framework and the challenges of extending the ML potential to long-range physical interactions.

Minor issues:

The authors should be given credit for making their sophisticated and well-documented piece of rascaline software available on Github. However, it seems that the scripts to generate the training data and to fit the models are not provided and thus the work cannot be reproduced. I believe that it is important to make them available in a single source and ensure that the results are reproducible.

Recommendations:

Fig 1 is central to the introduction. However in its current form together with the purely descriptive explanations included in the caption, it is not very clear. Maybe connections can be made to Eq. 1 and integrated into the figure, to clarify how the potential is constructed.

The mentioned 'specialised methods for general p' (page 2) could be described and the choice discussed shortly for convenience of the reader.

The 'optimised radial basis' should be formally introduced before they are mentioned in the text to be clear about what 'optimised' means.

'we perform a different kind of experiments' (page 4) -experiment

'This setup is consistent with a practical scenario...' - it should be explained more in detail which practical scenarios are referred to here. This would also allow to go into a short discussion of the current capabilities and limitations of the SOAP+LODE framework (computational costs, number of molecules, atomic species etc.).

In Fig 3 the explanation in the caption is hard to understand even after multiple readings.

Fig 4 it might be useful to indicate a percentage error that can be considered acceptable for the dataset e.g. using a horizontal line

Author's Response to Peer Review Comments:

STI – IMX - COSMO
Laboratory of Computational Science and Modelling

Lausanne, 3 Oct 2023

Dear Prof.,

We thank you and the reviewers for their careful consideration, supporting recommendations, and suggestions to improve our study further. We have attached a revised manuscript, highlighting the many changes to the manuscript with blue text.

We clarified figures and exposition, in response to the many constructive comments of both reviewers, and added a brief discussion of how many-body long-range interactions can be described by LODE, complemented by a more detailed section in the SI. We believe this to add a very interesting additional dimension to our results, and to address - together with the clarifications in the analysis of pair interactions - the concerns of Reviewer 1 on the suitability of J. Phys. Chem. Letters. We thank Reviewer 1 for pointing us in this direction.

As requested by Reviewer 2, we also provide scripts to reproduce the benchmarks and experiments discussed in the manuscript. We have also discussed further the physical implications of our observations.

We also adapted the manuscript to conform to the journal guidelines. Only for source code references we have decided to keep them as URLs to Github repositories, since the version control system still allows for reproducibility.

Yours sincerely,

Michele Ceriotti
on behalf of all Authors

EPFL STI-IMX-COSMO
Pr. Michele Ceriotti
MXG 340, Station N° 12

E-mail: michele.ceriotti@epfl.ch

Reviewer #1

The manuscript describes the extension of the LODE framework, originally constructed for charge-charge interactions, to arbitrary asymptotics. The mathematical details are delegated to the SI, and the paper mostly presents benchmark results of a toy system and dimer interactions. The aim of the work is to keep the flexibility of machine learned methods while introducing physical behaviour in otherwise extrapolative regimes, where ML performs poorly.

This aim is partially achieved, with an incomplete reproduction of long-range asymptotics in the dimer test set. The absolute errors for some systems are very respectable, but interestingly in the charge-charge dimers are quite poorly the long-distance interaction appears somewhat inaccurate.

Indeed, in Fig. 3, the relative errors on the dimers of the CC (charged-charged) class do remain relatively high even when using the "correct" exponent $p=1$, while the results are best for the classes AA and PA. At least in part, we believe that this is due to the different number of training structures (2392 CC pairs, 5434 PA, 13663 AA, we added a complete table, table S1, in section S6 of the SI).
Furthermore, it should be noted that we are training on the total energy, which also includes short-ranged contributions. Given that the reported test errors are from the "extrapolative regime" (dimers with small separations are used in training set, while those with big separations are used for the test set), the slowly decaying Coulomb potential would represent a larger fraction of the total energy in general. This is also apparent considering how a model trained on energy alone (as in Fig. 4) has considerably lower RMSE(energy) than a model trained on both energy and forces (as in Fig. 3). We have added a brief discussion about this fact also in the main text.

It is unclear from the manuscript if the authors tried a combination of p models, as my understanding is that a single p model only represents the leading term of the asymptotics, but other may be also important.

We have updated the main text in multiple places to make more clear whether a single or multiple values of p are used. To clarify on the meaning of p: When we say that the dipole-dipole interaction has an exponent of $p=3$, it indeed is implied that this is the leading order term (unless we are talking about ideal dipoles, which is rarely the case in atomistic modeling). Similarly, also for LODE, features built using an exponent $p=3$ would automatically contain terms decaying with an exponent of 4,5,... as well. In this sense, the used features do also match the analogous properties of the target. Furthermore, we did experiment with using a model with multiple p values (light orange and green bars in Fig. 4) but observed only minute improvements, suggesting that at the dataset size we are working, additional model flexibility is not the primary limiting factor.

I have missed some even simpler "toy models" from the evaluation, e.g. the 3-body component of the many-body dispersion of 3 particles could be beautifully analysed with this extension of LODE.

We thank the reviewer for this important suggestion. We took their comment at heart, and performed a detailed analytical study, as well as additional benchmark evaluations for a 3 particle xenon system. The analysis shows that indeed LODE is able to analyze the 3-body component of the many-body dispersion, provided one uses a generalized 3-body version of the descriptors. We have added a paragraph in the main text and a full section S5 in the Supporting Information that discusses the application of LODE to study long-ranged many-body effects, with this simple dataset containing that is used to verify the theoretical results (we also reproduced Fig. S6 below,

for convenience). We have kept the discussion in the main text short, since the main focus of the work still is the meaning of the coefficients themselves (which do originally come from pair interactions, despite being able to describe many-body effects when combined) and the interplay of different interactions, rather than the systematic body-ordered expansions. We believe this example allowed us to touch upon another very important property of the LODE framework, adding an entire new dimension (the body order of the non-bonded interactions) to the manuscript.

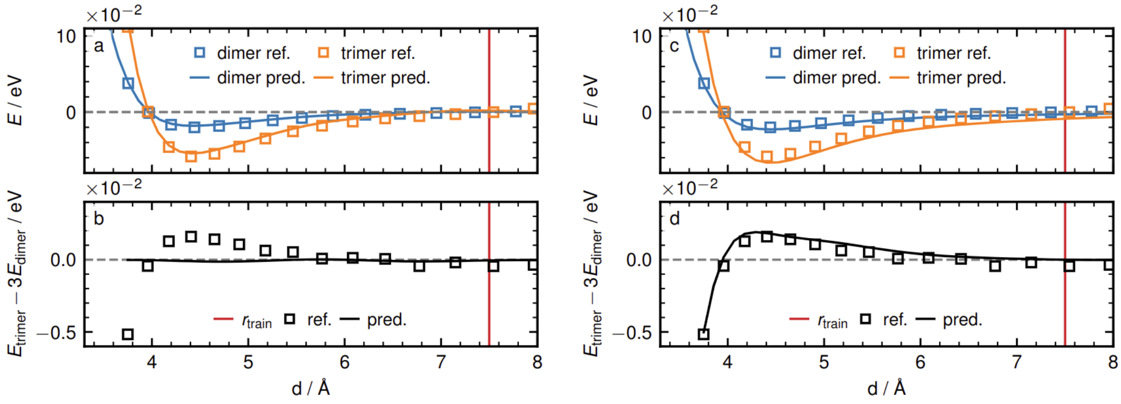

Figure S6. Fitting of three-body dispersion using LODE. The two figures on top (a,c) show the target data and predicted curves for the total energy of both the dimers and trimers as a function of the interatomic distance d. The two figures on the bottom (b,d) show the analogous result for the three-body part of the energy, defined as $E_{3b}(d) = E_{\mathrm{trimer}}(d) - 3E_{\mathrm{dimer}}(d)$. For the left hand column, a linear model only using two-body features is used, which fails to capture the three-body part as shown in panel (b). On the right hand column, a three-body LODE features are used as well, leading to a model that can also capture the three-body part of the energy.

I also had the impression that the text could be improved at many places. The language is colloquial (see below for examples), and some of the commentary could be much clearer.

We have edited the manuscript with the goal of achieving higher clarity, and address the more specific points further below.

I am somewhat surprised by the argument of V in Eq. 1: why (r+r_i)? It may be obvious to others, but an explanation would be useful to some of us. The SI doesn't appear to explain this specifically.

The explanation here might indeed have been lacking. The text around Eqs. (1) and (2) that also uses the same construction has now been modified to explain this more properly. Furthermore, we have updated Fig. 1 that is also relevant to these paragraphs to better support the explanations given in the main text.

Figures and figure captions should be made clearer. Most adeptly summarised by the authors themselves, who, presumably accidentally, left the comment "I find this a little confusing" in the caption of Fig. 3. I couldn't agree more. E.g. Fig.2, the caption should explicitly say "panel a" rather than "a" as one can confuse "a" with the English definite article "a". At the same place, the authors presumably describe "b" when they say "In a all particles are neutral". Also, legends could be clearer. In Fig. 2, what is n=8? Presumably they mean n_\max = 8? In Fig. 3, "b: Same as a but using multiple monomials for the radial basis" - which exactly? What is encoded in panels c and d by the colours? They are particularly difficult to see in panel c. The legends in Fig. 4 should be explained.

We are thankful to the reviewer for reading the manuscript very carefully and catching many of these mistakes, as well as the detailed comments on the contents. We generally agree with the assessment and have now modified the legends of Fig. 3 and 4 as well as the caption of all figures to better convey the key messages. In particular, we have moved from expressing errors in terms of a fraction of the intrinsic variability (which is good to assess how well the model is learning, but hard to interpret in terms of actual energy accuracy) with absolute energy RMSE, using a dashed line at 10% of the intrinsic variability to convey information on the widely different energy scales of the various types of interactions.

It would be great if the "linear model" expression was defined quantitatively in the text as well.

While we agree in spirit, we have decided not to write down the full expression in the main text, and have added a reference to the Supporting Information to sections S5C2 (three-body), S6 (point charges), S7D (dimer binding) instead. This is because the full model expression is more involved due to the atom-centered nature of the features, leading to a slightly more complex model expression (of course, to scientists already working in this field, there is nothing new here, but we believe that the apparent additional complexity might confuse newcomers to the field more).

What is r_0 in p4, l55? What is "shifted"? Phrases such as "we perform a different kind of experiments" should be removed or exactly specified.

r_0 refers to the initial separation of two molecules before increasing their distance along their connecting vector. We acknowledge that the prior statement has been imprecise, and have now written out what is meant more explicitly at the relevant locations.

In this current form I don't see the publication criterion (new physical insights and/or extremely important results) satisfied. The model is a very useful extension of LODE, which could be extremely important tool for modellers, but in this current presentation I did not find new physical insight or novel results. The presentation is also in need of improvement. Being a potentially powerful tool, this may be possible after a major review.

We thank the reviewer again for this critical and constructive feedback about the general scope of our manuscript. We believe that extending LODE to general exponents while also discussing the physical interpretation of the LODE features based on analytical results is crucial. The mathematical analysis provides guidelines for choosing good model parameters, and allows us to have an explicit description of the type of physical interactions that can be captured by these descriptors. On top of the direct use as a modeling tool, some of these insights will also be useful for a wider audience of physicists and physical chemists, because of the general implications for the design of architectures that combine data-driven and physics-based elements. Last, but not least, we believe that the explicit discussion of how LODE features must be generalized to describe many-body long-range interactions, which was prompted by the reviewer’s suggestion, adds a completely different, and novel, dimension to our analysis.
We believe that with our changes we made the novelty clearer and hope that the revised manuscript does now meet the reviewer’s standard.

Reviewer #2

The authors should be given credit for making their sophisticated and well-documented piece of rascaline software available on Github. However, it seems that the scripts to generate the training data and to fit the models are not provided and thus the work cannot be reproduced. I believe that it is important to make them available in a single source and ensure that the results are reproducible

We thank the reviewer for the positive comments and constructive feedback. We have made the scripts, the input for the DFT simulations as well as our datasets publicly available. The locations of these files are referenced at the end of the manuscript.

Fig 1 is central to the introduction. However in its current form together with the purely descriptive explanations included in the caption, it is not very clear. Maybe connections can be made to Eq. 1 and integrated into the figure, to clarify how the potential is constructed.

We agree that the figure was perhaps more geared towards pleasing aesthetics over supporting the content of the main text. We now use an improved version of the figure as the toc figure and replaced Fig. 1 with a new one, that follows closely the steps mentioned in the main text on the construction of the LODE coefficients and that provides a more pedagogic description of the nature of the LODE construction.

The mentioned 'specialised methods for general p' (page 2) could be described and the choice discussed shortly for convenience of the reader.

We have added a brief description of the general idea in the main text, with a targeted reference to the relevant section in the supporting information for readers interested in more details.

The 'optimised radial basis' should be formally introduced before they are mentioned in the text to be clear about what 'optimised' means.

We have updated the main text in a way that clearly mentions that the optimal basis is referring to the monomial basis introduced in the same paragraph, and explains what justifies the term “optimized”.

'we perform a different kind of experiments' (page 4) -experiment

We have fixed the typo and also modified the phrasing more in light of the comments by reviewer #1.

'This setup is consistent with a practical scenario...' - it should be explained more in detail which practical scenarios are referred to here. This would also allow to go into a short discussion of the current capabilities and limitations of the SOAP+LODE framework (computational costs, number of molecules, atomic species etc.).

We have modified the text to more clearly explain what scenarios are meant here: we typically want to train models on small structures (i.e. containing a small number of atoms), and apply them to large structures which are no longer accessible to quantum mechanical methods.
There already was a discussion on the limitations, which however is located at a later stage in the text. Since we believe that it is more fitting to have this discussion after presenting the results, we have kept the location of the latter.

In Fig 3 the explanation in the caption is hard to understand even after multiple readings. Fig 4 it might be useful to indicate a percentage error that can be considered acceptable for the dataset e.g. using a horizontal line

We thank the reviewer for his comments about the figures. Also following criticism from Reviewer 1, we have re-designed both figures to show the absolute errors rather than the errors relative to the intrinsic variability of the various classes of dimers. We have also rewritten the captions, hopefully making these information-rich figures easier to interpret. We do not want to indicate an “acceptable error” (and we have removed one occurrence of this phrasing in the conclusions), as what is acceptable depends on the application. However, we have added horizontal lines on both Fig. 3 and 4 that correspond to 10% of the intrinsic variability of each subset. These allow a reader to appreciate the widely different energy scales, and to gauge how the absolute error compares with these energy scales. Informally speaking, these can also indicate the order of magnitude of an “acceptable” level of error for each type of interaction, if one were forced to indicate one.
